# Genomic saturation mutagenesis and polygenic analysis identify novel yeast genes affecting ethyl acetate production, a non-selectable polygenic trait

**DOI:** 10.15698/mic2016.04.491

**Published:** 2016-03-18

**Authors:** Tom Den Abt, Ben Souffriau, Maria R. Foulquié-Moreno, Jorge Duitama, Johan M. Thevelein

**Affiliations:** 1 Laboratory of Molecular Cell Biology, Institute of Botany and Microbiology, KU Leuven.; 2 Department of Molecular Microbiology, VIB, Kasteelpark Arenberg 31, B-3001 Leuven-Heverlee, Flanders, Belgium.; 3 Agrobiodiversity Research Area, International Center for Tropical Agriculture (CIAT), Cali, Colombia.

**Keywords:** Mutant screen, genomic mutagenesis, non-selectable trait, polygenic trait, QTL mapping, genetic basis, flavor production, ethyl acetate production

## Abstract

Isolation of mutants in populations of microorganisms has been a valuable tool in experimental genetics for decades. The main disadvantage, however, is the inability of isolating mutants in non-selectable polygenic traits. Most traits of organisms, however, are non-selectable and polygenic, including industrially important properties of microorganisms. The advent of powerful technologies for polygenic analysis of complex traits has allowed simultaneous identification of multiple causative mutations among many thousands of irrelevant mutations. We now show that this also applies to haploid strains of which the genome has been loaded with induced mutations so as to affect as many non-selectable, polygenic traits as possible. We have introduced about 900 mutations into single haploid yeast strains using multiple rounds of EMS mutagenesis, while maintaining the mating capacity required for genetic mapping. We screened the strains for defects in flavor production, an important non-selectable, polygenic trait in yeast alcoholic beverage production. A haploid strain with multiple induced mutations showing reduced ethyl acetate production in semi-anaerobic fermentation, was selected and the underlying quantitative trait loci (QTLs) were mapped using pooled-segregant whole-genome sequence analysis after crossing with an unrelated haploid strain. Reciprocal hemizygosity analysis and allele exchange identified *PMA1* and *CEM1* as causative mutant alleles and *TPS1* as a causative genetic background allele. The case of *CEM1* revealed that relevant mutations without observable effect in the haploid strain with multiple induced mutations (in this case due to defective mitochondria) can be identified by polygenic analysis as long as the mutations have an effect in part of the segregants (in this case those that regained fully functional mitochondria). Our results show that genomic saturation mutagenesis combined with complex trait polygenic analysis could be used successfully to identify causative alleles underlying many non-selectable, polygenic traits in small collections of haploid strains with multiple induced mutations.

## INTRODUCTION

Random mutagenesis has been a powerful technique in microbial genetics for decades and has aided in identifying structural and regulatory genes involved in many biochemical pathways and cellular processes 1,2,3. A major drawback of this methodology is that mutants can only be isolated in phenotypes that in one way or another can be selected under a specific condition, in which only the mutants of interest can multiply and not the wild type or any other mutant strains. In addition, mutant screens have focused for many years on mutants carrying single mutations causing a specific phenotype because there were no efficient methodologies to identify multiple mutant genes responsible for establishing a single polygenic trait. Also in programs for improvement of industrial yeast strains, random approaches, like population mutagenesis, have often been more successful than rational approaches targeting specific genes 4,5,6. This likely reflects the lack of insight in the regulation of many metabolic pathways and cellular processes, especially in industrial yeast strains. In addition, the genetic basis of many commercially important traits is often poorly understood in laboratory yeast strains and even more in industrial yeast strains. Most traits of yeast that have industrial importance are non-selectable and polygenic. As a result, it has been difficult to obtain mutants in commercially-important traits of yeast and to identify the genes involved using classical complementation approaches.

The advent of high-throughput genome sequencing methodologies has allowed simultaneous mapping of QTLs underlying a polygenic trait-of-interest, while reciprocal hemizygosity analysis and allele exchange have allowed to identify the causative genes in the QTLs 7,8. This has now been performed with selectable or easily identifiable traits, allowing very high numbers of segregants to be used 9,10, but also with small pools of selected segregants for difficult-to-score commercially important traits 11,12,13,14,15,16,17,18,19,20,21,22,23,24,25,26. These new methodologies have allowed exploration of the huge *Saccharomyces cerevisiae* biodiversity to identify genes affecting commercially-important traits. This approach is limited, however, to genes sufficiently affected by natural variation to exert a significant effect on a trait-of-interest. There is no evidence that identification of all genes responsible for the natural variation in a trait-of-interest will reveal the complete genetic basis of this trait.

For non-selectable traits, on the other hand, all segregants need to be phenotyped individually, for instance in small scale fermentations, which can be quite laborious. There is however a tremendous interest in these non-selectable phenotypes 19,26,27,28 and recently Hubmann *et al*. 17 and Pais *et al*. 12 showed that even with a limited number of segregants causative alleles could be identified for maximal ethanol accumulation and low glycerol production. For similar reasons, also random mutagenesis techniques have been limited to selectable or at least easily identifiable phenotypes. The possibility to simultaneously identify several causative alleles, in the midst of many irrelevant mutations, has provided the opportunity to also expand mutagenesis treatments to polygenic non-selectable phenotypes.

A non-selectable trait that attracted much attention is the production of volatile esters because these esters are responsible for the highly desired fruity aroma character of alcoholic beverages, like beer, wine and sake 29,30,31,32,33. Although a number of structural genes involved in ester biosynthesis have been identified 34,35,36,37,38,39,40,41, little is known about the regulation of the biosynthetic pathways and about other systems influencing the production of specific flavor esters positively or negatively. Ethyl acetate is such a flavor-active ester, which has a desirable, fruity flavor in low concentrations, but an undesirable solvent-like flavor in higher concentrations. Atf1 and Atf2 have been shown to catalyze the synthesis of ethyl acetate from its two substrates, ethanol and acetyl-CoA, but an *atf1*Δ *atf2*Δ strain still produces considerable amounts of ethyl acetate 38. The genetic basis of the remaining ethyl acetate production remains unknown and provides an interesting non-selectable, likely polygenic phenotype to investigate with the genomic saturation mutagenesis technology. Identification of novel genes causing modified ethyl acetate production would allow for targeted strain improvement by genetic engineering but also provide new genetic targets for marker-assisted breeding 42, in order to refine the flavor profile of alcoholic beverages produced by yeast.

The polygenic nature of most traits requires many mutations to be introduced to create an appropriate base for their genetic analysis and an adaptation of current mutagenesis protocols is therefore necessary. Ultraviolet light (UV) and ethyl methanesulphonate (EMS) mutagenesis have been the most popular classical mutagenesis techniques in yeast but are limited in the number of mutations they introduce. Recently, Shiwa *et al*. 43 proposed an alternative mutagenesis technique using a mutant error-prone DNA polymerase δ. Their study showed, however, that, although creating a more diverse mutation profile, classical mutagenesis techniques were still superior in introducing multiple genomic mutations as assessed by whole genome sequencing. Therefore adaptation of existing UV or EMS protocols was considered to be a more appropriate approach to maximize mutation load.

In this work we have evaluated the two most commonly used mutagen treatments, UV and EMS, for maximal introduction of random mutations in a single haploid yeast strain. Using an optimized protocol, we show that these haploid strains can accumulate ~900 mutations while retaining genetic proficiency, i.e. maintenance of mating capacity, sporulation capacity of the diploid strain and spore viability of the segregants. The mutant strains can thus be used for QTL mapping of polygenic, non-selectable traits. As a proof-of-principle we have applied pooled-segregant whole-genome sequence analysis to identify QTLs for low ethyl acetate production. Further analysis of the QTLs with the strongest linkage has identified causative mutations in *PMA1*, and *CEM1* as well as *TPS1* as a causative genetic background allele. These results show that genomic saturation mutagenesis combined with polygenic analysis can be used successfully to identify the genetic basis of non-selectable polygenic traits.

## RESULTS

### Comparison of the efficiency of UV and EMS mutagenesis in enhancing mutation load in the absence of phenotypic selection.

**Figure 1:**
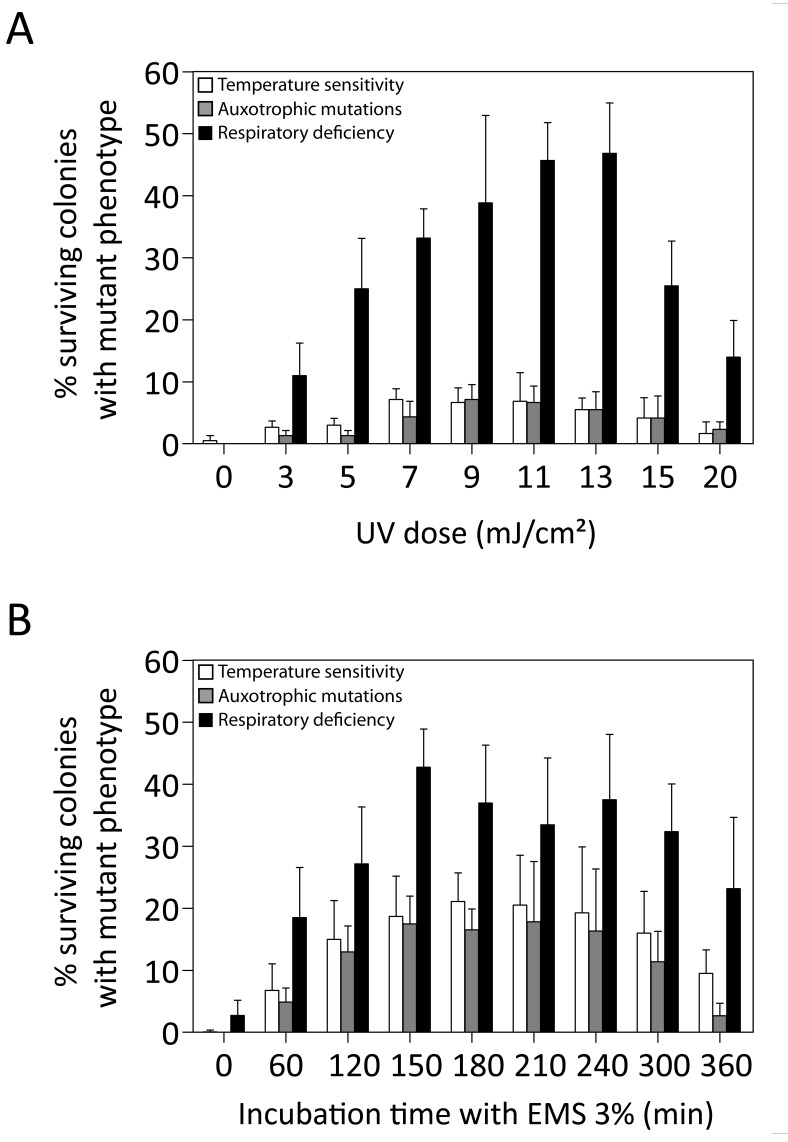
FIGURE 1: Mutation frequencies induced by different doses in UV and EMS mutagenesis. The S288c haploid yeast strain was submitted to mutagenesis by different doses of UV light **(A) **or different time periods of incubation with EMS **(B)**. The mutation frequency in surviving colonies was assessed through the presence of different types of mutations: temperature sensitivity (white bars), auxotrophic mutations on eight drop-out media (grey bars) and respiratory deficiency (black bars). No viable colonies were recovered after 420 min of incubation with EMS.

We first evaluated the efficiency of UV and EMS mutagenesis to cause accumulation of multiple mutations in single haploid yeast strains without selection for any phenotype. Haploid S288c *S. cerevisae* cells were irradiated with UV light at different energy levels or treated with 3% EMS for different time intervals and subsequently allowed to grow to single colonies on rich YPD medium. Mutagenesis efficiency was first estimated by assessing the presence of auxotrophic mutations on eight drop-out media (AUX), temperature sensitivity at 37°C (ts+) and respiratory deficiency (RD). For both mutagen treatments an optimum curve was obtained with increasing dose for the three types of mutations (Fig. 1). For the AUX and ts+ mutations, the maximum efficiency of EMS (Fig. 1B) was nearly three times higher than that of UV (Fig. 1A), with maxima of about 20% for EMS and about 7% for UV. The frequency of RD mutations was much higher than that of AUX or ts+ mutations, with maxima of 46.8% (at 13 mJ/cm^2^) and 42.77% (at 150 min) for UV and EMS, respectively. Mating of 160 RD mutants with BY4742 *ρ0* showed that 73% of RD mutants were caused by mitochondrial mutations. EMS was chosen as the preferred mutagen to maximize mutation load without selection and AUX and ts+ mutations were considered the most reliable measure for accumulation of genomic mutations. Although RD does not appear to be a good measure for genomic mutation load, it still appears to be a valuable read-out for the mutagenesis process.

### Accumulation of multiple mutations in single haploid strains by consecutive rounds of EMS mutagenesis.

**Figure 2:**
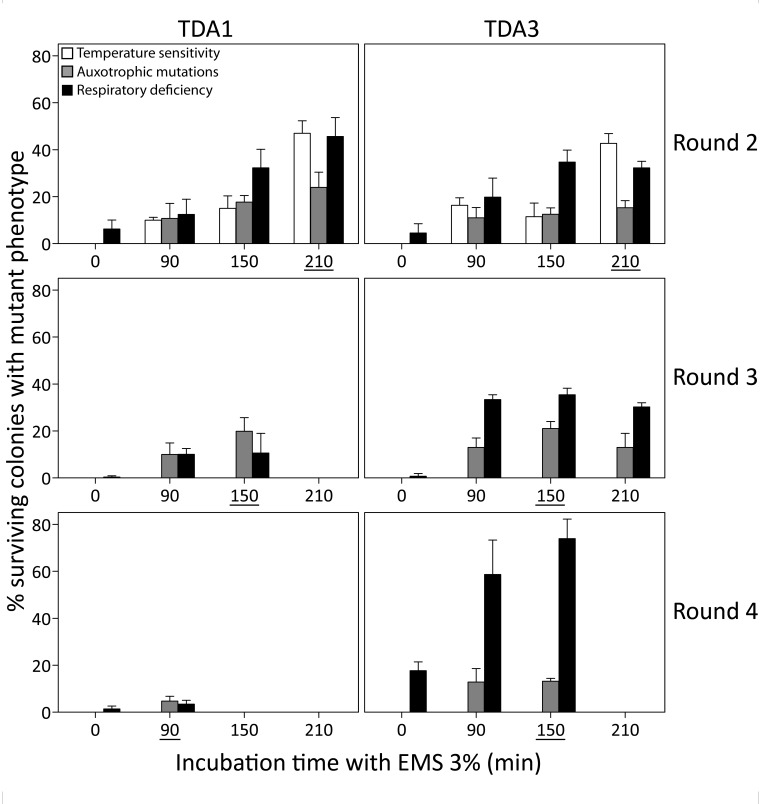
FIGURE 2: Mutation frequency after multiple EMS mutagenesis rounds. Two mutants, TDA1 and TDA3, selected after 210 min of EMS mutagenesis of the S288c strain, were submitted to three additional EMS mutagenesis rounds. In each round, the mutation frequency was assessed after 0, 90, 150, 210 and 360 min of incubation with EMS. It was based on temperature sensitivity (white bars), auxotrophic mutations on eight drop-out media (grey bars) and respiratory deficiency (black bars). The time period of incubation with EMS for each selected mutant is underlined in each mutagenesis round. For both mutants, no viable colonies were obtained after 360 min of EMS treatment in the second mutagenesis round and at the other time points at which no bars are present. Both mutant lines became temperature sensitive in round 2 and TDA3 acquired auxotrophic mutations for methionine and histidine in round 3.

Since single treatments with EMS result in a limited number of induced mutations 43,44,45,46, we performed multiple rounds of EMS mutagenesis to increase the mutation frequency further. Two mutants, TDA1 and TDA3, were selected after the first round of 210 min EMS mutagenesis of the haploid S288c strain based on maintenance of respiration, mating capacity (with S288c *MATα ura3::KanMX*), sporulation and a spore viability greater than 70%. For double selection in mating assays, the mutants were also selected to be prototrophic for uracil. The two mutants were each submitted to three additional rounds of EMS mutagenesis. In each round a single mutant was selected for the next mutagenesis round, based on the same criteria as applied after the first mutagenesis round (Fig. 2). TDA1-derived mutants were selected after 210, 150 and 90 min of EMS treatment in round 2, 3 and 4, respectively. In each of the four rounds, the selected TDA1-derived mutant lacked auxotrophic mutations and the same number of genes was thus tested in every round for the occurrence of mutations. TDA3-derived mutants were selected after 210, 150 and 150 min of EMS treatment in round 2, 3 and 4, respectively. In round 3, the TDA3-derived mutant selected had auxotrophic mutations for methionine and histidine. Hence, these two auxotrophies could no longer be tested in round 4 and the total number of genes evaluated for mutations in this round was thus reduced from 80 to 50. Maximum mutagenesis efficiencies were in all rounds within the range of that initially observed in S288c for both ts+ and AUX mutations except for round 4. A severe drop in efficiency was observed for TDA1 (AUX 4.7% at 90 min possibly due to the accumulation of synthetically lethal mutations. Both mutant lines became ts+ after the second mutagenesis round.

Whole genome sequence analysis of a single colony from each line, i.e. TDA1(4) and TDA3(4), was performed after four rounds of mutagenesis. Genomic DNA of both mutants and the original S288c strain was extracted and subjected to whole-genome sequence analysis using Illumina HiSeq2000 technology (BGI, Hong Kong, China). The sequence reads were mapped to the S288c reference sequence and variants were identified and filtered using the NGSEP pipeline 47. TDA1(4) and TDA3(4) had accumulated respectively 815 and 967 single nucleotide polymorphisms (SNPs) and small indels compared to the original S288c strain. The genomic loci of the 1294 variants located in ORFs were assessed for natural variation with ‘Align Strain Sequences’ at SGD (http://www.yeastgenome.org/cgi-bin/FUNGI/alignement.pl). The ORF sequences of an average of 40 yeast strains were compared to the S288c reference strain (Supplementary Tables S1 and S2). The 40 yeast strains were a selection out of 48 possible strains for which the specific ORF sequence was available, i.e. AWRI1631, AWRI796, BC187, BY4741, BY4742, CBS7960, CEN.PK, CLIB215, CLIB324, D273-10B, DBVPG6044, EC1118, EC9-8, FL100, FY1679, FosterB, FosterO, JAY291, JK9-3d, K11, Kyokai7, L1528, LalvinQA23, M22, PW5, RedStar, RM11-1a, SEY6210, SK1, Sigma1278b, T7, T73, UC5, UWOPS05_217_3, VL3, Vin13, W303, X2180-1A, Y10, Y55, YJM269, YJM339, YJM789, YPH499, YPS128, YPS163, YS9, ZTW1. More than 93% of the assessed genomic loci did not display any natural variation. Hence, the induced mutations in TDA1(4) and TDA3(4) in these loci were all unique compared to the sequence present in the natural yeast strains evaluated and thus likely absent from all or most of the biodiversity of *S. cerevisiae*. The majority of the SNPs present in the 7% genomic loci with sequence variation were also different from SNPs present in the natural alleles. Hence, genomic mutagenesis induces mutations that are generally different from those present in the natural biodiversity.

### Screening of TDA1(4) and TDA3(4) for non-selectable, polygenic phenotypes.

**Figure 3:**
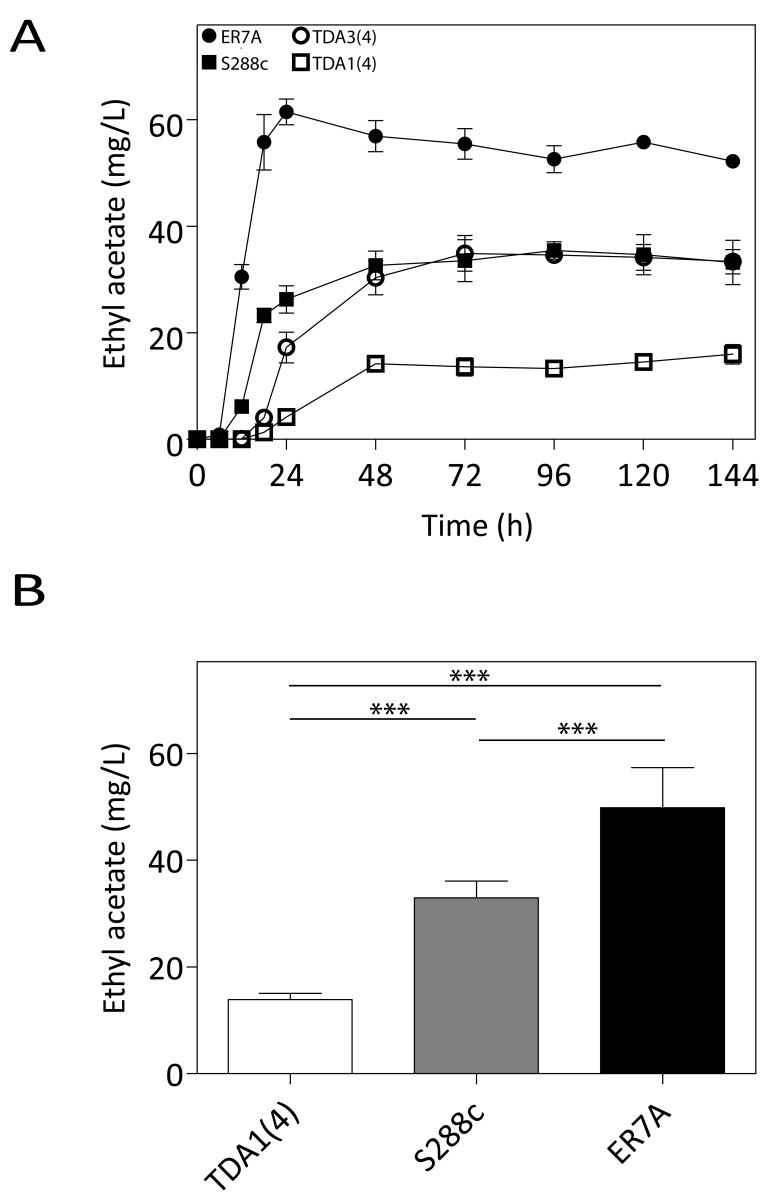
FIGURE 3: Ethyl acetate production in small-scale semi-anaerobic fermentations. **(A)** Ethyl acetate concentration during the course of a semi-anaerobic fermentation in 100 mL 10% YPD. Strains: (filled square) S288c, (filled circle) ER7A, (empty square) TDA1(4) and (empty circle) TDA3(4). **(B)** Ethyl acetate concentrations produced after 96 h, were analysed by a one-way ANOVA for TDA1(4) (white bar), S288c (grey bar) and ER7A (black bar) (p-value ≤0.001). Subsequent multiple comparison was carried out by a post hoc Tukey test with p-value ≤0.001 (***).

The two mutants, TDA1(4) and TDA3(4), were screened for flavor compound production as a typical example of a non-selectable and likely polygenic phenotype. This was performed in small scale (100 mL) semi-anaerobic fermentations with S288c and ER7A 17 as control strains. Fermentations were carried out in 10% YPD at 30°C with stirring at 80 rpm and a water lock placed on top. At the indicated times, samples were taken and flavor ester concentrations determined by headspace GC-FID. Fermentations finished in less than 96 h for all strains. Ethyl acetate production by TDA1(4) was considerably reduced at each time point compared to the control strains, while for TDA3(4) there was no significant difference in ethyl acetate production, although a slight delay in the onset was observed (Fig. 3A). We have subsequently chosen one fixed time point, i.e. 96 h, for determination of ethyl acetate levels in phenotyping the segregants for QTL mapping. After 96 h of fermentation, ethyl acetate concentrations were 13.9 ± 1.2 mg/L and 32.9 ± 3.2 mg/L for TDA1(4) and S288c, respectively (Fig. 3B). The low ethyl acetate production phenotype was chosen for further analysis as an example of a non-selectable, likely polygenic trait. For QTL mapping, ER7A (49.8 ± 7.5 mg/L) was chosen as inferior parent, instead of S288c, in order to have enough SNPs as genetic markers and because of the larger phenotypic difference in this case between the parent strains (Fig. 3B).

### Selection of superior segregants with low ethyl acetate production and QTL mapping by pooled-segregant whole-genome sequence analysis.

**Figure 4:**
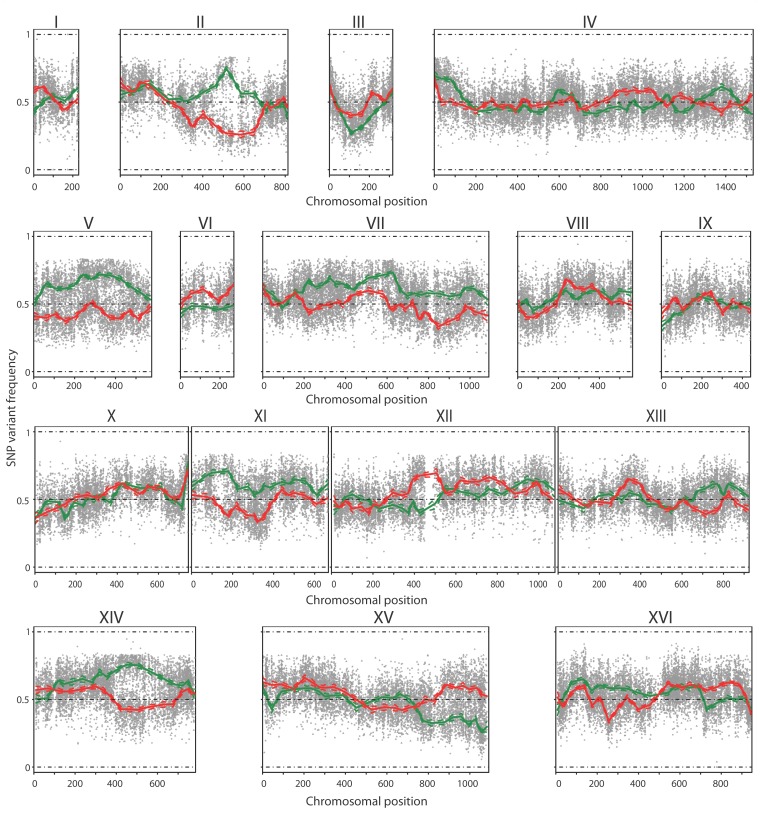
FIGURE 4: QTL mapping by pooled-segregant whole-genome sequence analysis of low ethyl acetate production. The SNP variant frequency was plotted versus the SNP chromosomal position for the 16 yeast chromosomes (raw data: grey circles for selected pool and grey triangles for random pool; smoothened data: solid green line for selected pool and solid red line for random pool; statistical confidence interval: dashed green and red lines). Significant upward deviation from 50% (>70%), indicating linkage to the genome of TDA1(4), was observed on chromosome II, V, VII, XI and XIV for the selected pool. Significant downward deviation from 50% (<30%), indicating linkage to the genome of ER7A, was observed on chromosome III and XV.

The TDA1(4)/ER7A hybrid diploid strain was constructed, sporulated and 386 segregants were isolated and characterized for ethyl acetate production after 96 h in small scale semi-anaerobic fermentations (Supplementary Table S3). Among these, 41 segregants showed a low ethyl acetate production (<20 mg/L) and were selected for QTL mapping with pooled-segregant whole-genome sequence analysis (Supplementary Fig. S1). As a control, 41 randomly selected segregants were pooled and also subjected to pooled-segregant whole-genome sequence analysis. Genomic DNA of the selected and random pools was extracted and sequenced as described for the mutant strains. The sequence reads were aligned with the S288c reference genome and quality-filtered SNPs were used as genetic markers, essentially as described previously 18. The ER7A parent had been sequenced previously 17. The SNP variant frequency of the two pools was then plotted against the SNP position throughout the genome (Fig. 4). Smoothening of the SNP variant frequency in both pools, under the form of smoothing splines, was accomplished using a linear mixed model framework 18. Seven regions showed a significant deviation from 50% (i.e. <30% or >70%). Two of these regions were located on chromosome III and XV and showed 27.2 and 25.9% linkage to ER7A, the inferior parent, respectively. These two QTLs were not further analysed since they were unlikely due to linkage with SNPs induced by mutagenesis in the non-selectable trait of low ethyl acetate production, which was the aim of this study. The other five regions were located on chromosome II, V, VII, XI and XIV and showed 74.4, 71.9, 73.2, 70.8 and 75.7% linkage, respectively, to the genome of TDA1(4), the superior parent.

### Fine-mapping of QTLs by allele specific PCR in individual segregants.

**Figure 5:**
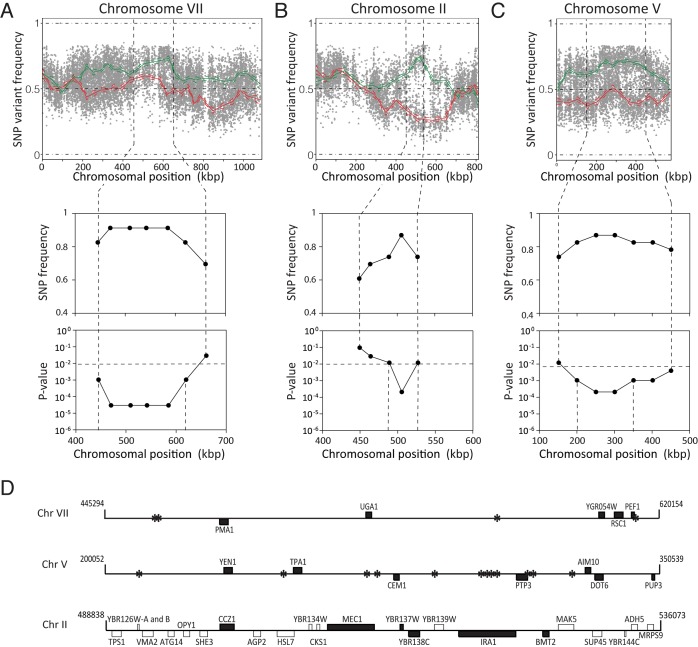
FIGURE 5: Fine mapping of QTLs on chromosome VII, II and V. Top: SNP variant frequency plotted versus SNP chromosomal position for chromosomes VII **(A)**, II **(B)** and V **(C)** (raw data: grey circles for selected pool and grey triangles for random pool; smoothened data: solid green line for selected pool and solid red line for random pool; statistical confidence interval: dashed green and red lines). Middle and bottom: SNP frequency and associated p-value as determined by allele specific PCR in the 23 selected individual segregants (<16.3 mg/L ethyl acetate), in the indicated regions on chromosome VII **(A)**, II **(B)** and V **(C)**. The statistical confidence line (p-value ≤0.01) is also indicated. **(D)** Overview of intergenic regions (*) and genes present on chromosomes VII, II and V with a mutation in TDA1(4) (black bars) compared to S288c. For chromosome II the genes with a mutation in ER7A (white bars) compared to S288c are also indicated

Fine-mapping of the five QTLs was performed by scoring selected individual SNPs by allele specific PCR in individual segregants. A more stringent cut-off was used for low ethyl acetate production in the individual segregants since in a separate S288c/ER7A cross, one out of 44 segregants accumulated 19.5 mg/L ethyl acetate, which is lower than the 20 mg/L cut-off used for QTL mapping. We chose 16.3 mg/L as the new cut-off since it was the highest measured ethyl acetate concentration in TDA1(4) after 96 h of fermentation. As a result, 18 TDA1/ER7A segregants were excluded from the fine-mapping. When the SNP variant frequency was determined in each QTL with the 23 remaining segregants of the selected pool, the strongest link to low ethyl acetate production was located in the QTL on chromosome VII, with a p-value of 3.02 x 10^-5^ (Fig. 5A). The two QTLs on chromosome II and V also showed strong linkage with both p-values being 2.11 x 10^-4^ (Fig. 5B, C). The two putative QTLs on chromosome XI and XIV were not significant with this number of segregants and at this stringency level since their p-values were above the 0.01 threshold (both 0.012). Further analysis was thus focused on the QTLs on chromosome VII, II and V.

### Identification of a causative allele in the QTL on chromosome VII.

**Figure 6:**
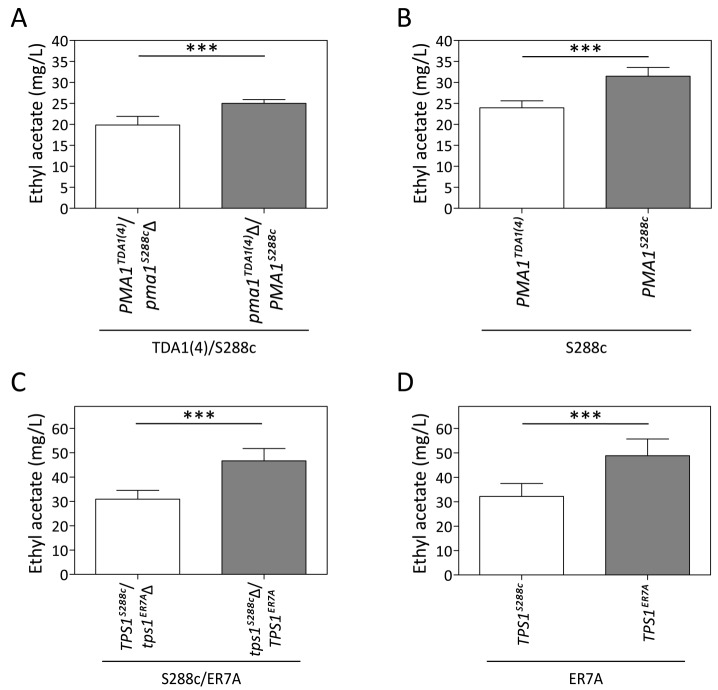
FIGURE 6. Reciprocal hemizygosity analysis (RHA) and allele exchange of the *PMA1* and *TPS1* alleles. Ethyl acetate production in semi-anaerobic fermentations after 96 h. **(A)** RHA of *PMA1* in the TDA1(4)/S288c hybrid strain. **(B)** Allele replacement of *PMA1*^S288c^ by *PMA1*^TDA1(4)^ in the S288c strain. **(C)** RHA of *TPS1* in the ER7A/S288c hybrid strain. **(D)** Allele replacement of *TPS1*^ER7A^ by *TPS1*^S288c^ in the ER7A strain. The results were analysed with an unpaired, two-sided Student t-test with p-value ≤0.001 (***).

Sequence analysis of the 175 kb region with strongest linkage on chromosome VII revealed seven intragenic and four intergenic SNPs in the TDA1(4) sequence compared to the sequence of the S288c parent strain (Fig. 5D). Except for one intergenic SNP located in an autonomously replicating sequence, these were evaluated as possible causative genetic elements by reciprocal hemizygosity analysis (RHA) 21. For each of the candidate causative variants, we constructed two hybrid hemizygous TDA1(4)/S288c strains differing only in the candidate locus, either by deleting the precise mutant ORF or the two neighboring ORFs for intra- and intergenic SNPs, respectively. Comparison of ethyl acetate production by the two hybrid strains for each of the eleven variants revealed a significant difference only for *PMA1* (Fig 6A and supplementary Fig S2). The hybrid with the *PMA1*^TDA1(4)^ allele showed a significantly reduced ethyl acetate production (19.8 ± 2.1 mg/L) compared to the hybrid with the *PMA1*^S288c^ allele (25.0 ± 0.9 mg/L). We further confirmed *PMA1* as causative allele for low ethyl acetate production by allele replacement in the S288c strain (Fig 6B). A significant reduction in ethyl acetate production was observed when *PMA1*^S288c^ was replaced by *PMA1*^TDA1(4)^ in the S288c strain (24.0 ± 1.7 mg/L compared to 31.5 ± 2.1 mg/L). Sequence comparison of the *PMA1*^TDA1(4)^ and *PMA1*^S288c^ alleles revealed one SNP in the ORF (position 482216 bp of chromosome VII), resulting in an M152I change in the amino acid sequence.

### Identification of the causative alleles in the QTLs on chromosome II and V.

Similar to the QTL on chromosome VII, RHA was performed for six and sixteen genes with a mutation in the QTLs on chromosome II and V, respectively (two intergenic SNPs were excluded on chromosome V because of their location between a TY element and a tRNA or an autonomously replicating sequence) (Fig. 5D). For none of the candidate mutant genes on chromosome II did we observe an allele-specific difference in ethyl acetate production (Supplementary Fig S3). This could be due to the fact that the causative gene in this QTL is not one of the genes with an induced mutation in TDA1(4) but rather a background allele of the S288c parent strain, causing lower ethyl acetate production compared to the ER7A parent. Hence, we performed RHA with all 23 ORFs containing SNPs between S288c and ER7A in the ER7A/S288c hybrid strain (Supplementary Fig S4). Only the hybrid expressing the *TPS1*^S288c^ allele showed a significantly reduced ethyl acetate production (30.9 ± 3.6 mg/L) compared to the hybrid expressing the *TPS1*^ER7A^ allele (46.7 ± 5.1 mg/L) (Fig. 6C). We further confirmed the *TPS1*^S288c^ involvement in ethyl acetate reduction by allele replacement in the ER7A background (Fig. 6D). A significant reduction in ethyl acetate production was observed when *TPS1*^ER7A^ was replaced by *TPS1*^S288c^ in the ER7A strain (48.9 ± 6.8 mg/L compared to 32.2 ± 5.3 mg/L ethyl acetate). The *TPS1* allele of ER7A contains only one single and synonymous mutation compared to the S288c allele, located 1152 bp (V384V) from the start codon. *TPS1*^ER7A^, however, also contained SNPs upstream (-226, relative to the start codon) and downstream (+61 and +130, relative to the stop codon) of the ORF. This suggests that differences in the expression level of *TPS1*^ER7A^ might be responsible for the higher ethyl acetate production in ER7A.

**Figure 7:**
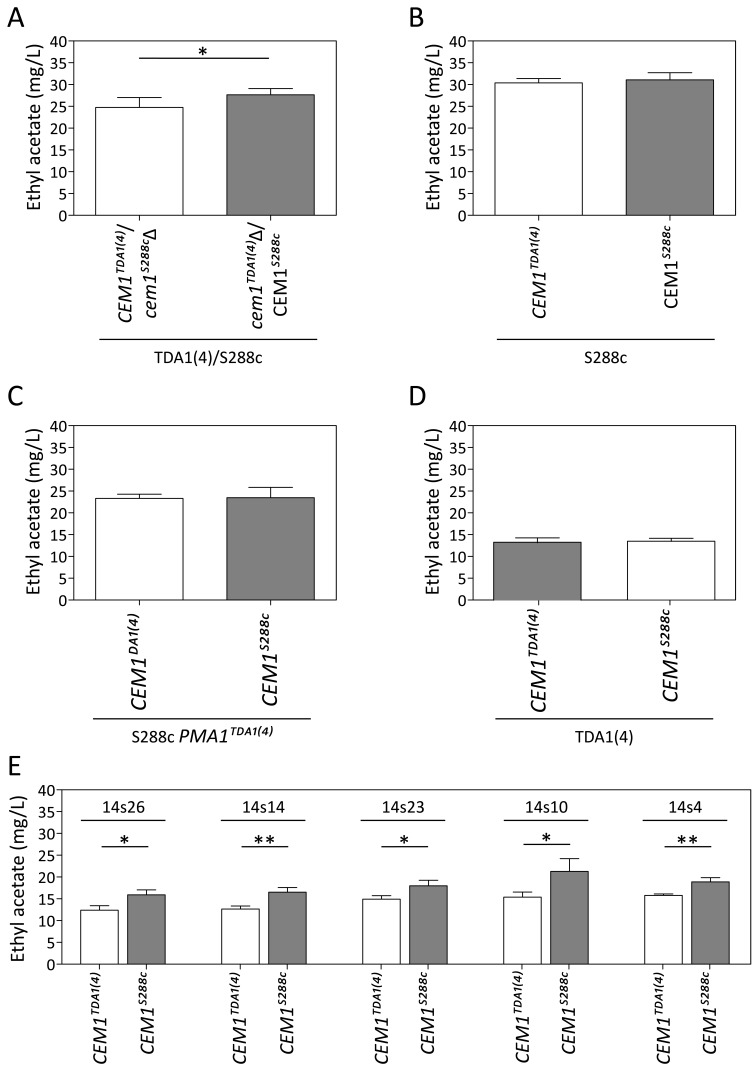
FIGURE 7: Reciprocal hemizygosity analysis (RHA) and allele exchange of the *CEM1* allele. Ethyl acetate production in semi-anaerobic fermentations after 96 h. **(A)** RHA of *CEM1* in the TDA1(4)/S288c hybrid strain. **(B)** Allele replacement of *CEM1*^S288c^ by *CEM1*^TDA1(4)^ in the S288c strain. **(C)** Allele replacement of both *CEM1*^S288c^ and *PMA1*^S288c^ by *CEM1*^TDA1(4) ^and *PMA1*^TDA1(4)^, respectively, in the S288c strain. **(D)** Allele replacement of *CEM1*^TDA1(4)^ by *CEM1*^S288c^ in the TDA1(4) strain. **(E)** Allele replacement of *CEM1*^TDA1(4)^ by *CEM1*^S288c^ in the five segregants of TDA1(4)/S288c with the lowest ethyl acetate production. The results were analysed with an unpaired, two-sided Student t-test with p-value ≤0.05 (*) or ≤0.01 (**).

For chromosome V, seven intragenic and nine intergenic SNPs, located in the most strongly linked 150 kb region, were analyzed for possible causative character (Fig. 5C). None of the TDA1(4)/S288c RHA strains constructed for these SNPs showed a significant difference in ethyl acetate production (Supplementary Fig. S5), except for *CEM1* (24.7 ± 2.3 mg/L compared to 27.7 ± 1.4 mg/L) (Fig. 7A). Sequence comparison of the *CEM1* allele from TDA1(4) with that of the S288c reference strain revealed one SNP in the *CEM1* ORF (position 278369 bp on chromosome V) resulting in an A420T amino acid change. Allele replacement of *CEM1* in the S288c strain, however, did not cause any significant change in ethyl acetate production (Fig 7B). We also constructed an S288c strain in which both *CEM1* and *PMA1* alleles were replaced with the TDA1(4) alleles, in order to investigate a possible dependence of the *CEM1*^TDA1(4)^ allele on the *PMA1*^TDA1(4)^ allele. Its ethyl acetate production was compared with that of the strain with only the *PMA1*^TDA1(4)^ allele replaced (Fig. 7C). However, again no difference in ethyl acetate production was observed. We further explored a possible epistatic interaction with another, yet unidentified, TDA1(4) allele. For this purpose, we replaced the *CEM1* allele in TDA1(4) by the S288c allele. Again no difference in ethyl acetate production could be observed between strains containing either *CEM1*^TDA1(4)^ or *CEM1*^S288c^ (Fig. 7D). The *CEM1* gene product is located in the mitochondria 48 and expression of the low ethyl acetate production phenotype might be dependent on proper mitochondrial function, which was lost after the 4^th^ round of mutagenesis in TDA1(4). When we assessed the presence of the *CEM1*^TDA1(4)^/*CEM1*^S288c^ and *PMA1*^TDA1(4)^/*PMA1*^S288c^ alleles in 44 segregants of the hybrid TDA1(4)/S288c, the five lowest ethyl acetate producing segregants all possessed both the *PMA1*^TDA1(4)^ and *CEM1*^TDA1(4)^ alleles (Table 1). All five segregants also had proper mitochondrial function as assessed by growth on YPSEG. This suggests that *PMA1*^TDA1(4)^ and *CEM1*^TDA1(4)^ are both indeed important for low ethyl acetate production and that the contribution of *CEM1*^TDA1(4)^ depends on mitochondrial function. Since these five segregants apparently contained most of the causative alleles for low ethyl acetate production present in TDA1(4), we replaced *CEM1*^TDA1(4)^ in these segregants by *CEM1*^S288c^. This resulted in a significant increase in ethyl acetate production in all five segregants (Fig. 7E). This result confirmed that the induced mutation in *CEM1* is causative for low ethyl acetate production and that it is dependent on proper mitochondrial function. Replacement of the *CEM1* allele in the S228c strain, which has proper mitochondrial function, did not cause an observable effect. Hence, it is likely that the induced mutation in *CEM1* is not only dependent on proper mitochondrial function but also on another, yet unidentified, causative induced mutation. This induced mutation is not present in S288c but is assumed to be present in all five TDA1(4)/S288c segregants with the lowest ethyl acetate production. In addition, these results showed that causative induced mutations can still be identified in the genetic analysis in spite of the fact that the mutation is unable to cause an observable phenotypic effect in the original strain with multiple induced mutations obtained by genomic saturation mutagenesis.

**Table 1:** Presence of the *PMA1* and *CEM1* alleles in 44 segregants of the TDA1(4)/S288c hybrid. The presence of the *CEM1^TDA1(4)^* or *CEM1^S288c^* and *PMA1^TDA1(4)^* or *PMA1^S288c^* alleles was assessed by Sanger sequencing in 44 segregants of the hybrid TDA1(4)/S288c strain. The level of ethyl acetate production in semi-anaerobic fermentations after 96 h is indicated for each segregant.

**Segregant**	***PMA1 *allele**	***CEM1 *allele**	**Ethyl acetate (mg/L)**
14s 26	TDA1(4)	TDA1(4)	11.6 ± 1.2
14s 14	TDA1(4)	TDA1(4)	12.9 ± 1.4
14s 23	TDA1(4)	TDA1(4)	13.8 ± 0.5
14s 10	TDA1(4)	TDA1(4)	13.9 ± 0.6
14s 4	TDA1(4)	TDA1(4)	14.9 ± 0.3
14s 3	TDA1(4)	S288c	15.7 ± 2.3
14s 32	TDA1(4)	S288c	15.9 ± 0.4
14s 37	TDA1(4)	TDA1(4)	16.0 ± 0.4
14s 39	S288c	S288c	16.2 ± 1.4
14s 5	S288c	TDA1(4)	17.5 ± 2.2
14s 8	TDA1(4)	TDA1(4)	17.6 ± 0.3
14s 21	TDA1(4)	S288c	18.0 ± 2.8
14s 18	TDA1(4)	S288c	18.4 ± 0.2
14s 33	TDA1(4)	S288c	19.0 ± 0.1
14s 19	S288c	TDA1(4)	19.2 ± 2.4
14s 31	TDA1(4)	TDA1(4)	19.4 ± 0.5
14s 35	S288c	TDA1(4)	19.5 ± 2.8
14s 15	TDA1(4)	S288c	19.5 ± 2.4
14s 44	TDA1(4)	S288c	19.7 ± 0.2
14s 27	S288c	S288c	19.9 ± 1.8
14s 6	S288c	S288c	20.0 ± 0.7
14s 7	TDA1(4)	S288c	21.0 ± 0.6
14s 42	TDA1(4)	S288c	21.0 ± 0.9
14s 9	S288c	S288c	21.2 ± 1.0
14s 24	S288c	TDA1(4)	21.4 ± 1.0
14s 1	S288c	TDA1(4)	22.4 ± 1.9
14s 25	TDA1(4)	S288c	22.8 ± 3.3
14s 43	S288c	TDA1(4)	23.0 ± 2.9
14s 30	S288c	TDA1(4)	23.3 ± 1.7
14s 34	TDA1(4)	TDA1(4)	23.6 ± 3.3
14s 17	TDA1(4)	S288c	23.8 ± 0.3
14s 11	S288c	TDA1(4)	24.1 ± 0.4
14s 12	TDA1(4)	S288c	25.4 ± 1.5
14s 40	TDA1(4)	TDA1(4)	25.7 ± 1.4
14s 28	S288c	TDA1(4)	27.9 ± 2.2
14s 2	S288c	S288c	28.2 ± 1.2
14s 41	S288c	TDA1(4)	28.2 ± 1.7
14s 38	S288c	S288c	30.0 ± 1.3
14s 13	S288c	S288c	30.5 ± 1.5
14s 16	S288c	TDA1(4)	30.5 ± 0.7
14s 29	S288c	S288c	30.8 ± 0.1
14s 22	S288c	S288c	32.3 ± 0.5
14s 20	S288c	TDA1(4)	34.1 ± 1.5
14s 36	S288c	S288c	37.0 ± 7.8

## DISCUSSION

The aim of this work was to explore whether the main disadvantage of classical mutant screens, i.e. the inability of isolating mutants in non-selectable, polygenic phenotypes, could be addressed by combining genomic saturation mutagenesis and QTL mapping based on pooled-segregant whole-genome sequence analysis. The first challenge was to accumulate enough mutations in the genome of a single haploid strain so as to create a significant chance that the multiply mutated strain will be affected in many non-selectable phenotypes while maintaining genetic proficiency. For that purpose, we optimized a methodology to accumulate a high number of mutations in a single haploid strain by applying multiple rounds of EMS mutagenesis. Intuitively, one would expect the strains with higher numbers of mutations to become affected in viability or vitality and therefore at some point strains with lower numbers of mutations to dominate in the surviving populations. Hence, genomic saturation mutagenesis would be limited by counterselection of the preferred multiply-mutated strains. In spite of this, we were able to obtain two haploid strains with about 900 mutations and given the steady increase in the number of mutations in the subsequent mutagenesis rounds we expect that considerably higher numbers of artificial mutations can be induced in a single genome, while still retaining the mating capacity required for genetic mapping. Moreover, most of the induced mutations were located at genomic loci not affected by natural variation, at least in the 48 strains for which genome sequence data are now available at SGD. At the genomic loci that were affected by natural variation, the induced mutations had low similarity with the nucleotide variation in these 48 strains as compared to the S288c sequence. These induced mutations can thus be used to unravel underlying genetic elements that would not be discovered by analyzing natural phenotypic diversity.

To estimate the number of mutations accumulated, we initially used the classical procedures based on phenotypic screening of auxotrophic, temperature sensitive and respiratory deficiency mutations. This is similar to the assays of mutation frequency in the literature, which are based on mutations causing a selectable phenotype in single genes 49,50. In our case, we selected single colonies from the whole population of surviving cells and tested each colony separately for the presence of auxotrophic, temperature sensitive and respiratory deficiency mutations. This had the advantage that many more genes could be assessed for the presence of mutations. Although we could use this approach to optimize the mutagenesis method and to demonstrate a steady increase in the number of mutations likely accumulated, we cannot exclude phenotypic bias in the number of mutations inferred. On the other hand, the strong drop in the cost of whole genome sequencing now allows to estimate the number of mutations accumulated directly and more reliably in multiple isolates after each mutagenesis round. Multiple rounds of EMS mutagenesis turned out to be much more efficient than multiple rounds of UV mutagenesis in inducing high numbers of mutations in single haploid strains when no direct selection for the mutants was applied.

The biggest challenge of the genomic saturation mutagenesis was the maintenance of genetic proficiency in the strains with multiple induced mutations. Since the goal of the new methodology is to use the multiply mutated strains for elucidating the genetic basis of non-selectable, polygenic traits by QTL mapping, the mutant haploid strains must maintain mating capacity with a haploid reference strain, sporulation capacity of the diploid strain and spore viability of the segregants. Strains that lost mating capacity could be eliminated quite easily by counterselection for a combination of a prototrophic marker and geneticin resistance. Sporulation capacity of the diploid was usually maintained. This is likely due to complementation by the partner genome of any recessive mutation that would compromise sporulation capacity. Only dominant mutations affecting sporulation capacity would present a problem but these are likely much more rare than recessive mutations. Surprisingly, the most challenging problem was the maintenance of spore viability in the segregants. A possible explanation is that the persistent mutagenesis causes accumulation of multiple lethal mutations in essential genes, which are suppressed by a lower number or even by only one suppresser mutation. For instance, in the cAMP-PKA pathway, a single mutation in *BCY1*, the gene encoding the regulatory subunit of protein kinase A, is able to suppress lethality caused by inactivation of *CYR1*, encoding adenylate cyclase, *RAS1/RAS2* or *CDC25/SDC25*, but also of lethal mutations in all these genes together 51. When this happens for multiple combinations of lethal and suppressor mutations, as soon as a spore inherits a single lethal mutation without its suppressor mutation, it will be dead. Hence, with an increasing number of mutations accumulated in further mutagenesis rounds, a single suppressor mutation present could suppress the lethal effect of an increasing number of lethal mutations introduced. When this happens with multiple suppressor mutations, it would rapidly compromise the possibility of isolating viable spores after crossing with another strain since two of the spores in a tetrad will always lack the suppressor mutation. In spite of this problem, we were able to maintain sufficient spore viability to allow genetic analysis with strains accumulating about 900 mutations. It is important to emphasize that mutations in essential genes are not necessarily completely inactivating mutations. They can also affect the expression level, the regulation or the cellular location of the gene product, and in this way cause specific phenotypes. Hence, we feel confident that the current number of about 900 mutations does not yet represent real saturation and that accumulation in a single strain of much higher numbers of useful mutations, i.e. mutations with a phenotypic effect, while maintaining genetic proficiency, is thus likely possible. This would create a strain collection useful for screening many non-selectable phenotypes, not only at the cellular but also at the subcellular or biochemical level, such as misregulation of cellular location or alteration of post-translational modification of specific proteins.

In the current study, we have screened the two strains with multiple induced mutations for deficiencies in flavor compound production, a non-selectable trait of crucial importance in the alcoholic beverage industry and of which the metabolic basis and regulation are only poorly understood 29,52,53. We found that the TDA1(4) multiply mutated strain had noteworthy low ethyl acetate production. The ease with which a deficiency in a target trait was identified shows the usefulness of this approach. It can be easily predicted that with higher numbers of mutations accumulated in single haploid strains, a small collection of multiply mutated haploid strains may be affected in a very high number of phenotypes that are all compatible with the requirements for genetic analysis. Abolishment or even a drastic change in the phenotype is not required. A statistically significant difference that is reliable enough to perform the subsequent genetic analysis is sufficient. Remarkably, it is not even required that the phenotype is observable in the multiply mutated strain. This is shown in the current study by the mutation in *CEM1* that requires proper mitochondrial function to have effect, while the parent multiply mutated strain no longer had proper mitochondrial function. Since proper mitochondrial activity was recovered in all of the viable segregants, the *CEM1* mutation could exert its effect in these segregants. This shows that the accumulation of useful mutations in the multiply mutated strain is not limited by the fact that these mutations can exert a recognizable phenotypic effect. The only requirement is that the mutation can exert its effect in a sufficient number of segregants. If this is the case, the locus of the mutation will be identified in the QTL mapping. In practice, efficient QTL mapping by pooled-segregant whole genome sequence analysis requires only about 30 selected segregants 12,18. 

In our work we have crossed the multiply mutated haploid strain TDA1(4) with an unrelated haploid strain, ER7A. This provides the advantage that thousands of SNPs are available for efficient QTL mapping. On the other hand, it also makes it possible that genetic background mutations rather than induced mutations are responsible for part of the difference in the phenotype. This was the case in our study for the *TPS1* gene. In principle, however, this does not have to be a disadvantage since it simply provides an additional way to discover gene products affecting the phenotype of interest. Since the genome sequence of the multiply mutated strain and its parent strain are known, these mutations can be identified easily.

In the present study, we have identified three new genes affecting ethyl acetate production. On chromosome VII we identified *PMA1*^TDA1(4)^ as causative allele for lowering ethyl acetate production. The mutation causes an M152I amino acid change in transmembrane domain II of Pma1, an essential H+-ATPase that maintains the electrochemical proton gradient over the plasma membrane. Transmembrane domain I and II are linked by a short intracellular loop and form a conformationally-sensitive structure. Many recessive mutations in transmembrane domain I or II are lethal or cause a reduction in H+-ATPase activity 54. An amino acid change in this region could thus have an important effect on H+-ATPase activity and in this way affect many traits, including ethyl acetate production. The precise molecular mechanism, however, remains unclear.

As previously mentioned, the *CEM1*^TDA1(4)^ causative allele presents an interesting case since it was identified as causative allele in the genetic analysis with the segregants, although its effect could not be observed in the original TDA1(4) strain. The *CEM1* gene product is a beta-keto-acyl synthase located in the mitochondria and required for mitochondrial respiration 48. This may explain why the effect of the *CEM1*^TDA1(4)^ allele on ethyl acetate production is dependent on proper mitochondrial function. The *CEM1* gene product has never been connected to ethyl acetate biosynthesis. It is a homolog of the *FAS2* gene product, the alpha subunit of fatty acid synthetase, which synthetizes aceto-acetyl(-ACP) in the fatty acid biosynthesis pathway. Since both fatty acid biosynthesis and ethyl acetate biosynthesis use acetyl-CoA as substrate, one possible explanation for the reduction of ethyl acetate production by the *CEM1*^TDA1(4)^ allele, is lowering of the acetyl-CoA level due to enhanced activity of the fatty acid biosynthesis pathway. This result indicates the potential of the novel methodology proposed in this paper to obtain new insight in the functioning and interaction between metabolic pathways. 

A number of mutant alleles identified with the current methodology will undoubtedly only very indirectly affect the trait-of-interest, but this also applies to classical mutant screens using selectable phenotypes. This situation may be true for the *PMA1* gene product although it may also reflect our lack of insight in the regulation of metabolism and the presence of additional unanticipated functions of well-established proteins. This may also be true for the *TPS1* gene product, of which the S288c allele contributed to the low ethyl acetate production in the TDA1(4) strain. *TPS1* encodes trehalose-6-phosphate synthase, the first enzyme of trehalose biosynthesis. Its product, trehalose-6-phosphate, is also involved in allosteric regulation of glycolysis at the level of hexokinase 55. Tps1 may thus affect the levels of ethanol and acetyl-CoA, the substrates for ethyl acetate biosynthesis. However, multiple results suggest additional regulatory functions for Tps1 56,57,58,59,60.

In conclusion, our work has shown that genomic saturation mutagenesis combined with complex trait polygenic analysis can be used successfully to identify causative alleles underlying non-selectable, polygenic traits. Small collections of independently mutagenized haploid strains, containing very high numbers of induced mutations, could be screened for any non-selectable phenotype, including structural phenotypes at the subcellular level and biochemical phenotypes at the protein or metabolite level. The genetic basis can then be identified by the now well-established methodology for polygenic analysis of complex traits.

## MATERIALS AND METHODS

### Yeast strains, growth conditions, mating and sporulation

All *S. cerevisiae* strains used are listed in Table 2. S288c *ura3::KanMX* was constructed as described 61 and homologous integration verified by polymerase chain reaction (PCR). BY4742 *ρ0* was obtained according to Fox *et al*. 62. Yeast strains were grown at 30°C and 200 rpm in YPD medium containing 1% yeast extract, 2% Bacto peptone and 2% glucose. Where appropriate the medium was supplemented with 200 µg/mL geneticin or 100 µg/mL nourseothricin. Other media used are SD [0.171% Yeast Nitrogen Base w/o amino acids (Difco), 0.5% ammonium sulphate (or 0.1% glutamate if supplemented with geneticin) and 2% glucose at pH 6.5], YPSEG (1% yeast extract, 2% Bacto peptone, 1% succinic acid, 2% glycerol and 2% ethanol at pH 5.5) and sporulation medium (0.5% potassium acetate at pH 6.0). 1.5% Bacto agar was used to make solid nutrient plates. All percentages indicate weight per volume, unless stated otherwise. Mating, sporulation and dissection of tetrads were carried out according to standard protocols 63.

**Table 2:** *S. cerevisiae *strains used in this study.

**Strain**	**Genotype/Description**	**Source**
S288c	*MAT****a*** prototroph	64
S288c *MATα*	Mating type switched S288c	This study
S288c 2n	Isogenic S288c diploid	This study
S288c *ura3*::*KANMX*	*MATα ura3*::*KANMX*	This study
BY4742 *ρ0*	*MATα his3∆1 leu2∆0 ura3∆0 lys2∆0 ρ0*	This study
ER7A	Segregant 7A of Ethanol Red, Matα	17
*ade2Δ*	(BY4741) *MAT****a**** his3∆1 leu2∆0 ura3∆0 met15∆0 ade2::KANMX*	65
*arg2Δ*	(BY4741) *MAT****a**** his3∆1 leu2∆0 ura3∆0 met15∆0 arg2::KANMX*	65
*his3Δ1*	(BY4741) *MAT****a**** his3∆1 leu2∆0 ura3∆0 met15∆0*	64
*Leu2Δ0*	(BY4741) *MAT****a**** his3∆1 leu2∆0 ura3∆0 met15∆0*	64
*lys2Δ0*	(BY4742) *MATα his3∆1 leu2∆0 ura3∆0 lys2∆0*	64
*met15Δ0*	(BY4741) *MAT****a**** his3∆1 leu2∆0 ura3∆0 met15∆0*	64
*ura3Δ0*	(BY4741) *MAT****a**** his3∆1 leu2∆0 ura3∆0 met15∆0*	64
*trp1Δ*	(BY4741) *MAT****a**** his3∆1 leu2∆0 ura3∆0 met15∆0 trp1::KANMX*	65

### Mutagenesis

Yeast cells were grown to stationary phase, harvested, washed twice and re-suspended for EMS mutagenesis in 1 mL 0.1 M sodium phosphate buffer (pH 7.0) at a concentration of 2 x 10^8^ cells/mL. The samples were incubated with a final concentration of 3% (v/v) EMS for different time periods at 30°C and 200 rpm. EMS was neutralized by washing twice with 5% sodium thiosulphate 2. The pelleted cells were re-suspended in 1 mL sterile water and spread on YPD plates at appropriate dilutions. For UV mutagenesis the samples were re-suspended in 1 mL sterile water at a concentration of 2 x 10^8^ cells/mL, spread on 120 mm x 120 mm YPD plates at appropriate dilutions and irradiated with UV light at different energy levels (GS Gene Linker, BIO-RAD) 66. Single colonies were manually re-plated on YPD plates and allowed to grow for 3 days. Auxotrophic mutations were determined by re-plating the colonies on eight drop-out media (SD-ADE, SD-ARG, SD-HIS, SD-LEU, SD-LYS, SD-MET, SD-TRP, SD-URA) at 30°C. To determine temperature sensitivity and respiratory deficiency the colonies were re-plated on YPD at 37°C and on YPSEG at 30°C, respectively. YPD and SD plates incubated at 30°C served as control. For EMS and UV mutagenesis of the S288c strain a total of respectively 1536 and 576 colonies were analyzed at each indicated time interval. For the strains with multiple induced mutations 288 colonies were analyzed. The same single colonies were allowed to mate with S288c *MATα ura3::KanMX* in liquid YPD, diploids selected on SD-URA plates supplemented with 200 µg/mL geneticin, sporulated for 7 days and 20 random tetrads dissected to assess spore viability.

### General molecular biology methods 

Yeast cells were transformed with the LiOAc/SS-DNA/PEG method 67,68. Yeast genomic DNA was extracted with Phenol/Chloroform/Isoamyl-alcohol (25:24:1) 69 and further purified with ethanol precipitation if required. PCR was performed with high-fidelity polymerases Phusion and Q5 (New England Biolabs) or ExTaq (Takara) and Taq (New England Biolabs) for diagnostic purposes. Sanger sequencing 70 was performed by the VIB Genetic Service Facility (Antwerp). The sequences were analyzed with Vector NTI (Invitrogen) or CLC DNA workbench (CLC Bio) software. Lists of primers and plasmids are provided as supplementary tables S4 and S5, respectively.

### Fermentation conditions

The cells were pre-grown in 3 mL YPD medium for 24 h and subsequently grown to stationary phase for 2 days in 40 mL YPD medium. Cells were washed with sterile, distilled water and used to inoculate 100 mL 10% YPD medium in 100 mL flasks. Fermentations were carried out at 30°C with stirring at 80 rpm with a magnetic rod (30 x 6 mm) and a water lock on top to create semi-anaerobic conditions. Samples for gas chromatographic analysis were prepared by centrifugation and immediate cooling (on ice) of the supernatant in an airtight container at indicated times.

### Headspace gas chromatography

Headspace gas chromatography coupled with flame ionization detection (GC-FID) was used for the measurement of ethyl acetate in the fermentation samples. Samples of 5 mL were collected in 15 mL pre-cooled glass tubes and analyzed with a calibrated Trace Ultra gas chromatograph (Thermo Scientific) equipped with a Stabilwax column (length 60 m, internal diameter 0.25 mm, layer thickness 0.25 µm; Restek) and coupled to a Triplus RSH headspace autosampler (Thermo Scientific). Samples were heated for 10 min at 60 °C, with agitation, in the headspace autosampler. The injection block and flame ionization detector were kept at constant temperatures of 220°C and 250°C, respectively, and helium was used as a carrier gas. The oven temperature was held at 50°C for 5 min, then increased to 240°C at a rate of 15°C per min. Results were analyzed with Chromquest software (Thermo Scientific).

### Whole genome sequence analysis of mutant strains

High molecular weight DNA (5 µg; ~20 kb fragments) was isolated according to Johnston and Aust 71. The purity of the DNA sample was estimated from UV measurement (260/280=1.7-2.0). The DNA samples were provided to BGI (Hong Kong, China) for whole genome sequence analysis by Illumina technology at ~60 fold nominal coverage. The NGSEP pipeline 47 version 2.1.3 was used to align reads to the reference genome S288c, and to identify and filter variants relative to S288c. In brief, reads were aligned to the reference genome using bowtie2 72 with minimum insert size 350, maximum insert size 550 and allowing up to 3 valid alignments per read. Then alignments were sorted and variants were identified by the FindVariants command of NGSEP using its recommended parameters for WGS data: 1) Maximum base quality score 30; 2) Minimum genotype quality 40; 3) At most two alignments starting at each genomic site were considered; 4) Sample ploidy equal to 1; 5) Prior heterozygosity rate 10E-6. Variants falling into repetitive regions or regions with predicted copy number variation were filtered out using the FilterVCF command of NGSEP.

### Pooled-segregant whole-genome sequence analysis

After crossing the two parent strains, TDA1(4) and ER7A, 41 superior segregants (ethyl acetate production <20 mg/L after 96 h of fermentation in two biological replicates) were used to assemble the ‘selected pool’. A ‘non-selected’, i.e. random pool of equal size was also assembled. All segregants were grown separately and the pools were made by combining equal amounts of cells based on OD_600_. Whole-genome sequence analysis was carried out in a similar way as for the mutant strains at ~40 fold nominal coverage. Sequence alignment was performed using SeqMan NGen 4 (DNASTAR) and assembly and mapping with Lasergene 10 (DNASTAR). A variant was called if it was covered by at least 25 sequence reads with a quality score of at least 10 and minimum 90% of the reads contained the variant. QTL analysis was carried out as described previously 18. Essentially, SNP variant frequencies were calculated by dividing the number of the variant by the total number of reads at each SNP location. Deviation from 50%, either above or below, was a sign of a one-sided SNP variant segregation, indicating a genetic linkage to the trait of interest.

### Detection of SNP markers by allele specific PCR in individual segregants

SNPs were scored by allele specific PCR in individual segregants of TDA1(4)/ER7A, that accumulated <16.8 mg/L ethyl acetate after 96 h fermentation. The forward and reverse primer each contained either the S288c or ER7A nucleotide as the 3’ terminal nucleotide and were spaced between 400 and 1700 bp apart. The annealing temperature was optimized using genomic DNA of both parents to allow only hybridization with primers containing the exact complement. The SNP data in the individual segregants were analyzed using the binomial exact test.

### Reciprocal hemizygosity analysis (RHA)

For RHA analysis 21 the exact ORF or the two neighboring ORFs for intragenic or intergenic variants, respectively, were deleted with *NatMX4* 61 in the TDA1(4)/S288c or S288c/ER7A hybrid diploid strains. Homologous integration was verified by PCR and the remaining, i.e. non-deleted, allele identified by Sanger sequencing. RHA analysis was performed with two independent isolates of all diploids tested and fermentations were repeated twice.

### Allele replacement

Reciprocal allele replacements at the *CEM1* locus in S288c and TDA1(4) and the *TPS1* locus in ER7A were achieved by a two step-method. In the first step, *CEM1* or *TPS1* was deleted with a deletion cassette amplified by PCR and containing both the selectable *NatMX4* and counter-selectable *GIN11* marker 17. After transformation, positive clones were selected on nourseothricin containing YPD plates. The presence of the deletion cassette was verified by PCR. In the second step, either the S288c or TDA1(4) allele for *CEM1* or the S288c allele for *TPS1* were amplified from genomic DNA and inserted at the deletion site. Positive clones were selected on galactose containing medium by inducing the counter-selectable *GIN11* marker 73,74. Integration was assessed by nourseothricin sensitivity and the inserted allele verified by PCR and Sanger sequencing. The allele replacement of the essential gene *PMA1* was done similarly to *CEM1* in an isogenic S288c diploid. The latter strain was generated by mating type switching 75 and a subsequent backcross to the original S288c *MATa* strain. In the S288c *MATa* strain the HO-gene was expressed from the pFL39 GAL1 HO *KanMX* plasmid to induce mating type switch. Mating type was determined by PCR for the *MAT* locus 76. The near-isogenic S288c *PMA1*/*PMA1*^TDA1(4)^ diploid was sporulated to generate S288c *MATa* *PMA1*^TDA1(4)^. Double allele replacement of both *CEM1* and *PMA1* in S288c *MATa* was accomplished by mating S288c *MATa* *CEM1*^TDA1(4)^ with S288c *MATα* *PMA1*^TDA1(4)^ and subsequent sporulation of the diploid. Correct presence of the alleles was verified by Sanger sequencing. For all allele replacements, except the double replacement, 2 independent clones were tested and fermentations were repeated twice.

### Data access

Sequencing data have been deposited at the SRA database (NCBI), http://www.ncbi.nlm.nih.gov/sra with the account number SRP044392.

## SUPPLEMENTAL MATERIAL

Click here for supplemental data file.

All supplemental data for this article are also available online at http://microbialcell.com/researcharticles/genomic-saturation-mutagenesis-and-polygenic-analysis-identify-novel-yeast-genes-affecting-ethyl-acetate-production-a-non-selectable-polygenic-trait/.
