# Supplementary material for: Genomic saturation mutagenesis and polygenic analysis identify novel yeast genes affecting ethyl acetate production, a non-selectable polygenic trait
Source: Microb Cell. 2016 Mar 18;3(4):159–75. doi: 10.15698/mic2016.04.491 (PMC5349090; doi:10.15698/mic2016.04.491)
Supplement: Supplementary file 1 [file mic-03-159-s01.pdf]

Supplementary Figure S1. Ethyl acetate production in 386 segregants of TDA1(4)/ER7A. Histogram of ethyl acetate concentration for all 386 S288c/ER7A segregants in semi-anaerobic fermentations after 96h. Black bars: segregants present in the selected pool for pooled-segregant whole-genome sequence analysis, based on the ability to ferment to dryness within 96 h and producing an ethyl acetate concentration lower than the 20 mg/L cut-off. White bars: segregants not present in the selected pool. Fermentations were performed only once except for the 41 segregants in the selected pool. For these segregants, fermentations were repeated twice and ethyl acetate concentration was consistently below 20 mg/L.

Supplementary Figure S2. Reciprocal hemizygosity analysis (RHA) of genes with an induced mutation in the QTL on chromosome VII. Ethyl acetate production in semi-anaerobic fermentations after 96h. RHA was performed for all genes containing an induced mutation in the strongest linked region on chromosome VII as determined by allele specific PCR. For RHA, a pair of diploid TDA1(4)/S288c strains were constructed with either the S288c allele or the TDA1(4) allele deleted.

Supplementary Figure S3. Reciprocal hemizygosity analysis (RHA) of genes with an induced mutations in the QTL on chromosome II. Ethyl acetate production in semi-anaerobic fermentations after 96h. RHA was performed for all genes containing an induced mutation in the strongest linked region on chromosome II as determined by allele specific PCR. For RHA, a pair of diploid TDA1(4)/S288c strains were constructed with either the S288c allele or the TDA1(4) allele deleted.

Supplementary Figure S4. Reciprocal hemizygosity analysis (RHA) of all genes present in the QTL on chromosome II. Ethyl acetate production in semi-anaerobic fermentations after 96h. RHA was performed for all genes containing a genetic difference between S288c and ER7A in the strongest linked region on chromosome II as determined by allele specific PCR. For RHA, a pair of diploid S288c/ER7A strains were constructed with either the ER7A allele or the S288c allele deleted.

Supplementary Figure S5. Reciprocal hemizygosity analysis (RHA) of genes with an induced mutation on chromosome V. Ethyl acetate production in semi-anaerobic fermentations after 96h. RHA was performed for all genes containing an induced mutation in the strongest linked region on chromosome V as determined by allele specific PCR. For RHA, a pair of diploid TDA1(4)/S288c strains were constructed with either the S288c allele or the TDA1(4) allele deleted.

Supplementary Figure S1.tif @ 25% (Gray/8#) *

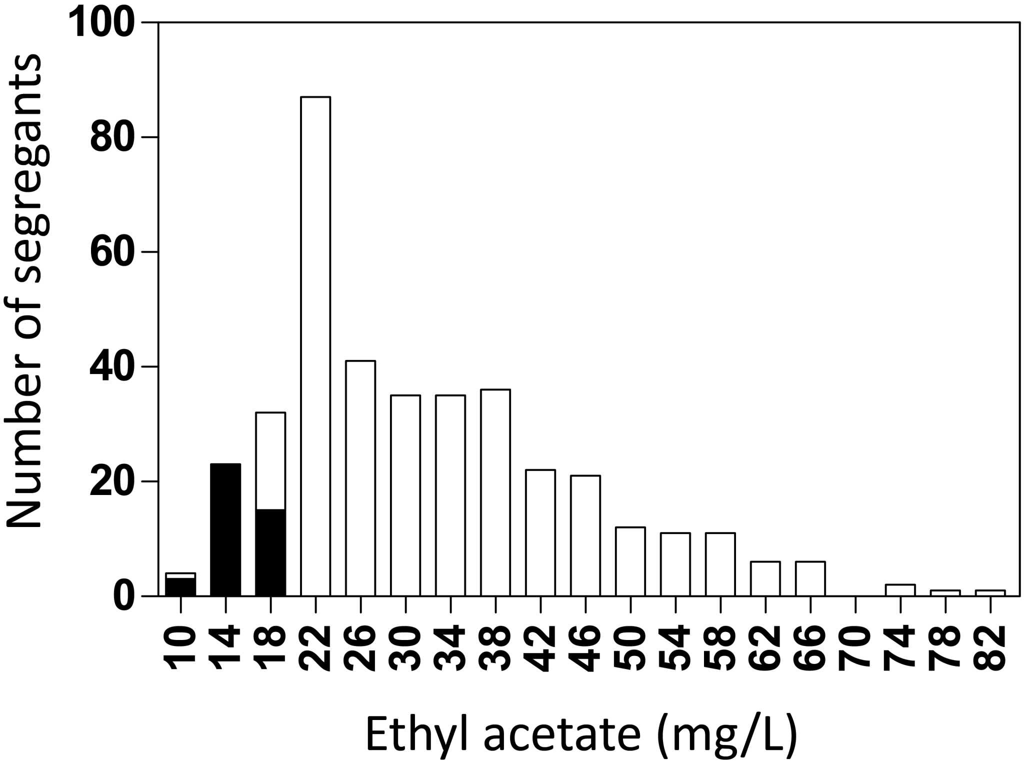

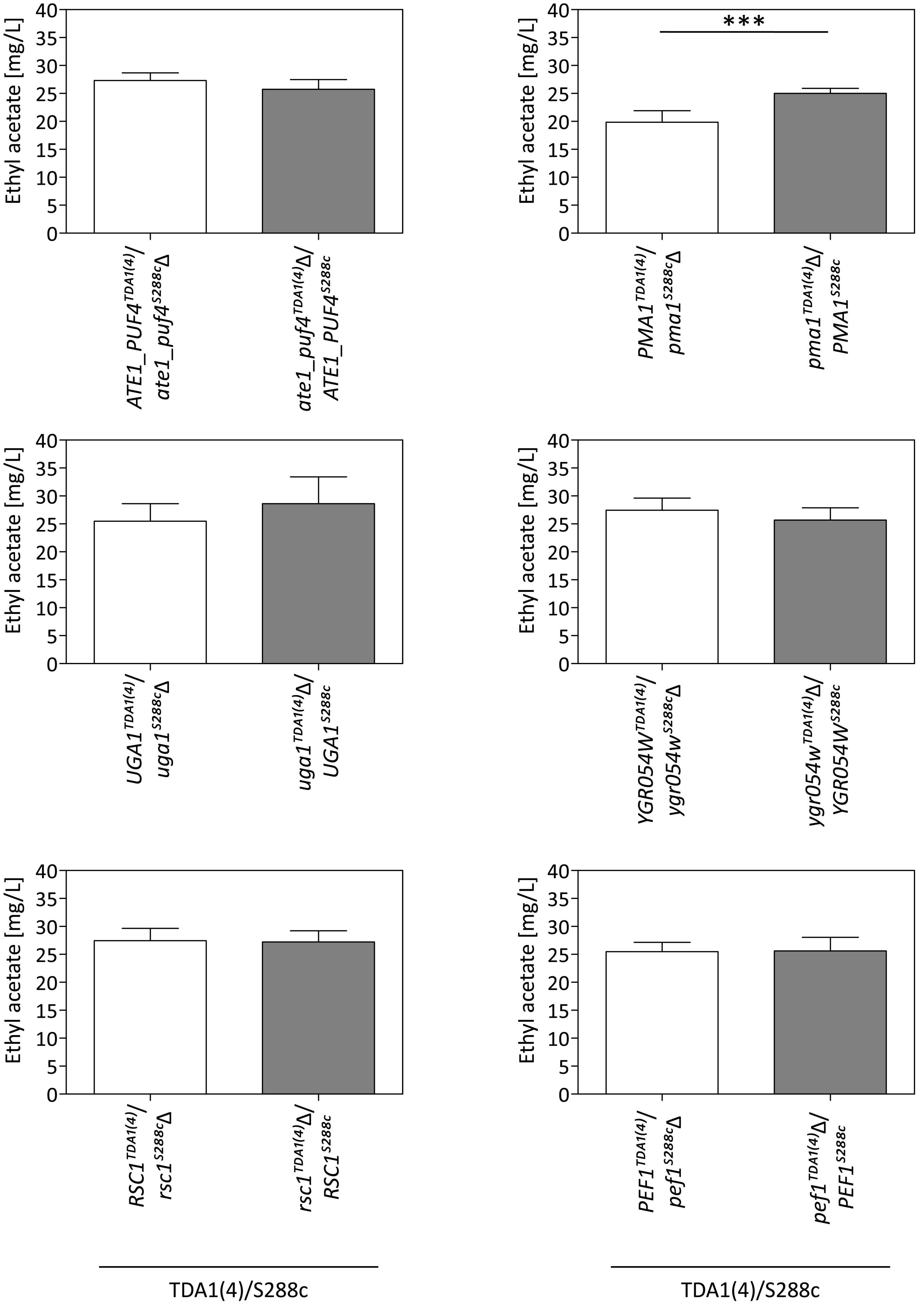

Ethyl acetate [mg/L]
0
5
10
15
20
25
30
35
40
***
ATE1_PUF4^TDA1(4)/ ate1_puf4^S288cΔ
ate1_puf4^TDA1(4)Δ/ ATE1_PUF4^S288c
PMA1^TDA1(4)/ pma1^S288cΔ
pma1^TDA1(4)Δ/ PMA1^S288c
UGA1^TDA1(4)/ uga1^S288cΔ
uga1^TDA1(4)Δ/ UGA1^S288c
YGR054W^TDA1(4)/ ygr054w^S288cΔ
ygr054w^TDA1(4)Δ/ YGR054W^S288c
RSC1^TDA1(4)/ rsc1^S288cΔ
rsc1^TDA1(4)Δ/ RSC1^S288c
PEF1^TDA1(4)/ pef1^S288cΔ
pef1^TDA1(4)Δ/ PEF1^S288c
TDA1(4)/S288c
TDA1(4)/S288c

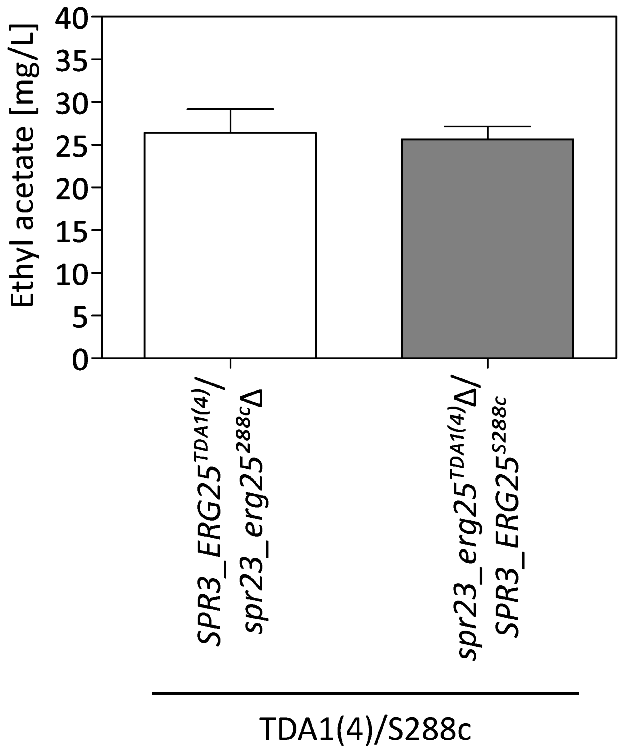
Ethyl acetate [mg/L]
40
35
30
25
20
15
10
5
0
SPR3_ERG25TDA1(4)/
spr23_erg25288cΔ
spr23_erg25TDA1(4)Δ/
SPR3_ERG25S288c
TDA1(4)/S288c

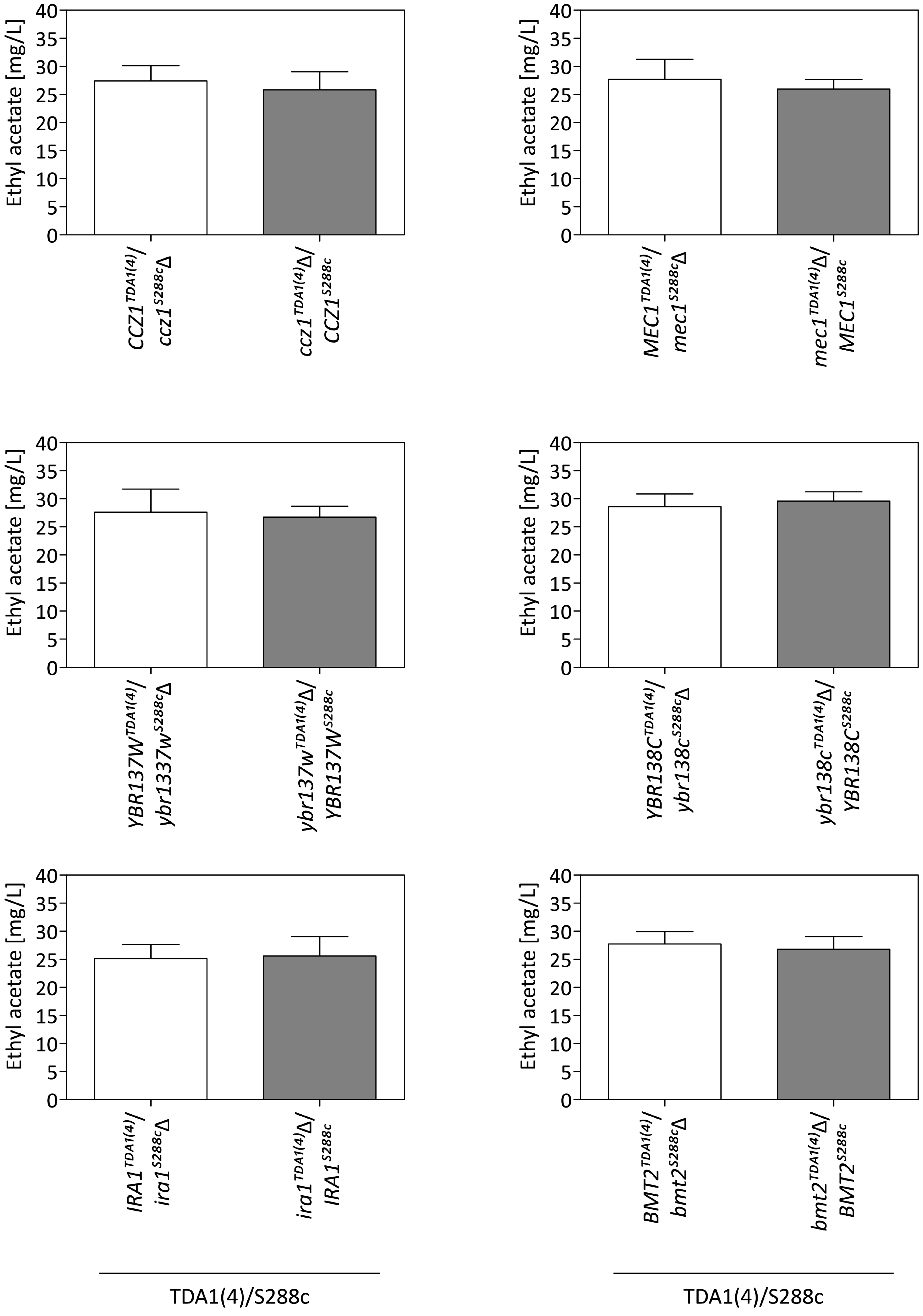
Ethyl acetate [mg/L]
0
5
10
15
20
25
30
35
40
CCZ1TDA1(4)/ ccz1S288cΔ
ccz1TDA1(4)Δ/ CCZ1S288c
MEC1TDA1(4)/ mec1S288cΔ
mec1TDA1(4)Δ/ MEC1S288c
YBR137WTDA1(4)/ ybr1337wS288cΔ
ybr137wTDA1(4)Δ/ YBR137WS288c
YBR138CTDA1(4)/ ybr138cS288cΔ
ybr138cTDA1(4)Δ/ YBR138CS288c
IRA1TDA1(4)/ ira1S288cΔ
ira1TDA1(4)Δ/ IRA1S288c
BMT2TDA1(4)/ bmt2S288cΔ
bmt2TDA1(4)Δ/ BMT2S288c
TDA1(4)/S288c
TDA1(4)/S288c

Supplementary Figure S4a.tif @ 8,33% (Gray/8#) [illegible]

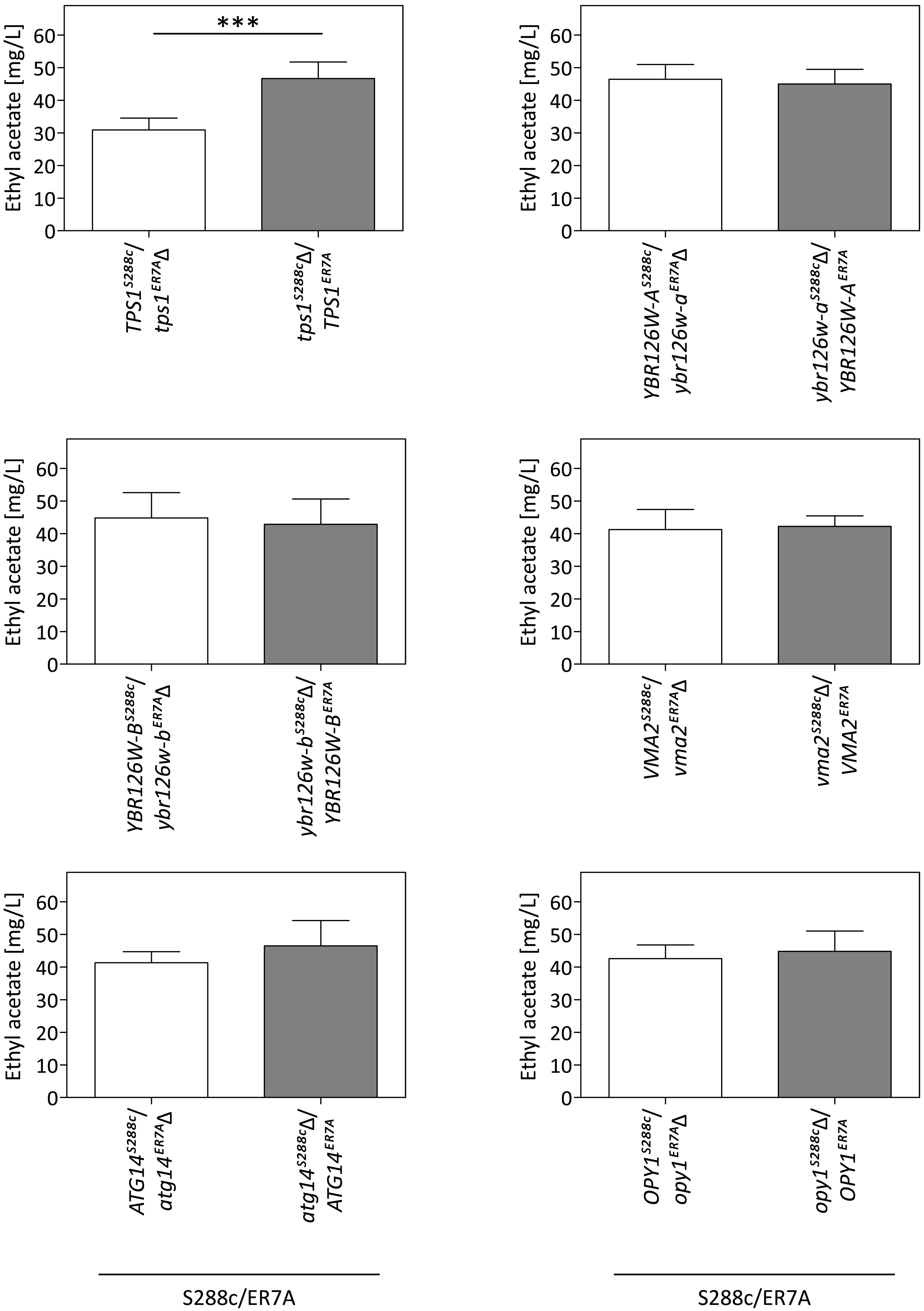

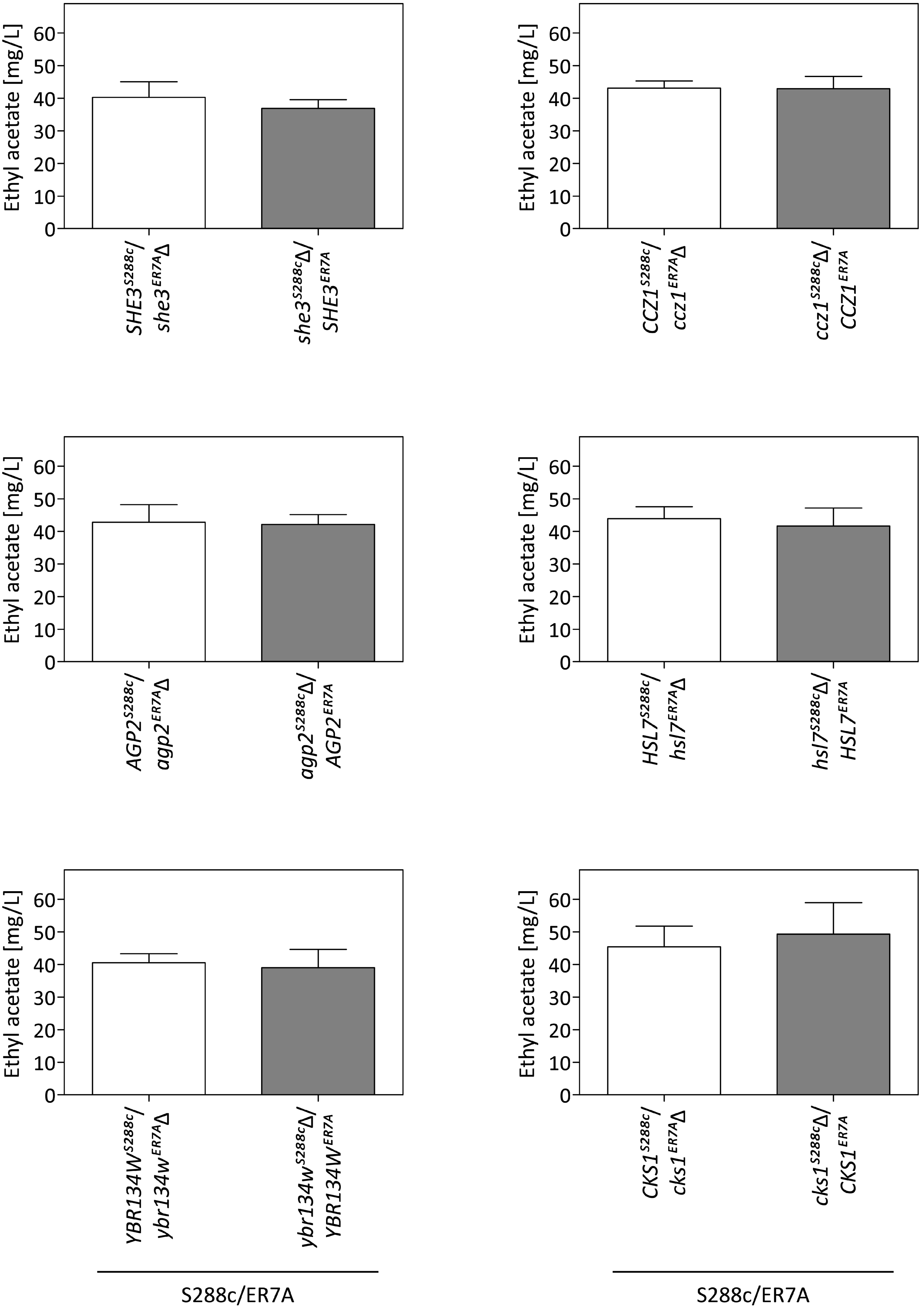
Ethyl acetate [mg/L]
0
10
20
30
40
50
60
SHE3^S288c/ she3^ER7AΔ
she3^S288cΔ/ SHE3^ER7A
CCZ1^S288c/ ccz1^ER7AΔ
ccz1^S288cΔ/ CCZ1^ER7A
AGP2^S288c/ agp2^ER7AΔ
agp2^S288cΔ/ AGP2^ER7A
HSL7^S288c/ hsl7^ER7AΔ
hsl7^S288cΔ/ HSL7^ER7A
YBR134W^S288c/ ybr134w^ER7AΔ
ybr134w^S288cΔ/ YBR134W^ER7A
CKS1^S288c/ cks1^ER7AΔ
cks1^S288cΔ/ CKS1^ER7A
S288c/ER7A
S288c/ER7A

Supplementary Figure S3c.tif @ 8,33% (Gray/8#) *

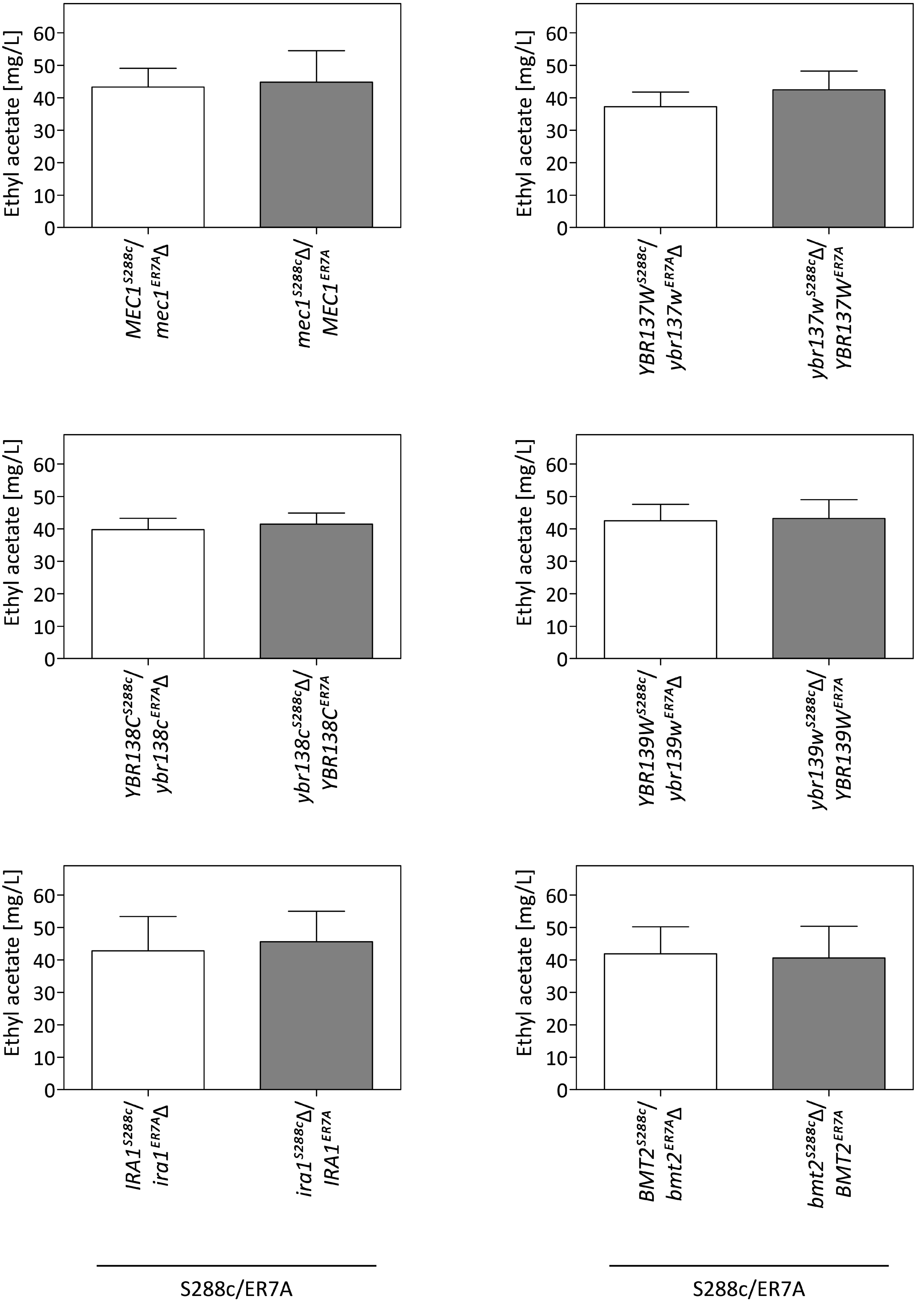

Supplementary Figure S3d.tif @ 8,33% (Gray/8#) *

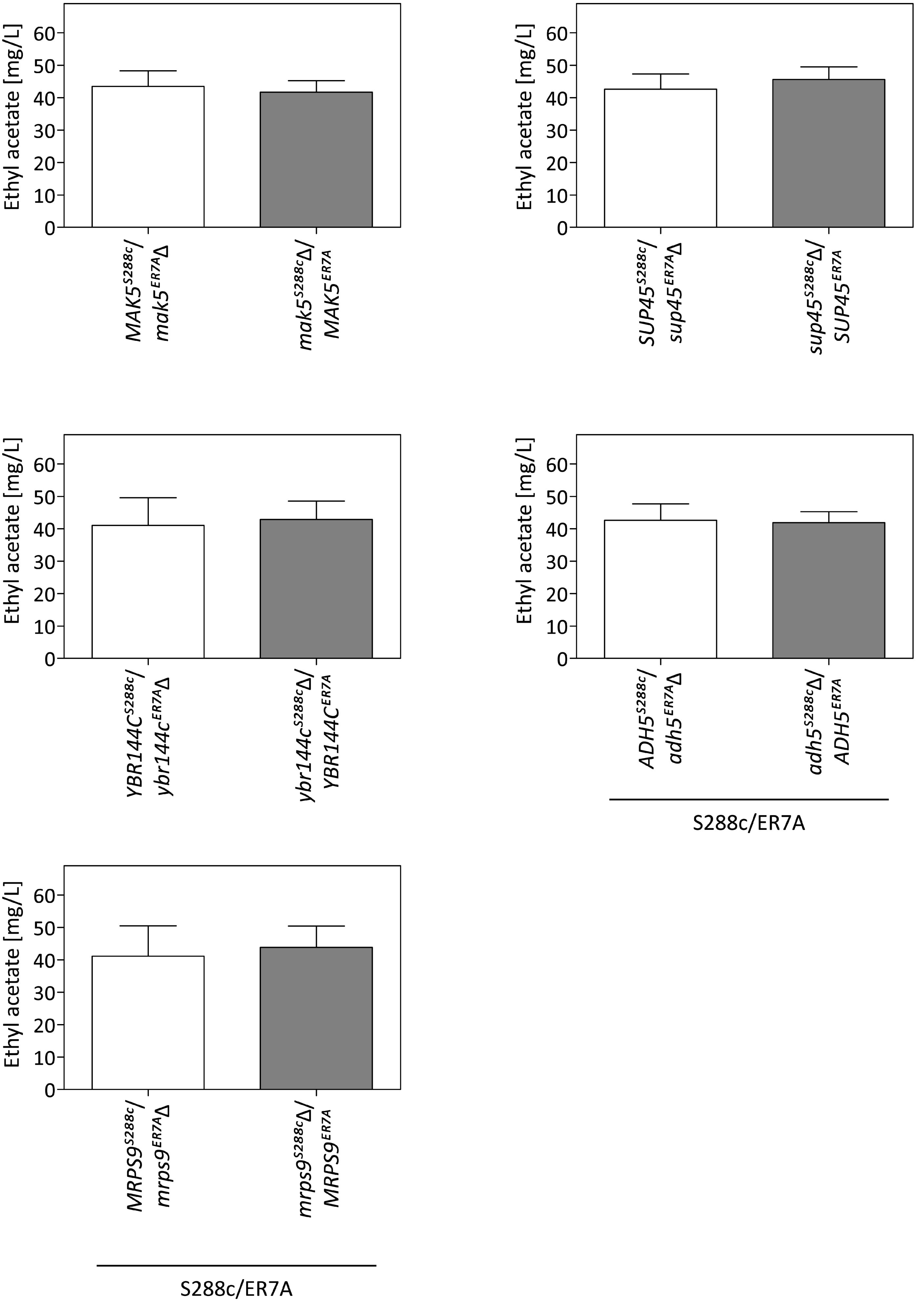

Supplementary Figure S4a.tif @ 8,33% (Gray/8#) *

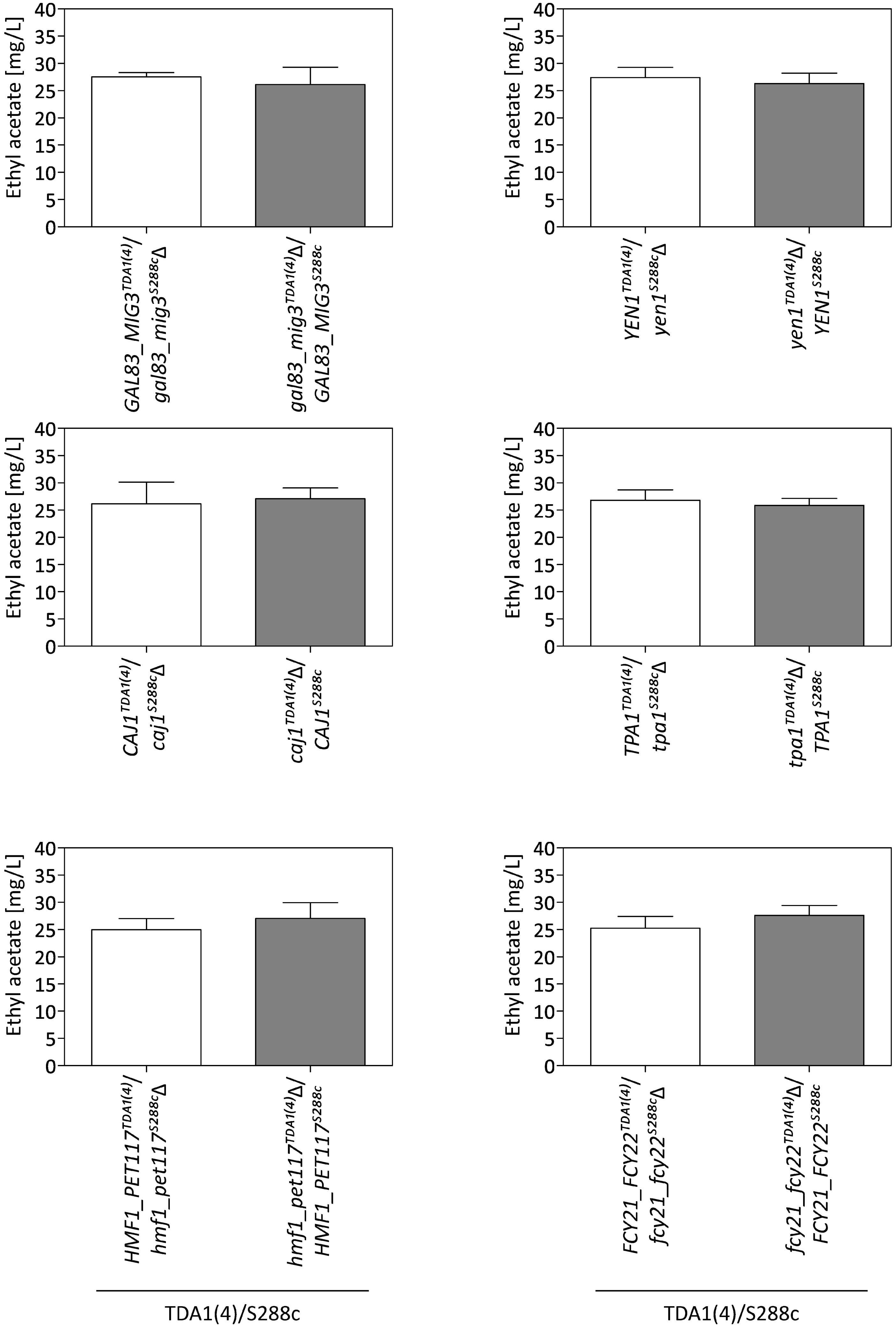

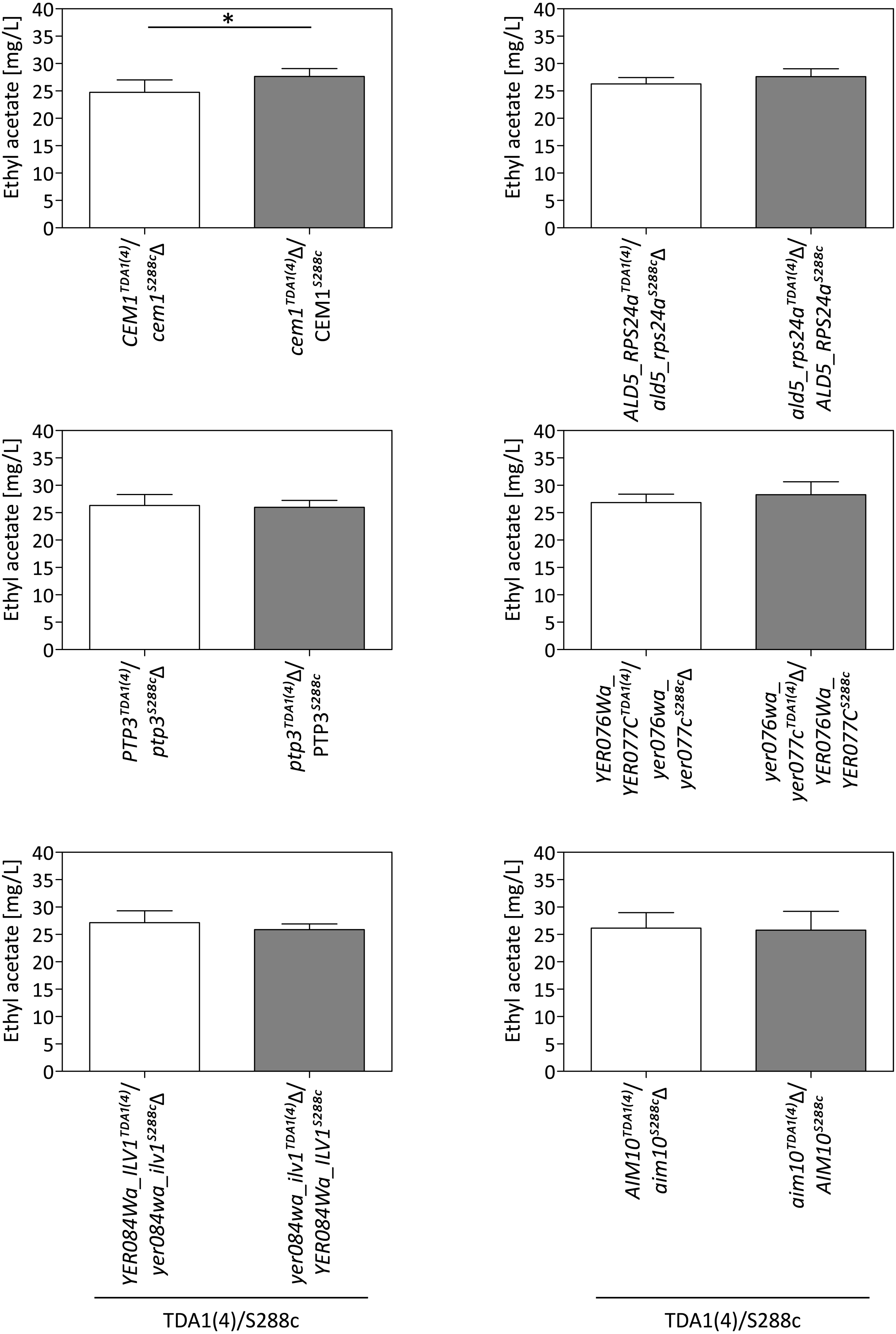

Ethyl acetate [mg/L]
0
5
10
15
20
25
30
35
40
*
CEM1TDA1(4)/ cem1S288cΔ
cem1TDA1(4)Δ/ CEM1S288c
ALD5_RPS24aTDA1(4)/ ald5_rps24aS288cΔ
ald5_rps24aTDA1(4)Δ/ ALD5_RPS24aS288c
PTP3TDA1(4)/ ptp3S288cΔ
ptp3TDA1(4)Δ/ PTP3S288c
YER076Wa_ YER077CTDA1(4)/ yer076wa_ yer077cS288cΔ
yer076wa_ yer077cTDA1(4)Δ/ YER076Wa_ YER077CS288c
YER084Wa_ILV1TDA1(4)/ yer084wa_ilv1S288cΔ
yer084wa_ilv1TDA1(4)Δ/ YER084Wa_ILV1S288c
AIM10TDA1(4)/ aim10S288cΔ
aim10TDA1(4)Δ/ AIM10S288c
TDA1(4)/S288c
TDA1(4)/S288c

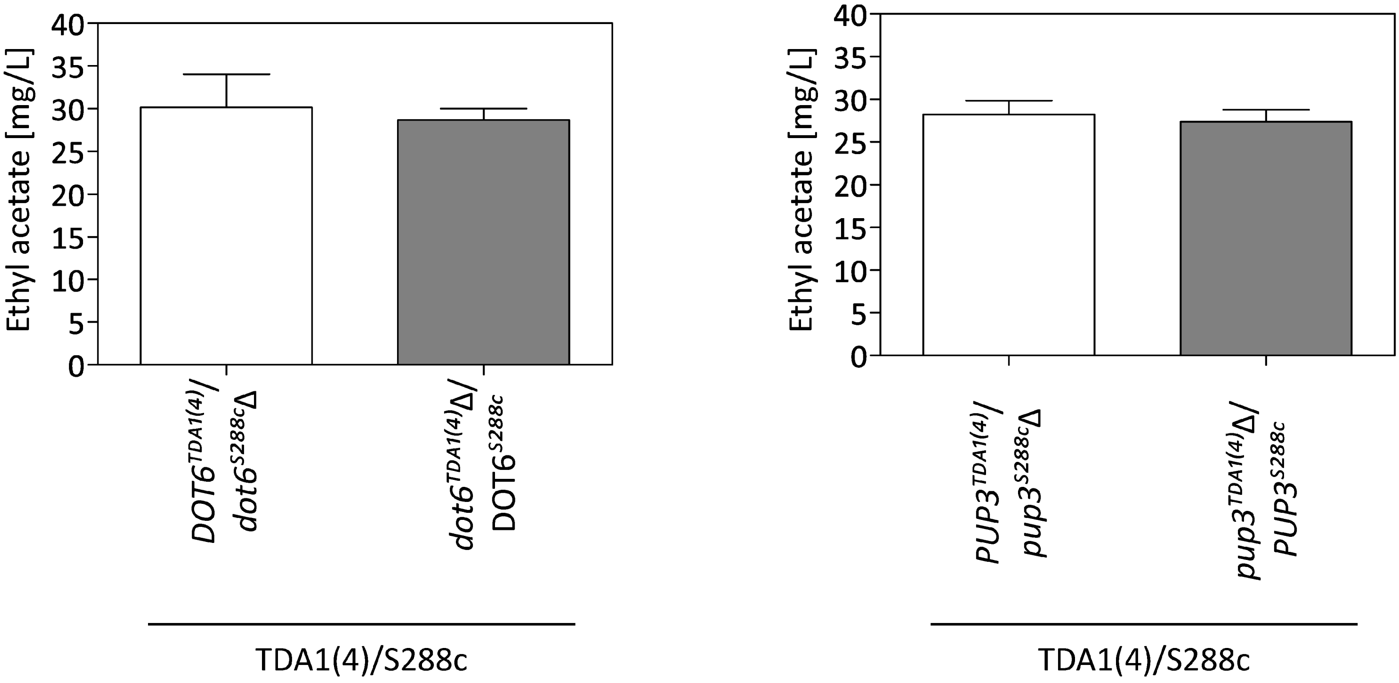
Ethyl acetate [mg/L]
40
35
30
25
20
15
10
5
0
DOT6TDA1(4)/ dot6S288cΔ
dot6TDA1(4)Δ/ DOT6S288c
TDA1(4)/S288c
Ethyl acetate [mg/L]
PUP3TDA1(4)/ pup3S288cΔ
pup3TDA1(4)Δ/ PUP3S288c
TDA1(4)/S288c

Supplementary Table S1

Variants between S288c and TDA1(4)									Natural variation at the genomic loci of the induced mutations in TDA1(4)	
CHROMOSOME	POSITION	S288c REFERENCE	INDUCED VARIANT	INFO	AMINO ACID	LOCATION IN CODON	GENE	NUCLEOTIDE IN ORF	Number of yeast strains for which the gene sequence was available on SGD	Number of yeast strains with a different nucleotide than the S288c reference
chrI	36421	ATT	AT	Upstream			YAL059W			
chrI	36421	ATT	AT	Upstream			YAL059W			
chrI	38550	C	T	Missense	363	1	YAL058W	1087	43	0
chrI	39297	C	T	Synonymous	13	3	YAL056W	39	38	0
chrI	42028	G	A	Upstream			YAL055W			
chrI	81562	C	T	Synonymous	130	3	YAL034C	390	44	0
chrI	82202	C	T	Upstream			YAL033W			
chrI	94891	G	A	Missense	69	1	YAL027W	205	43	0
chrI	105068	C	T	Missense	269	1	YAL024C	805	35	0
chrI	123020	G	A	Synonymous	932	3	YAL017W	2796	39	0
chrI	130358	G	A	Missense	363	3	YAL013W	1089	41	0
chrI	186142	G	A	Upstream			YAR031W			
chrI	192628	C	T	Missense	4	1	YAR042W	10	29	0
chrI	195391	G	A	Missense	925	1	YAR042W	2773	29	0
chrI	196241	G	A	Downstream			YAR042W			
chrI	202978	C	T	Upstream			YAR050W			
chrII	20611	C	T	Missense	228	2	YBL104C	683	39	0
chrII	25877	G	A	Missense	808	2	YBL101C	2423	43	0

chrII	42714	G	A	Synonymous	628	3	YBL097W	1884	39	0
chrII	48569	C	T	Synonymous	20	3	YBL091C	60	46	0
chrII	69651	C	T	Upstream			YBL084C			
chrII	82162	C	T	Missense	700	3	YBL076C	2100	39	0
chrII	100384	G	A	Missense	258	2	YBL064C	773	41	0
chrII	101924	G	A	Synonymous	13	3	YBL063W	39	39	0
chrII	110948	G	A	Upstream			YBL058W			
chrII	112028	G	A	Missense	198	1	YBL058W	592	43	0
chrII	117526	G	A	Upstream			YBL054W			
chrII	118473	G	A	Synonymous	295	3	YBL054W	885	35	3
chrII	130955	G	A	Synonymous	362	3	YBL047C	1086	40	4
chrII	136357	G	A	Synonymous	120	3	YBL044W	360	12	0
chrII	153234	C	T	Missense	126	2	YBL035C	377	38	0
chrII	174061	G	A	Missense	510	1	YBL024W	1528	41	0
chrII	184624	G	A	Missense	91	2	YBL019W	272	40	0
chrII	234179	G	A	Missense	2182	1	YBL004W	6544	38	0
chrII	251731	G	A	Upstream			YBR007C			
chrII	283946	G	A	Missense	70	2	YBR022W	209	47	0
chrII	293217	G	A	Missense	268	2	YBR026C	803	43	0
chrII	298769	G	A	Missense	160	1	YBR030W	478	43	0
chrII	301794	G	A	Synonymous	92	3	YBR032W	276	44	0
chrII	319064	G	A	Missense	267	1	YBR041W	799	45	0
chrII	323766	A	C	Synonymous	60	3	YBR043C	180	42	0
chrII	341146	C	T	Upstream			YBR053C			
chrII	361326	C	T	Missense	397	1	YBR060C	1189	44	0
chrII	364089	G	A	Intergenic						
chrII	365220	C	T	Missense	167	2	YBR061C	500	45	0
chrII	373217	G	A	Upstream			YBR067C			
chrII	388233	G	A	Missense	650	1	YBR074W	1948	38	0
chrII	400828	G	T	Missense	21	3	YBR080C	63	41	0
chrII	405535	G	A	Upstream			YBR081C			
chrII	461999	G	A	Upstream			YBR111W-			

							A			
chrII	467018	C	T	Upstream			YBR114W			
chrII	472616	G	A	Synonymous	437	3	YBR115C	1311	34	0
chrII	497411	G	A	Synonymous	83	3	YBR131W	249	41	0
chrII	510676	G	A	Missense	1670	2	YBR136W	5009	38	0
chrII	519234	C	T	Synonymous	2465	3	YBR140C	7395	34	0
chrII	542759	C	A	Nonsense	579	1	YBR150C	1735	38	0
chrII	589744	G	A	Missense	2	1	YBR180W	4	39	0
chrII	598938	G	A	Downstream			YBR184W			
chrII	606032	G	A	Missense	23	1	YBR190W	67	25	0
chrII	647961	G	A	Missense	26	1	YBR212W	76	41	0
chrII	651199	G	A	Upstream			YBR214W			
chrII	655032	G	A	Synonymous	531	3	YBR215W	1593	35	0
chrII	681916	T	C	Downstream			YBR231C			
chrII	692956	G	A	Missense	330	1	YBR237W	988	31	0
chrII	693071	G	A	Missense	368	2	YBR237W	1103	32	0
chrII	725409	G	A	Synonymous	318	3	YBR255W	954	37	0
chrII	733286	G	A	Missense	452	1	YBR260C	1354	42	0
chrII	745948	G	A	Missense	366	2	YBR271W	1097	47	0
chrII	780482	G	A	Synonymous	272	3	YBR289W	816	38	0
chrII	781585	G	A	Missense	640	2	YBR289W	1919	41	0
chrIII	19139	C	T	Missense	990	1	YCL061C	2968	38	0
chrIII	19570	C	T	Missense	846	2	YCL061C	2537	40	0
chrIII	21951	C	T	Synonymous	52	3	YCL061C	156	38	0
chrIII	36945	G	A	Missense	361	1	YCL051W	1081	41	0
chrIII	45048	C	T	Missense	620	1	YCL045C	1858	44	0
chrIII	48573	C	T	Upstream			YCL044C			
chrIII	65277	C	T	Missense	98	1	YCL031C	292	42	0
chrIII	79530	C	T	Synonymous	123	3	YCL024W	369	38	0
chrIII	115044	C	T	Upstream			YCR001W			
chrIII	203285	C	T	Missense	705	1	YCR042C	2113	35	0
chrIII	220881	C	T	Missense	783	2	YCR057C	2348	43	0

chrIII	232984	C	T	Downstream			YCR066W			
chrIII	236661	C	T	Upstream			YCR068W			
chrIII	246724	G	A	Upstream			YCR073W-A			
chrIII	265542	C	T	Missense	159	1	YCR088W	475	45	2
chrIII	276940	C	T	Missense	961	1	YCR092C	2881	35	0
chrIII	277179	C	T	Missense	881	2	YCR092C	2642	36	0
chrIV	20641	G	A	Missense	3	1	YDL241W	7	36	0
chrIV	31255	C	T	Missense	200	2	YDL237W	599	45	0
chrIV	37340	C	T	Missense	182	1	YDL233W	544	42	0
chrIV	42230	C	T	Synonymous	5	3	YDL231C	15	20	0
chrIV	46318	C	T	Missense	572	1	YDL227C	1714	35	0
chrIV	46363	C	T	Missense	557	1	YDL227C	1669	35	1
chrIV	47610	C	T	Missense	141	2	YDL227C	422	36	0
chrIV	61442	G	A	Synonymous	120	3	YDL222C	360	42	0
chrIV	66397	C	T	Upstream			YDL218W			
chrIV	70605	C	T	Upstream			YDL216C			
chrIV	74801	C	T	Missense	582	2	YDL214C	1745	40	0
chrIV	94930	C	T	Missense	109	2	YDL204W	326	41	0
chrIV	96160	C	T	Synonymous	598	3	YDL203C	1794	38	4
chrIV	102709	C	T	Nonsense	215	3	YDL199C	645	38	1
chrIV	127978	C	T	Missense	398	1	YDL185W	1192	35	0
chrIV	150144	C	T	Missense	1833	1	YDL171C	5497	37	0
chrIV	162897	C	T	Nonsense	86	3	YDL167C	258	42	0
chrIV	197966	C	T	Missense	71	1	YDL145C	211	38	0
chrIV	217359	G	A	Upstream			YDL136W			
chrIV	229542	C	T	Upstream			YDL130W			
chrIV	238161	C	T	Missense	168	3	YDL126C	504	38	0
chrIV	241698	C	T	Missense	94	2	YDL123W	281	43	0
chrIV	271809	CTGTT	CT	Missense	30	2	YDL106C	89	42	31
chrIV	273928	C	T	Missense	317	1	YDL104C	949	36	0
chrIV	314716	C	T	Synonymous	11	3	YDL079C	33	42	0

chrIV	315358	C	T	Synonymous	344	2	YDL078C	1031	43	0
chrIV	315472	C	T	Missense	306	2	YDL078C	917	43	0
chrIV	319321	G	A	Missense	267	2	YDL077C	800	40	1
chrIV	319399	C	T	Missense	241	2	YDL077C	722	40	1
chrIV	319554	G	A	Synonymous	189	3	YDL077C	567	40	0
chrIV	320951	C	T	Missense	201	2	YDL076C	602	45	0
chrIV	372063	C	T	Upstream			YDL045W-A			
chrIV	373590	C	T	Missense	7	1	YDL045C	19	43	0
chrIV	392150	C	T	Upstream			YDL035C			
chrIV	394804	G	A	Synonymous	196	3	YDL031W	588	42	0
chrIV	400194	G	A	Synonymous	244	3	YDL029W	732	46	0
chrIV	415428	C	T	Synonymous	427	3	YDL020C	1281	40	0
chrIV	417121	G	A	Upstream			YDL020C			
chrIV	428180	G	A	Missense	273	1	YDL014W	817	42	0
chrIV	430025	G	A	Missense	320	2	YDL013W	959	44	0
chrIV	449332	C	T	Downstream			YDL001W			
chrIV	460912	C	T	Synonymous	112	3	YDR006C	336	44	0
chrIV	477605	C	T	Upstream			YDR014W-A			
chrIV	574839	G	A	Missense	226	1	YDR061W	676	41	0
chrIV	588179	C	T	Synonymous	67	3	YDR070C	201	21	0
chrIV	588469	C	T	Upstream			YDR070C			
chrIV	593446	C	T	Upstream			YDR074W			
chrIV	614043	G	A	Upstream			YDR084C			
chrIV	615582	C	T	Missense	190	2	YDR085C	569	36	0
chrIV	639723	G	A	Missense	862	2	YDR096W	2585	39	0
chrIV	656007	G	A	Synonymous	487	3	YDR101C	1461	42	0
chrIV	659880	G	A	Missense	511	1	YDR103W	1531	45	0
chrIV	664368	TTCT	TTT	Frameshift	180	3	YDR104C	540	38	0
chrIV	686384	G	A	Missense	168	2	YDR118W	503	40	0
chrIV	696877	G	A	Synonymous	726	3	YDR122W	2178	42	0

chrIV	708446	G	A	Synonymous	1321	3	YDR127W	3963	37	0
chrIV	728526	C	T	Synonymous	90	1	YDR137W	268	40	0
chrIV	730565	C	T	Upstream			YDR138W			
chrIV	752366	G	A	Missense	246	1	YDR147W	736	44	0
chrIV	763563	G	A	Missense	2646	1	YDR150W	7936	7	0
chrIV	781816	G	A	Missense	132	1	YDR163W	394	43	0
chrIV	787848	G	A	Synonymous	458	3	YDR166C	1374	44	0
chrIV	795881	G	A	Intergenic						
chrIV	797282	G	A	Synonymous	1647	3	YDR170C	4941	38	0
chrIV	800998	C	T	Missense	409	1	YDR170C	1225	34	0
chrIV	839236	G	A	Missense	282	2	YDR189W	845	37	0
chrIV	856734	G	A	Missense	140	1	YDR201W	418	46	0
chrIV	858338	G	A	Missense	68	1	YDR204W	202	44	0
chrIV	861514	A	T	Synonymous	723	3	YDR205W	2169	44	0
chrIV	885259	G	A	Missense	178	2	YDR211W	533	40	8
chrIV	898230	C	T	Missense	1066	1	YDR216W	3196	36	0
chrIV	899321	G	A	Downstream			YDR217C			
chrIV	917143	G	A	Missense	220	1	YDR226W	658	45	0
chrIV	919590	G	A	Missense	674	1	YDR227W	2020	40	2
chrIV	996753	C	T	Missense	524	2	YDR264C	1571	43	0
chrIV	1020084	G	A	Synonymous	239	3	YDR279W	717	43	1
chrIV	1037946	G	A	Missense	251	2	YDR288W	752	47	0
chrIV	1077762	C	T	Missense	633	2	YDR307W	1898	42	0
chrIV	1080961	C	T	Upstream			YDR309C			
chrIV	1134363	G	A	Missense	357	1	YDR333C	1069	44	0
chrIV	1145176	G	A	Missense	29	1	YDR336W	85	40	0
chrIV	1174505	G	A	Upstream			YDR349C			
chrIV	1186118	G	A	Synonymous	84	3	YDR355C	252	22	0
chrIV	1220103	G	A	Missense	231	1	YDR371W	691	43	0
chrIV	1222718	G	A	Upstream			YDR373W			
chrIV	1255560	G	A	Missense	430	1	YDR390C	1288	43	0
chrIV	1267951	G	A	Missense	161	1	YDR398W	481	44	0

chrIV	1277754	G	A	Missense	37	1	YDR405W	109	45	0
chrIV	1277862	G	A	Missense	73	1	YDR405W	217	45	0
chrIV	1293719	C	T	Missense	226	1	YDR411C	676	45	0
chrIV	1353861	G	A	Missense	46	2	YDR446W	137	44	0
chrIV	1357082	G	A	Missense	340	1	YDR448W	1018	44	0
chrIV	1363949	G	A	Missense	358	1	YDR452W	1072	43	0
chrIV	1413043	G	A	Missense	224	2	YDR477W	671	42	0
chrIV	1461193	G	A	Missense	121	2	YDR506C	362	40	0
chrIV	1478684	G	A	Missense	26	2	YDR518W	77	44	0
chrIV	1496531	C	A	Synonymous	6	3	YDR529C	18	47	0
chrIV	1505979	G	A	Intergenic						
chrIV	1514519	G	A	Intergenic						
chrIV	1515033	GTAA	GTA	Intergenic						
chrV	6733	G	A	Downstream			YEL074W			
chrV	9912	G	A	Intergenic						
chrV	28451	G	A	Synonymous	265	3	YEL065W	795	40	0
chrV	44101	G	A	Missense	284	1	YEL058W	850	44	0
chrV	45035	G	A	Synonymous	229	3	YEL057C	687	40	0
chrV	56869	G	A	Missense	100	2	YEL052W	299	44	0
chrV	74306	G	A	Missense	179	2	YEL042W	536	43	0
chrV	77803	G	A	Upstream			YEL040W			
chrV	79077	G	A	Missense	342	2	YEL040W	1025	41	0
chrV	88545	G	A	Missense	537	1	YEL032W	1609	42	0
chrV	93797	G	A	Synonymous	1180	3	YEL031W	3540	38	1
chrV	108762	ACT	AT	Frameshift	597	1	YEL023C	1789	36	0
chrV	111911	C	T	Missense	164	2	YEL022W	491	36	0
chrV	134356	G	A	Missense	413	1	YEL011W	1237	43	0
chrV	150364	G	A	Synonymous	205	3	YEL001C	615	45	0
chrV	163235	C	T	Synonymous	171	3	YER006W	513	43	0
chrV	187586	C	T	Upstream			YER016W			
chrV	198615	C	T	Upstream			YER022W			
chrV	199795	G	A	Synonymous	328	3	YER022W	984	43	0

chrV	210408	C	T	Upstream			YER027C			
chrV	212110	G	A	Upstream			YER028C			
chrV	232604	G	A	Synonymous	48	3	YER041W	144	39	0
chrV	248163	C	T	Upstream			YER048C			
chrV	253489	G	A	Missense	588	1	YER049W	1762	46	0
chrV	271433	G	A	Upstream			YER058W			
chrV	276276	G	A	Upstream			YER060W-A			
chrV	278369	G	A	Missense	420	1	YER061C	1258	39	0
chrV	312128	G	A	Upstream			YER075C			
chrV	314194	G	A	Upstream			YER076C			
chrV	328199	C	T	Upstream			YER086W			
chrV	331336	G	A	Missense	254	2	YER087W	761	46	0
chrV	335100	G	A	Missense	30	2	YER088C	89	37	0
chrV	349110	T	A	Missense	79	3	YER094C	237	44	0
chrV	401669	G	A	Missense	179	1	YER120W	535	42	0
chrV	408292	C	T	Missense	258	1	YER124C	772	45	0
chrV	421093	C	T	Downstream			YER130C			
chrV	426714	G	A	Missense	1246	1	YER132C	3736	38	0
chrV	438115	G	A	Missense	77	2	YER135C	230	42	0
chrV	442256	G	A	Upstream			YER137C			
chrV	452622	G	A	Nonsense	353	2	YER140W	1058	45	0
chrV	464940	C	T	Upstream			YER148W			
chrV	475095	G	A	Missense	26	1	YER154W	76	47	0
chrV	489775	A	G	Synonymous	268	3	YER158C	804	45	0
chrV	491377	C	T	Upstream			YER158W-A			
chrV	501956	C	T	Synonymous	313	3	YER162C	939	42	0
chrV	505022	G	A	Upstream			YER164W			
chrV	521324	G	A	Missense	449	2	YER168C	1346	39	0
chrV	526043	G	A	Missense	24	1	YER170W	70	46	0
chrV	550184	C	T	Missense	115	2	YER180C	344	46	0

chrVI	44897	G	A	Missense	222	1	YFL044C	664	44	0
chrVI	56198	G	A	Upstream			YFL037W			
chrVI	72062	G	A	Missense	789	2	YFL033C	2366	37	0
chrVI	81537	G	A	Synonymous	126	1	YFL027C	376	43	0
chrVI	81544	G	A	Synonymous	123	3	YFL027C	369	43	0
chrVI	85867	G	A	Synonymous	456	3	YFL025C	1368	43	0
chrVI	97030	G	A	Synonymous	355	3	YFL021W	1065	36	0
chrVI	98766	ATT	AT	Intergenic						
chrVI	107817	ATGTGT	ATGT	Frameshift	7	1	YFL013W-A	19	8	5
chrVI	165291	G	A	Synonymous	75	3	YFR010W	225	45	0
chrVI	180066	G	A	Synonymous	226	3	YFR016C	678	35	0
chrVI	184418	G	A	Upstream			YFR019W			
chrVI	214300	G	A	Missense	330	2	YFR030W	989	38	0
chrVI	215352	G	A	Missense	681	1	YFR030W	2041	35	0
chrVI	227354	G	A	Upstream			YFR036W-A			
chrVI	231356	G	A	Synonymous	659	3	YFR038W	1977	41	0
chrVI	238862	G	A	Synonymous	135	3	YFR042W	405	43	0
chrVI	247181	C	T	Synonymous	346	1	YFR048W	1036	43	0
chrVI	247626	C	T	Missense	494	2	YFR048W	1481	41	0
chrVI	256541	G	A	Intergenic						
chrVI	264835	G	A	Missense	211	2	YFR055W	632	44	0
chrVII	23812	TAA	TA	Upstream			YGL253W			
chrVII	27198	G	A	Missense	96	2	YGL252C	287	45	0
chrVII	41932	G	A	Synonymous	145	3	YGL244W	435	42	0
chrVII	45190	G	A	Missense	3	2	YGL242C	8	42	0
chrVII	45688	G	A	Missense	82	1	YGL241W	244	36	0
chrVII	58277	G	A	Missense	599	2	YGL234W	1796	41	0
chrVII	58360	G	A	Missense	627	1	YGL234W	1879	41	0
chrVII	61620	G	A	Synonymous	833	3	YGL233W	2499	36	6
chrVII	62140	G	A	Downstream			YGL233W			

chrVII	62562	G	A	Missense	144	1	YGL232W	430	33	0
chrVII	86187	G	A	Missense	435	1	YGL216W	1303	41	0
chrVII	91059	G	A	Synonymous	63	3	YGL213C	189	42	0
chrVII	95202	G	A	Upstream			YGL209W			
chrVII	108450	G	A	Missense	98	2	YGL205W	293	44	0
chrVII	125308	G	A	Nonsense	204	2	YGL197W	611	39	0
chrVII	130739	G	A	Missense	286	2	YGL196W	857	42	0
chrVII	145006	G	A	Missense	67	1	YGL191W	199	44	0
chrVII	169669	G	A	Missense	560	1	YGL178W	1678	33	0
chrVII	208263	G	A	Missense	411	1	YGL158W	1231	46	0
chrVII	215162	G	A	Missense	361	2	YGL155W	1082	41	0
chrVII	229861	G	A	Upstream			YGL145W			
chrVII	230041	C	T	Upstream			YGL145W			
chrVII	242575	G	A	Missense	813	2	YGL140C	2438	37	0
chrVII	261030	G	A	Synonymous	1108	3	YGL133W	3324	40	0
chrVII	261215	G	A	Missense	1170	2	YGL133W	3509	40	0
chrVII	268027	C	T	Missense	350	2	YGL129C	1049	44	0
chrVII	274919	G	A	Missense	599	2	YGL124C	1796	33	9
chrVII	280770	G	A	Upstream			YGL122C			
chrVII	289519	G	A	Upstream			YGL116W			
chrVII	310196	C	T	Upstream			YGL103W			
chrVII	311027	C	T	Upstream			YGL104C			
chrVII	313821	G	A	Synonymous	196	3	YGL100W	588	46	0
chrVII	322982	G	A	Missense	401	1	YGL097W	1201	42	0
chrVII	342794	G	A	Synonymous	83	3	YGL091C	249	44	0
chrVII	344069	G	A	Missense	251	1	YGL090W	751	45	0
chrVII	346681	C	T	Missense	47	1	YGL087C	139	36	0
chrVII	349853	G	A	Missense	80	1	YGL085W	238	42	0
chrVII	356628	G	A	Missense	268	1	YGL082W	802	48	0
chrVII	369308	G	A	Missense	186	1	YGL073W	556	40	0
chrVII	369662	G	A	Missense	304	1	YGL073W	910	42	0
chrVII	374739	C	T	Missense	30	2	YGL070C	89	44	0

chrVII	375185	C	T	Synonymous	110	3	YGL069C	330	23	0
chrVII	382045	C	T	Synonymous	388	3	YGL064C	1164	46	0
chrVII	383615	G	A	Upstream			YGL063W			
chrVII	421931	C	T	Intergenic						
chrVII	435307	G	A	Upstream			YGL033W			
chrVII	435360	C	T	Upstream			YGL033W			
chrVII	443164	C	T	Upstream			YGL028C			
chrVII	461659	G	A	Upstream			YGL016W			
chrVII	465830	C	T	Upstream			YGL014W			
chrVII	482216	G	A	Missense	152	3	YGL008C	456	34	3
chrVII	525761	C	T	Missense	178	2	YGR019W	533	45	0
chrVII	568592	G	A	Intergenic						
chrVII	602872	G	A	Nonsense	404	3	YGR056W	1212	40	0
chrVII	622587	C	T	Missense	734	1	YGR067C	2200	43	0
chrVII	630072	G	A	Missense	756	2	YGR070W	2267	42	0
chrVII	697316	G	A	Downstream			YGR103W			
chrVII	751436	G	A	Synonymous	803	3	YGR130C	2409	43	0
chrVII	772580	G	A	Missense	43	1	YGR142W	127	39	0
chrVII	773076	A	C	Missense	208	2	YGR142W	623	39	0
chrVII	789154	C	T	Missense	42	1	YGR149W	124	44	0
chrVII	801642	C	A	Missense	366	2	YGR156W	1097	44	0
chrVII	802515	G	A	Missense	26	1	YGR157W	76	40	0
chrVII	827035	G	A	Upstream			YGR163W			
chrVII	853839	C	T	Missense	353	1	YGR179C	1057	46	0
chrVII	859735	G	A	Upstream			YGR183C			
chrVII	878017	C	T	Missense	59	2	YGR189C	176	43	0
chrVII	902460	G	A	Upstream			YGR200C			
chrVII	934951	C	T	Missense	804	2	YGR218W	2411	41	0
chrVII	966065	GTT	GT	Frameshift	874	2	YGR238C	2621	38	0
chrVII	1017053	C	T	Missense	236	2	YGR262C	707	45	0
chrVII	1024282	G	A	Missense	543	1	YGR266W	1627	42	0
chrVII	1057142	G	A	Missense	1440	2	YGR281W	4319	39	2

chrVII	1060019	C	T	Missense	8	1	YGR283C	22	38	0
chrVIII	41147	G	A	Missense	355	2	YHL030W	1064	39	0
chrVIII	44833	C	T	Missense	1584	1	YHL030W	4750	43	1
chrVIII	59664	G	A	Missense	967	2	YHL023C	2900	44	1
chrVIII	108115	C	T	Missense	76	2	YHR001W-A	227	21	0
chrVIII	124148	C	T	Synonymous	511	3	YHR009C	1533	45	0
chrVIII	131220	C	T	Missense	76	2	YHR013C	227	46	0
chrVIII	138969	G	A	Synonymous	92	3	YHR017W	276	41	0
chrVIII	153418	G	A	Missense	585	1	YHR023W	1753	38	0
chrVIII	171147	C	T	Synonymous	608	3	YHR031C	1824	39	0
chrVIII	172511	C	T	Missense	154	1	YHR031C	460	39	0
chrVIII	180906	G	A	Missense	188	1	YHR036W	562	47	0
chrVIII	185512	C	T	Missense	433	2	YHR039C	1298	41	0
chrVIII	189815	C	T	Upstream			YHR042W			
chrVIII	191683	G	A	Missense	381	1	YHR042W	1141	41	0
chrVIII	197182	C	T	Synonymous	544	3	YHR045W	1632	37	0
chrVIII	198528	G	A	Upstream			YHR046C			
chrVIII	203617	C	T	Upstream			YHR048W			
chrVIII	205160	G	A	Missense	185	2	YHR048W	554	45	0
chrVIII	219735	C	T	Synonymous	50	3	YHR058C	150	47	13
chrVIII	220946	G	A	Missense	74	3	YHR060W	222	47	0
chrVIII	256725	G	A	Synonymous	122	3	YHR078W	366	45	0
chrVIII	264991	C	T	Synonymous	616	3	YHR080C	1848	42	0
chrVIII	278184	G	A	Missense	11	2	YHR086W	32	44	0
chrVIII	304030	G	A	Missense	424	1	YHR099W	1270	36	0
chrVIII	316256	C	T	Upstream			YHR102W			
chrVIII	334607	C	T	Missense	353	1	YHR112C	1057	45	0
chrVIII	344804	C	T	Synonymous	275	3	YHR118C	825	42	0
chrVIII	372035	G	A	Missense	96	2	YHR134W	287	42	0
chrVIII	386057	C	T	Missense	183	2	YHR143W	548	41	0
chrVIII	396102	C	T	Synonymous	186	3	YHR149C	558	46	0

chrVIII	418786	G	A	Missense	413	2	YHR159W	1238	44	0
chrVIII	420042	C	T	Missense	11	1	YHR160C	31	45	0
chrVIII	432326	G	A	Synonymous	1541	3	YHR165C	4623	42	0
chrVIII	442258	C	T	Synonymous	26	3	YHR169W	78	45	0
chrVIII	442496	G	A	Missense	106	1	YHR169W	316	45	0
chrVIII	447339	G	A	Missense	543	1	YHR171W	1627	43	0
chrVIII	476374	C	T	Synonymous	1433	3	YHR186C	4299	31	0
chrVIII	482846	C	T	Missense	331	2	YHR188C	992	43	0
chrVIII	483385	C	T	Synonymous	151	3	YHR188C	453	43	0
chrVIII	483427	C	T	Synonymous	137	3	YHR188C	411	43	0
chrVIII	495237	G	A	Missense	448	1	YHR197W	1342	43	0
chrVIII	495855	G	A	Missense	654	1	YHR197W	1960	43	0
chrVIII	514200	G	A	Missense	490	2	YHR206W	1469	36	0
chrIX	36461	G	A	Upstream			YIL162W			
chrIX	56779	G	A	Missense	79	1	YIL152W	235	44	0
chrIX	66107	GTT	GT	Frameshift	653	3	YIL149C	1959	34	0
chrIX	78057	G	A	Upstream			YIL144W			
chrIX	95408	C	T	Missense	323	2	YIL135C	968	42	0
chrIX	104008	G	A	Synonymous	409	3	YIL130W	1227	16	0
chrIX	109423	C	T	Missense	1272	2	YIL129C	3815	37	0
chrIX	124850	G	A	Missense	721	2	YIL125W	2162	38	0
chrIX	126856	G	A	Missense	218	2	YIL124W	653	45	0
chrIX	143960	G	A	Missense	345	1	YIL116W	1033	39	1
chrIX	167774	C	T	Missense	623	2	YIL105C	1868	38	0
chrIX	217725	C	T	Downstream			YIL076W			
chrIX	227078	G	A	Synonymous	159	3	YIL072W	477	35	0
chrIX	227708	G	A	Synonymous	369	3	YIL072W	1107	37	0
chrIX	236100	G	A	Missense	554	2	YIL067C	1661	36	0
chrIX	237027	G	A	Missense	245	2	YIL067C	734	35	0
chrIX	237923	G	A	Upstream			YIL067C			
chrIX	250764	G	A	Synonymous	258	3	YIL056W	774	34	0
chrIX	252212	C	T	Missense	572	1	YIL055C	1714	34	0

chrIX	252648	C	T	Synonymous	426	3	YIL055C	1278	35	0
chrIX	271141	G	A	Upstream			YIL045W			
chrIX	281229	G	A	Missense	475	2	YIL038C	1424	36	0
chrIX	281304	C	T	Missense	450	2	YIL038C	1349	36	0
chrIX	301509	C	T	Upstream			YIL028W			
chrIX	304272	C	T	Upstream			YIL027C			
chrIX	372673	C	T	Missense	657	2	YIR007W	1970	31	19
chrIX	382205	G	A	Synonymous	86	3	YIR015W	258	47	0
chrIX	388471	C	T	Intergenic						
chrIX	390078	C	T	Missense	1200	1	YIR019C	3598	0	0
chrIX	393172	C	T	Nonsense	168	3	YIR019C	504	0	0
chrIX	396963	C	T	Upstream			YIR021W			
chrIX	403009	C	T	Synonymous	161	3	YIR024C	483	45	2
chrIX	405367	C	T	Missense	201	1	YIR026C	601	43	0
chrX	26642	G	A	Nonsense	44	1	YJL215C	130	29	0
chrX	30394	G	A	Intergenic						
chrX	65674	G	A	Missense	624	1	YJL197W	1870	38	3
chrX	69151	G	A	Upstream			YJL194W			
chrX	76761	GAA	GA	Upstream			YJL188C			
chrX	87215	G	A	Missense	519	2	YJL181W	1556	30	0
chrX	94826	C	T	Upstream			YJL174W			
chrX	105452	G	A	Missense	147	1	YJL167W	439	47	0
chrX	134883	G	A	Missense	351	1	YJL153C	1051	43	0
chrX	155896	C	T	Missense	78	1	YJL137C	232	43	0
chrX	161835	TAAA	TAA	Upstream			YJL132W			
chrX	167857	G	A	Missense	1504	2	YJL130C	4511	38	0
chrX	216665	G	A	Synonymous	215	3	YJL109C	645	41	0
chrX	238356	G	A	Missense	365	2	YJL100W	1094	44	18
chrX	239503	G	A	Synonymous	30	3	YJL099W	90	44	0
chrX	258264	G	A	Missense	281	2	YJL092W	842	39	0
chrX	269684	G	A	Missense	296	1	YJL088W	886	45	0
chrX	278869	G	A	Missense	10	2	YJL083W	29	39	0

chrX	323669	C	T	Missense	95	2	YJL060W	284	44	0
chrX	331839	C	T	Missense	412	2	YJL056C	1235	41	0
chrX	340861	G	A	Missense	359	1	YJL051W	1075	40	0
chrX	370317	C	T	Synonymous	1161	3	YJL039C	3483	42	0
chrX	386266	G	A	Upstream			YJL032W			
chrX	395319	C	T	Synonymous	451	3	YJL025W	1353	42	0
chrX	397496	C	T	Synonymous	301	3	YJL023C	903	46	20
chrX	411415	C	T	Missense	662	2	YJL012C	1985	45	0
chrX	414040	G	A	Upstream			YJL012C			
chrX	423157	C	T	Missense	318	2	YJL006C	953	37	0
chrX	459821	C	T	Missense	200	1	YJR012C	598	21	1
chrX	461186	C	T	Missense	269	1	YJR013W	805	38	0
chrX	467364	G	A	Synonymous	306	3	YJR019C	918	43	0
chrX	495160	T	A	Missense	431	2	YJR033C	1292	42	2
chrX	515699	G	A	Missense	549	1	YJR042W	1645	42	0
chrX	516502	GCT	GT	Frameshift	337	2	YJR043C	1010	42	0
chrX	516790	C	T	Missense	242	1	YJR043C	724	42	0
chrX	522071	G	A	Synonymous	8	3	YJR046W	24	40	0
chrX	569207	C	T	Missense	65	1	YJR069C	193	44	0
chrX	591555	C	T	Missense	925	1	YJR090C	2773	40	0
chrX	608108	C	T	Upstream			YJR094W-A			
chrX	615829	C	T	Missense	85	2	YJR099W	254	46	0
chrX	635577	G	A	Missense	655	1	YJR110W	1963	37	0
chrX	658447	C	T	Missense	81	3	YJR126C	243	40	0
chrX	678106	C	T	Missense	201	1	YJR136C	601	46	0
chrX	717252	C	T	Upstream			YJR151W-A			
chrXI	18498	C	T	Upstream			YKL218C			
chrXI	54293	G	A	Missense	197	2	YKL204W	590	42	0
chrXI	64371	C	T	Missense	184	2	YKL202W	551	4	0
chrXI	119775	C	T	Missense	328	2	YKL175W	983	43	0

chrXI	177686	C	T	Synonymous	302	3	YKL143W	906	47	0
chrXI	181992	C	T	Missense	405	2	YKL140W	1214	39	0
chrXI	207972	G	A	Upstream			YKL125W			
chrXI	237327	AT	ATT	Upstream			YKL106W			
chrXI	278370	G	A	Synonymous	30	3	YKL086W	90	44	0
chrXI	297400	G	A	Missense	324	2	YKL073W	971	42	26
chrXI	300748	C	T	Synonymous	389	3	YKL072W	1167	42	0
chrXI	323720	C	T	Missense	165	1	YKL062W	493	44	0
chrXI	353813	G	A	Synonymous	107	3	YKL045W	321	43	0
chrXI	356495	C	T	Synonymous	58	3	YKL044W	174	35	0
chrXI	360614	G	A	Missense	158	1	YKL041W	472	43	0
chrXI	418897	G	A	Synonymous	196	3	YKL012W	588	42	0
chrXI	451568	GGAACACG	GG	Missense	45	3	YKR007W	135	48	0
chrXI	487022	C	T	Synonymous	117	3	YKR024C	351	41	0
chrXI	516455	G	A	Missense	465	1	YKR039W	1393	46	0
chrXI	529895	G	A	Missense	694	2	YKR050W	2081	42	0
chrXI	530433	G	A	Synonymous	873	3	YKR050W	2619	40	0
chrXI	579816	C	T	Upstream			YKR075W-A			
chrXI	597138	C	T	Missense	585	1	YKR084C	1753	42	0
chrXI	626243	G	A	Upstream			YKR096W			
chrXI	650507	G	A	Intergenic						
chrXII	23405	G	A	Upstream			YLL058W			
chrXII	33220	G	A	Missense	662	2	YLL054C	1985	39	1
chrXII	38621	G	A	Missense	284	2	YLL051C	851	44	0
chrXII	38638	C	T	Synonymous	278	3	YLL051C	834	44	0
chrXII	39161	G	A	Missense	104	2	YLL051C	311	44	0
chrXII	40496	C	T	Upstream			YLL049W			
chrXII	46949	C	T	Missense	172	2	YLL046C	515	37	0
chrXII	60028	G	A	Synonymous	1206	3	YLL040C	3618	41	0
chrXII	60801	C	T	Missense	949	1	YLL040C	2845	40	0
chrXII	63504	C	T	Missense	48	1	YLL040C	142	36	0

chrXII	76842	G	A	Upstream			YLL032C			
chrXII	91236	G	A	Missense	872	1	YLL026W	2614	41	0
chrXII	122348	C	T	Synonymous	789	3	YLL013C	2367	41	0
chrXII	128090	G	A	Missense	190	1	YLL011W	568	44	0
chrXII	137081	G	A	Missense	166	1	YLL006W	496	41	0
chrXII	146201	C	T	Upstream			YLL002W			
chrXII	180074	G	A	Upstream			YLR019W			
chrXII	244965	C	T	Missense	70	1	YLR049C	208	47	0
chrXII	245555	G	A	Upstream			YLR049C			
chrXII	252849	C	T	Missense	78	1	YLR055C	232	35	0
chrXII	276235	G	A	Synonymous	798	1	YLR071C	2392	37	16
chrXII	276317	C	T	Synonymous	770	3	YLR071C	2310	37	0
chrXII	311346	C	T	Synonymous	1462	3	YLR087C	4386	35	7
chrXII	315110	C	T	Missense	208	1	YLR087C	622	38	0
chrXII	316149	G	A	Missense	15	1	YLR088W	43	43	0
chrXII	320503	G	A	Upstream			YLR090W			
chrXII	331492	G	A	Synonymous	208	3	YLR095C	624	46	0
chrXII	344051	C	T	Missense	631	1	YLR103C	1891	38	0
chrXII	356466	C	T	Missense	2425	1	YLR106C	7273	28	0
chrXII	362130	C	T	Missense	537	1	YLR106C	1609	27	0
chrXII	364529	G	A	Synonymous	138	3	YLR107W	414	42	2
chrXII	381706	G	A	Synonymous	295	3	YLR116W	885	44	0
chrXII	381992	G	A	Missense	391	1	YLR116W	1171	43	0
chrXII	385503	C	T	Upstream			YLR119W			
chrXII	386840	C	T	Missense	461	1	YLR120C	1381	40	0
chrXII	388467	G	A	Upstream			YLR120W-A			
chrXII	390439	C	T	Upstream			YLR121C			
chrXII	417082	G	A	Missense	26	2	YLR137W	77	39	0
chrXII	435393	C	T	Synonymous	251	3	YLR148W	753	42	0
chrXII	439225	C	T	Missense	200	2	YLR149C	599	40	0
chrXII	450628	C	T	Intergenic						

chrXII	491023	G	A	Intergenic						
chrXII	519868	G	A	Synonymous	643	3	YLR182W	1929	38	0
chrXII	551644	G	A	Missense	106	2	YLR203C	317	38	0
chrXII	576470	T	A	Upstream			YLR220W			
chrXII	584836	C	T	Missense	219	1	YLR223C	655	39	0
chrXII	607773	G	A	Synonymous	584	3	YLR233C	1752	37	0
chrXII	618732	C	T	Synonymous	400	3	YLR240W	1200	45	0
chrXII	642987	G	A	Missense	121	1	YLR253W	361	43	0
chrXII	643180	G	A	Missense	185	2	YLR253W	554	43	0
chrXII	644141	G	A	Synonymous	505	3	YLR253W	1515	44	0
chrXII	665763	G	A	Upstream			YLR260W			
chrXII	666000	C	T	Missense	53	1	YLR260W	157	38	0
chrXII	666863	G	A	Synonymous	340	3	YLR260W	1020	39	0
chrXII	723809	G	A	Upstream			YLR297W			
chrXII	725019	G	A	Missense	133	2	YLR298C	398	48	0
chrXII	735016	G	A	Downstream			YLR304C			
chrXII	741749	G	A	Synonymous	705	3	YLR305C	2115	39	0
chrXII	743928	G	A	Upstream			YLR306W			
chrXII	778628	C	T	Missense	109	1	YLR323C	325	46	0
chrXII	859795	G	A	Missense	82	1	YLR369W	244	43	0
chrXII	861300	G	A	Synonymous	583	3	YLR369W	1749	43	0
chrXII	875201	G	A	Upstream			YLR377C			
chrXII	898273	C	T	FivePrimeUTR			YLR388W			
chrXII	912885	G	A	Synonymous	670	1	YLR397C	2008	42	0
chrXII	918388	G	A	Missense	211	2	YLR398C	632	41	0
chrXII	950007	C	T	Downstream			YLR412C-A			
chrXII	960179	G	A	Synonymous	584	3	YLR419W	1752	42	1
chrXII	964775	G	A	Missense	331	1	YLR420W	991	45	0
chrXII	972855	C	T	Missense	106	1	YLR423C	316	45	0
chrXII	984982	G	A	Missense	697	1	YLR425W	2089	41	0
chrXII	989037	G	A	Missense	204	1	YLR427W	610	41	0
chrXII	994261	G	A	Synonymous	276	3	YLR430W	828	37	16

chrXII	1037964	G	A	Synonymous	624	3	YLR451W	1872	42	0
chrXII	1048370	G	A	Missense	1458	2	YLR454W	4373	36	4
chrXIII	13421	G	A	Upstream			YML130C			
chrXIII	23859	C	T	Upstream			YML124C			
chrXIII	30120	C	T	Upstream			YML119W			
chrXIII	31576	C	T	Synonymous	322	3	YML119W	966	43	0
chrXIII	36699	G	A	Missense	819	3	YML117W	2457	43	0
chrXIII	40400	C	T	Synonymous	465	3	YML115C	1395	44	0
chrXIII	74790	C	T	Missense	751	1	YML099C	2251		0
chrXIII	78135	C	T	Downstream			YML097C			
chrXIII	85625	G	A	Missense	846	1	YML093W	2536	29	1
chrXIII	86221	C	T	Synonymous	173	3	YML092C	519	46	0
chrXIII	88060	T	A	Missense	891	2	YML091C	2672	35	0
chrXIII	120348	C	T	Missense	326	2	YML074C	977	44	0
chrXIII	128726	A	G	Synonymous	214	3	YML072C	642	44	0
chrXIII	142800	G	A	Synonymous	197	3	YML065W	591	43	0
chrXIII	158330	C	T	Upstream			YML058W-A			
chrXIII	159377	C	T	Upstream			YML057W			
chrXIII	194239	C	T	Synonymous	484	3	YML042W	1452	43	0
chrXIII	202368	G	A	Intergenic						
chrXIII	244139	G	A	Upstream			YML013W			
chrXIII	244585	C	T	Missense	146	2	YML013W	437	41	0
chrXIII	254083	G	A	Missense	79	2	YML007W	236	40	0
chrXIII	254093	G	A	Synonymous	82	3	YML007W	246	40	0
chrXIII	265026	C	T	Synonymous	162	3	YML002W	486	41	2
chrXIII	267501	C	T	Missense	110	1	YML001W	328	44	0
chrXIII	271815	C	T	Upstream			YMR002W			
chrXIII	300201	G	A	Missense	445	2	YMR014W	1334	43	0
chrXIII	330347	G	A	Upstream			YMR030W			
chrXIII	336999	G	A	Missense	568	1	YMR032W	1702	39	0
chrXIII	342257	AGA	AA	Frameshift	421	2	YMR036C	1262	41	0

chrXIII	354008	G	A	Synonymous	46	3	YMR043W	138	47	0
chrXIII	366956	G	A	Upstream			YMR048W			
chrXIII	381062	G	A	Synonymous	613	1	YMR053C	1837	42	0
chrXIII	398197	G	A	Missense	99	1	YMR064W	295	44	0
chrXIII	436277	G	A	Upstream			YMR084W			
chrXIII	444092	G	A	Synonymous	337	3	YMR088C	1011	45	0
chrXIII	477737	ACC	AC	Upstream			YMR105W-A			
chrXIII	492198	G	A	Upstream			YMR110C			
chrXIII	494518	G	A	Upstream			YMR113W			
chrXIII	504958	G	A	Upstream			YMR119W			
chrXIII	541388	G	A	Missense	64	1	YMR136W	190	38	0
chrXIII	548888	G	A	Missense	392	2	YMR140W	1175	41	0
chrXIII	560748	G	A	Missense	128	2	YMR148W	383	43	0
chrXIII	569985	G	A	Missense	479	1	YMR155W	1435	44	0
chrXIII	576038	G	A	Missense	325	1	YMR160W	973	41	0
chrXIII	594730	G	A	Upstream			YMR167W			
chrXIII	606504	G	A	Missense	175	2	YMR172W	524	43	0
chrXIII	649704	G	A	Upstream			YMR193W			
chrXIII	657782	G	A	Missense	903	1	YMR196W	2707	41	0
chrXIII	674714	G	T	Missense	18	2	YMR205C	53	39	0
chrXIII	676111	T	A	Missense	72	3	YMR206W	216	44	0
chrXIII	702876	G	A	Missense	363	1	YMR217W	1087	40	0
chrXIII	707353	G	A	Missense	74	2	YMR219W	221	37	0
chrXIII	713630	G	A	Missense	439	1	YMR220W	1315	40	0
chrXIII	752029	C	T	Synonymous	23	3	YMR241W	69	43	0
chrXIII	758757	G	A	Synonymous	25	3	YMR244C-A	75	12	0
chrXIII	772691	C	T	Upstream			YMR251W			
chrXIII	774344	G	A	Upstream			YMR251W-A			
chrXIII	794988	G	A	Missense	24	1	YMR263W	70	42	0

chrXIII	799231	G	A	Synonymous	238	3	YMR266W	714	39	0
chrXIII	815755	G	A	Synonymous	943	1	YMR275C	2827	41	0
chrXIII	823783	G	A	Missense	341	1	YMR278W	1021	41	0
chrXIII	834427	G	A	Synonymous	224	3	YMR282C	672	40	0
chrXIII	849038	ATT	AT	Frameshift	118	3	YMR289W	354	41	0
chrXIII	893045	C	T	Synonymous	794	3	YMR309C	2382	43	0
chrXIII	893769	G	A	Missense	553	2	YMR309C	1658	44	0
chrXIV	21202	C	T	Missense	478	1	YNL329C	1432	42	0
chrXIV	34591	G	A	Upstream			YNL321W			
chrXIV	41986	G	A	Synonymous	456	3	YNL317W	1368	42	0
chrXIV	67415	A	T	Missense	300	2	YNL299W	899	43	0
chrXIV	76800	G	A	Upstream			YNL295W			
chrXIV	82918	G	A	Missense	38	2	YNL292W	113	42	0
chrXIV	86432	G	A	Missense	72	2	YNL290W	215	40	0
chrXIV	104634	G	A	Upstream			YNL284C			
chrXIV	113653	C	A	Missense	128	2	YNL278W	383	40	0
chrXIV	177570	G	A	Missense	721	1	YNL250W	2161	43	0
chrXIV	182855	G	A	Upstream			YNL247W			
chrXIV	184493	G	A	Missense	540	2	YNL247W	1619	36	0
chrXIV	187847	G	A	Upstream			YNL243W			
chrXIV	188794	G	A	Synonymous	248	3	YNL243W	744	37	0
chrXIV	210427	G	A	Missense	65	3	YNL234W	195	42	0
chrXIV	211427	G	A	Missense	399	1	YNL234W	1195	39	0
chrXIV	212760	G	A	Missense	280	2	YNL233W	839	37	0
chrXIV	215224	G	A	Missense	101	2	YNL232W	302	39	0
chrXIV	226750	C	T	Missense	117	2	YNL224C	350	40	0
chrXIV	228160	G	A	Missense	264	2	YNL223W	791	44	0
chrXIV	232206	G	A	Missense	497	2	YNL221C	1490	43	0
chrXIV	245436	G	A	Upstream			YNL214W			
chrXIV	271264	G	A	Upstream			YNL196C			
chrXIV	285847	G	A	Missense	530	1	YNL189W	1588	43	0
chrXIV	330170	C	T	Upstream			YNL162W-			

							A			
chrXIV	333585	G	A	Missense	330	2	YNL161W	989	44	0
chrXIV	341282	C	T	Synonymous	229	3	YNL156C	687	46	1
chrXIV	347923	G	A	Missense	200	2	YNL151C	599	43	0
chrXIV	358787	G	A	Synonymous	445	3	YNL142W	1335	42	0
chrXIV	370338	G	A	Upstream			YNL136W			
chrXIV	377777	G	A	Synonymous	819	3	YNL132W	2457	46	0
chrXIV	378354	G	A	Missense	1012	1	YNL132W	3034	42	0
chrXIV	408185	G	A	Upstream			YNL116W			
chrXIV	413567	C	T	Upstream			YNL112W			
chrXIV	428937	G	A	Synonymous	401	3	YNL103W	1203	37	1
chrXIV	432647	G	A	Missense	854	2	YNL102W	2561	40	0
chrXIV	434816	G	A	Upstream			YNL101W			
chrXIV	435047	G	A	Missense	17	1	YNL101W	49	40	0
chrXIV	450708	G	A	Upstream			YNL092W			
chrXIV	468445	C	T	Missense	439	1	YNL085W	1315	42	0
chrXIV	472462	G	A	Synonymous	362	3	YNL083W	1086	44	0
chrXIV	481601	G	A	Missense	71	1	YNL077W	211	39	0
chrXIV	491401	G	A	Upstream			YNL071W			
chrXIV	491904	G	A	Missense	128	1	YNL071W	382	44	0
chrXIV	504959	G	A	Synonymous	412	3	YNL065W	1236	37	0
chrXIV	514856	G	A	Missense	27	2	YNL059C	80	37	0
chrXIV	516416	C	T	Missense	5	2	YNL057W	14	13	0
chrXIV	517315	G	A	Missense	23	1	YNL056W	67	42	0
chrXIV	528168	G	A	Missense	695	1	YNL054W	2083	42	0
chrXIV	531616	G	A	Upstream			YNL052W			
chrXIV	543628	G	A	Synonymous	222	3	YNL045W	666	44	0
chrXIV	545281	G	A	Missense	5	2	YNL044W	14	21	0
chrXIV	552292	G	A	Upstream			YNL041C			
chrXIV	594107	G	A	Missense	294	2	YNL021W	881	42	0
chrXIV	605103	G	A	Upstream			YNL015W			
chrXIV	605916	G	A	Upstream			YNL014W			

chrXIV	612015	C	T	Missense	386	1	YNL011C	1156	43	0
chrXIV	612483	C	T	Missense	230	1	YNL011C	688	44	0
chrXIV	640527	G	A	Synonymous	44	3	YNR008W	132	42	0
chrXIV	649069	C	T	Missense	882	1	YNR013C	2644	37	0
chrXIV	685569	ACC	AC	Upstream			YNR032W			
chrXIV	692809	G	T	Missense	83	3	YNR034W-A	249	24	0
chrXIV	734923	G	A	Nonsense	211	3	YNR058W	633	42	0
chrXV	7813	G	A	Missense	547	1	YOL164W	1639	15	0
chrXV	39835	G	A	Upstream			YOL152W			
chrXV	40405	C	T	Upstream			YOL152W			
chrXV	69154	C	T	Upstream			YOL136C			
chrXV	80996	G	A	Missense	79	1	YOL127W	235	32	0
chrXV	101613	G	A	Missense	47	1	YOL115W	139	39	0
chrXV	104949	G	A	Synonymous	208	3	YOL113W	624	44	0
chrXV	109341	C	T	Missense	56	1	YOL110W	166	42	0
chrXV	150648	C	T	Missense	948	2	YOL089C	2843	40	0
chrXV	165099	G	A	Missense	915	2	YOL084W	2744	40	0
chrXV	166268	G	A	Synonymous	185	3	YOL083W	555	39	0
chrXV	176072	C	T	Missense	1668	2	YOL081W	5003	35	0
chrXV	177451	G	A	Missense	2128	1	YOL081W	6382	37	0
chrXV	229638	G	A	Missense	342	2	YOL054W	1025	42	0
chrXV	299306	G	A	Upstream			YOL014W			
chrXV	299731	G	A	Synonymous	13	3	YOL014W	39	42	16
chrXV	328386	G	A	Missense	519	1	YOR001W	1555	43	0
chrXV	330386	G	A	Missense	324	1	YOR002W	970	43	0
chrXV	360146	G	A	Missense	52	1	YOR015W	154	47	0
chrXV	384727	C	T	Missense	43	2	YOR029W	128	42	0
chrXV	401678	G	A	Missense	42	1	YOR037W	124	42	0
chrXV	408558	C	T	Missense	45	2	YOR042W	134	43	0
chrXV	433235	G	A	Synonymous	350	3	YOR057W	1050	39	0
chrXV	433323	G	A	Missense	380	1	YOR057W	1138	39	1

chrXV	448293	C	T	Synonymous	285	3	YOR065W	855	42	0
chrXV	449171	G	A	Upstream			YOR066W			
chrXV	452435	G	A	Missense	343	2	YOR067C	1028	42	0
chrXV	462051	C	T	Upstream			YOR071C			
chrXV	462412	G	A	Intergenic						
chrXV	468754	G	A	Synonymous	181	3	YOR075W	543	42	0
chrXV	474013	G	A	Missense	135	2	YOR079C	404	45	0
chrXV	475865	G	A	Synonymous	424	3	YOR080W	1272	41	0
chrXV	478135	C	T	Missense	352	1	YOR081C	1054	42	0
chrXV	481887	G	A	Upstream			YOR085W			
chrXV	508189	G	A	Missense	997	2	YOR098C	2990	42	0
chrXV	516141	G	A	Missense	300	1	YOR101W	898	44	0
chrXV	533524	G	A	Missense	673	1	YOR112W	2017	37	0
chrXV	552970	G	A	Upstream			YOR122C			
chrXV	566778	G	A	Upstream			YOR128C			
chrXV	572830	C	T	Synonymous	3	3	YOR131C	9	40	0
chrXV	585227	G	A	Synonymous	366	3	YOR138C	1098	42	0
chrXV	612358	G	A	Synonymous	120	3	YOR150W	360	43	0
chrXV	646475	G	A	Missense	637	2	YOR165W	1910	42	0
chrXV	655802	C	T	Synonymous	531	3	YOR172W	1593	-1	0
chrXV	662029	A	T	Upstream			YOR176W			
chrXV	665342	G	A	Intergenic						
chrXV	674023	G	A	Intergenic						
chrXV	674218	G	A	Downstream			YOR180C			
chrXV	702497	G	A	Intergenic						
chrXV	734929	G	A	Synonymous	335	3	YOR208W	1005	41	0
chrXV	744967	G	A	Synonymous	105	3	YOR213C	315	42	4
chrXV	746258	C	T	Missense	16	1	YOR214C	46	40	0
chrXV	748928	C	T	Missense	18	1	YOR216C	52	41	1
chrXV	749545	C	T	Missense	82	2	YOR217W	245	39	0
chrXV	764234	T	C	Synonymous	470	3	YOR227W	1410	42	0
chrXV	779515	G	A	Upstream			YOR235W			

chrXV	800111	C	T	Synonymous	207	3	YOR249C	621	42	0
chrXV	801881	G	A	Synonymous	143	1	YOR250C	427	44	0
chrXV	828142	C	T	Synonymous	811	3	YOR270C	2433	39	0
chrXV	856308	G	A	Synonymous	1317	3	YOR290C	3951	34	0
chrXV	859552	C	T	Missense	236	2	YOR290C	707	34	0
chrXV	860177	C	T	Missense	28	1	YOR290C	82	33	0
chrXV	865888	G	A	Missense	232	2	YOR292C	695	42	0
chrXV	887328	G	A	Missense	939	1	YOR304W	2815	36	0
chrXV	945498	G	A	Synonymous	301	3	YOR334W	903	41	0
chrXV	976486	C	T	Intergenic						
chrXV	994432	G	A	Missense	142	1	YOR350C	424	42	0
chrXV	1036934	G	A	Missense	34	2	YOR373W	101	29	0
chrXV	1038468	G	A	Synonymous	545	3	YOR373W	1635	32	0
chrXV	1069347	G	A	Downstream			YOR387C			
chrXVI	20485	C	T	Intergenic						
chrXVI	26526	C	T	Downstream			YPL272C			
chrXVI	37623	G	A	Synonymous	796	3	YPL268W	2388	43	0
chrXVI	52714	C	T	Upstream			YPL259C			
chrXVI	63999	C	T	Downstream			YPL257W			
chrXVI	114313	TAGA	TA	ExonJunction			YPL231W			
chrXVI	156876	C	T	Missense	240	1	YPL209C	718	47	0
chrXVI	183361	C	T	Upstream			YPL192C			
chrXVI	186691	C	T	Synonymous	345	3	YPL190C	1035	40	0
chrXVI	186972	C	T	Missense	252	1	YPL190C	754	40	0
chrXVI	188361	C	T	Synonymous	51	3	YPL189C-A	153	21	0
chrXVI	195285	C	T	Missense	48	1	YPL186C	142	44	4
chrXVI	221355	C	T	Synonymous	473	3	YPL174C	1419	43	0
chrXVI	242230	C	T	Synonymous	157	3	YPL163C	471	42	0
chrXVI	259801	C	T	Synonymous	377	3	YPL154C	1131	40	0
chrXVI	262379	C	T	Missense	605	2	YPL153C	1814	44	0
chrXVI	265083	C	T	Missense	19	2	YPL152W	56	44	0
chrXVI	284765	C	T	Missense	433	1	YPL141C	1297	42	0

chrXVI	290163	C	T	Missense	297	1	YPL139C	889	45	0
chrXVI	310593	C	T	Synonymous	128	3	YPL126W	384	42	0
chrXVI	318420	C	T	Missense	176	1	YPL123C	526	44	0
chrXVI	338971	C	T	Upstream			YPL111W			
chrXVI	412055	C	T	Upstream			YPL075W			
chrXVI	412634	C	T	Intron			YPL075W			
chrXVI	414199	G	A	Missense	399	1	YPL075W	1195	40	0
chrXVI	465859	C	T	Upstream			YPL047W			
chrXVI	501890	C	T	Synonymous	98	3	YPL026C	294	42	1
chrXVI	512353	C	T	Missense	609	2	YPL020C	1826	37	0
chrXVI	525461	C	T	Intergenic						
chrXVI	525985	T	A	Missense	300	2	YPL015C	899	38	0
chrXVI	536250	C	T	Missense	896	2	YPL009C	2687	39	0
chrXVI	540267	A	T	Missense	295	1	YPL008W	883	44	0
chrXVI	544939	G	A	Synonymous	103	3	YPL006W	309	36	0
chrXVI	569690	C	T	Synonymous	563	3	YPR007C	1689	39	0
chrXVI	592434	G	T	Synonymous	212	3	YPR016C	636	47	0
chrXVI	620106	C	T	Intergenic						
chrXVI	622583	C	T	Upstream			YPR028W			
chrXVI	627977	G	A	Missense	33	2	YPR030W	98	38	0
chrXVI	628736	G	A	Missense	286	2	YPR030W	857	41	0
chrXVI	638257	C	T	Missense	255	1	YPR033C	763	46	0
chrXVI	641293	C	T	Upstream			YPR035W			
chrXVI	641735	C	T	Upstream			YPR035W			
chrXVI	645593	C	T	Upstream			YPR036W-A			
chrXVI	673118	G	A	Synonymous	216	3	YPR057W	648	41	0
chrXVI	678193	C	T	Synonymous	14	3	YPR063C	42	30	0
chrXVI	679075	TCT	TT	Frameshift	42	2	YPR064W	125	43	0
chrXVI	712742	C	T	Missense	96	2	YPR088C	287	42	0
chrXVI	715029	C	T	Synonymous	585	3	YPR089W	1755	35	0
chrXVI	719903	C	T	Synonymous	174	3	YPR093C	522	44	0

chrXVI	741026	G	A	Synonymous	322	3	YPR106W	966	42	0
chrXVI	760368	C	T	Missense	115	2	YPR117W	344	39	0
chrXVI	775811	C	T	Upstream			YPR120C			
chrXVI	793288	C	T	Missense	201	2	YPR129W	602	44	0
chrXVI	802972	G	A	Missense	205	2	YPR137W	614	43	0
chrXVI	815015	G	A	Missense	209	1	YPR140W	625	47	0
chrXVI	843049	G	A	Upstream			YPR158W			
chrXVI	843888	G	A	Synonymous	209	3	YPR158W	627	45	0
chrXVI	844218	TAA	TA	Downstream			YPR158W			
chrXVI	870566	G	A	Upstream			YPR164W			
chrXVI	873643	G	A	Missense	981	1	YPR164W	2941	44	0
chrXVI	879755	G	A	Missense	356	1	YPR169W	1066	44	0
chrXVI	896094	G	A	Missense	45	2	YPR180W	134	43	0
chrXVI	903108	G	A	Synonymous	355	3	YPR184W	1065	38	2
chrXVI	903867	G	A	Synonymous	608	3	YPR184W	1824	38	0
chrXVI	914737	G	A	Missense	692	1	YPR189W	2074	42	0
chrXVI	918044	G	A	Missense	333	2	YPR190C	998	42	0

Supplementary Table S2

Variants between S288c and TDA3(4)									Natural variation at the genomic loci of the induced mutations in TDA3(4)	
CHROMOSOME	POSITION	S288c REFERENCE	INDUCED VARIANT	INFO	AMINO ACID	LOCATION IN CODON	GENE	NUCLEOTIDE IN ORF	Number of yeast strains for which the gene sequence was available on SGD	Number of yeast strains with a different nucleotide than the S288c reference
chrI	48908	G	A	Synonymous	115	3	YAL051W	345	41	0
chrI	49014	G	A	Missense	151	1	YAL051W	451	41	0
chrI	51925	G	A	Missense	224	2	YAL049C	671	46	0
chrI	69538	C	T	Upstream			YAL039C			
chrI	73859	C	T	Upstream			YAL037W			
chrI	75453	C	T	Missense	234	1	YAL036C	700	42	0
chrI	85599	G	A	Missense	478	2	YAL031C	1433	43	0
chrI	92627	G	A	Upstream			YAL028W			
chrI	95311	G	A	Missense	209	1	YAL027W	625	41	0
chrI	110470	C	T	Upstream			YAL022C			
chrI	117668	G	A	Missense	917	2	YAL019W	2750	40	0
chrI	120288	G	A	Missense	22	1	YAL017W	64	41	0
chrI	120590	G	A	Synonymous	122	3	YAL017W	366	41	0
chrI	124656	G	A	Upstream			YAL016W			
chrI	136414	G	A	Synonymous	187	3	YAL009W	561	42	0
chrI	148456	G	A	Missense	874	2	YAL001C	2621	28	0
chrI	171172	C	T	Missense	178	1	YAR018C	532	44	0
chrI	182310	C	T	Intergenic						

chrI	191074	G	A	Synonymous	294	3	YAR035W	882	32	0
chrI	191714	G	A	Missense	508	1	YAR035W	1522	32	0
chrI	192018	G	A	Missense	609	2	YAR035W	1826	31	0
chrI	193150	C	T	Missense	178	1	YAR042W	532	31	0
chrII	59350	G	A	Synonymous	11	3	YBL088C	33	39	0
chrII	62555	C	T	Synonymous	16	3	YBL086C	48	42	0
chrII	79066	G	A	Missense	1270	1	YBL079W	3808	40	0
chrII	79515	G	A	Synonymous	1419	3	YBL079W	4257	40	0
chrII	124623	T	C	Missense	46	2	YBL051C	137	36	0
chrII	124635	A	C	Missense	42	2	YBL051C	125	36	0
chrII	129895	C	T	Missense	716	1	YBL047C	2146	43	0
chrII	141279	G	A	Synonymous	11	3	YBL041W	33	43	0
chrII	149747	G	A	Missense	847	1	YBL037W	2539	42	0
chrII	166209	C	T	Missense	26	1	YBL029W	76	39	0
chrII	171359	G	A	Upstream			YBL025W			
chrII	173553	G	A	Synonymous	340	3	YBL024W	1020	43	0
chrII	195983	C	T	Downstream			YBL015W			
chrII	204810	G	A	Missense	425	1	YBL011W	1273	41	0
chrII	212553	G	A	Downstream			YBL007C			
chrII	213258	G	A	Missense	1037	1	YBL007C	3109	41	0
chrII	214708	G	A	Synonymous	553	3	YBL007C	1659	41	0
chrII	227747	G	A	Missense	38	1	YBL004W	112	37	0
chrII	228439	G	A	Synonymous	268	3	YBL004W	804	37	0
chrII	232353	G	A	Missense	1573	2	YBL004W	4718	44	0
chrII	238576	G	A	Intergenic						
chrII	249226	C	T	Synonymous	597	3	YBR007C	1791	36	1
chrII	287563	G	A	Synonymous	121	3	YBR023C	363	43	0
chrII	299059	G	A	Synonymous	256	3	YBR030W	768	44	0
chrII	302275	C	T	Missense	111	2	YBR033W	332	41	0
chrII	302998	C	T	Missense	352	2	YBR033W	1055	41	0
chrII	310141	G	A	Missense	58	2	YBR036C	173	43	0
chrII	320523	C	T	Missense	363	1	YBR042C	1087	44	0

chrII	320814	T	C	Missense	266	1	YBR042C	796	44	0
chrII	325525	C	T	Missense	179	1	YBR044C	535	44	0
chrII	331306	C	T	Missense	69	2	YBR046C	206	42	0
chrII	335850	G	A	Synonymous	323	3	YBR049C	969	42	2
chrII	370403	C	T	Synonymous	99	3	YBR066C	297	42	0
chrII	380065	C	T	Upstream			YBR071W			
chrII	383640	G	A	Missense	177	1	YBR073W	529	45	1
chrII	392751	C	T	Upstream			YBR078W			
chrII	401964	G	A	Synonymous	1096	3	YBR081C	3288	41	0
chrII	405929	G	A	Upstream			YBR081C			
chrII	422690	G	A	Missense	118	1	YBR086C	352	37	0
chrII	425877	G	A	Upstream			YBR088C			
chrII	438721	G	A	Missense	591	1	YBR097W	1771	42	0
chrII	445837	G	A	Missense	496	2	YBR102C	1487	42	0
chrII	474168	C	T	Upstream			YBR115C			
chrII	484656	C	T	Upstream			YBR122C			
chrII	532475	G	A	Upstream			YBR143C			
chrII	549434	G	A	Upstream			YBR155W			
chrII	549793	G	A	Missense	8	2	YBR155W	23	44	0
chrII	557746	C	T	Missense	400	1	YBR158W	1198	42	0
chrII	584414	G	A	Missense	232	2	YBR176W	695	42	0
chrII	595215	AAGA	AAA	Upstream			YBR183W			
chrII	598459	G	A	Missense	366	2	YBR184W	1097	43	0
chrII	600370	C	T	Upstream			YBR186W			
chrII	617088	C	T	Missense	479	2	YBR198C	1436	39	0
chrII	623244	C	T	Upstream			YBR201W			
chrII	673064	G	A	Missense	813	2	YBR225W	2438	41	0
chrII	677577	T	C	Missense	549	1	YBR229C	1645	39	1
chrII	680017	G	A	Upstream			YBR230W-A			
chrII	683579	G	A	Missense	51	2	YBR233W	152	43	0
chrII	692834	C	T	Missense	289	2	YBR237W	866	30	0

chrII	718310	C	T	Upstream			YBR250W			
chrII	751727	C	T	Missense	1794	1	YBR275C	5380	36	0
chrII	752726	G	A	Missense	1461	1	YBR275C	4381	40	0
chrII	758365	G	A	Missense	560	2	YBR276C	1679	43	0
chrII	774401	C	T	Upstream			YBR286W			
chrII	779377	G	A	Synonymous	29	3	YBR288C	87	42	0
chrII	786419	G	A	Upstream			YBR293W			
chrII	795933	G	A	Missense	1029	1	YBR295W	3085	39	0
chrII	796425	G	A	Missense	1193	1	YBR295W	3577	39	0
chrII	798136	G	A	Synonymous	129	3	YBR296C	387	44	0
chrIII	15592	C	T	Downstream			YCL064C			
chrIII	16098	C	T	Synonymous	261	3	YCL064C	783	42	0
chrIII	46496	G	A	Missense	137	2	YCL045C	410	41	0
chrIII	52692	G	A	Synonymous	16	3	YCL039W	48	44	1
chrIII	54474	G	A	Synonymous	610	3	YCL039W	1830	42	1
chrIII	66185	C	T	Missense	717	1	YCL030C	2149	44	0
chrIII	78597	G	A	Upstream			YCL024W			
chrIII	117955	C	T	Missense	132	1	YCR002C	394	42	0
chrIII	123145	G	A	Upstream			YCR006C			
chrIII	127854	G	A	Upstream			YCR008W			
chrIII	140790	C	T	Synonymous	48	3	YCR014C	144	38	0
chrIII	159836	C	T	Missense	180	1	YCR023C	538	41	0
chrIII	162878	C	T	Upstream			YCR024C-B			
chrIII	164422	C	T	Missense	640	1	YCR026C	1918	39	0
chrIII	176932	C	T	Upstream			YCR030C			
chrIII	193454	C	T	Missense	53	2	YCR036W	158	44	0
chrIII	193930	G	A	Missense	212	1	YCR036W	634	43	0
chrIII	203196	G	T	Synonymous	734	3	YCR042C	2202	38	0
chrIII	205428	C	T	Upstream			YCR042C			
chrIII	208296	C	T	Missense	439	1	YCR045C	1315	44	0
chrIII	210115	C	T	Synonymous	103	3	YCR046C	309	43	0
chrIII	225172	G	A	Upstream			YCR061W			

chrIII	233559	C	T	Missense	922	1	YCR067C	2764	40	0
chrIII	249479	C	T	Synonymous	189	3	YCR076C	567	41	0
chrIII	251451	C	T	Missense	393	2	YCR077C	1178	43	0
chrIII	252366	C	T	Missense	88	2	YCR077C	263	42	0
chrIII	254271	C	T	Upstream			YCR081W			
chrIV	28922	C	T	Upstream			YDL239C			
chrIV	38034	G	A	Missense	413	2	YDL233W	1238	40	0
chrIV	42396	C	T	Upstream			YDL230W			
chrIV	57801	C	T	Missense	869	1	YDL223C	2605	36	0
chrIV	88043	G	A	Upstream			YDL207W			
chrIV	97951	C	T	Missense	1	3	YDL203C	3	43	0
chrIV	122306	G	A	Missense	31	1	YDL189W	91	41	0
chrIV	127579	G	A	Missense	265	1	YDL185W	793	33	0
chrIV	127963	G	A	Missense	393	1	YDL185W	1177	33	0
chrIV	138673	G	A	Missense	128	2	YDL179W	383	44	1
chrIV	177835	C	T	Synonymous	355	1	YDL155W	1063	43	0
chrIV	188277	C	T	Synonymous	770	3	YDL148C	2310	41	0
chrIV	207903	C	T	Missense	887	1	YDL140C	2659	40	0
chrIV	237553	C	T	Missense	371	2	YDL126C	1112	47	0
chrIV	239989	C	T	Upstream			YDL124W			
chrIV	260431	C	T	Missense	506	2	YDL112W	1517	42	0
chrIV	281641	C	T	Missense	70	1	YDL101C	208	39	0
chrIV	296254	C	T	Missense	26	1	YDL090C	76	44	0
chrIV	330403	C	T	Synonymous	15	3	YDL072C	45	42	0
chrIV	338685	C	T	Missense	484	1	YDL063C	1450	39	0
chrIV	359960	C	T	Missense	442	3	YDL054C	1326	45	0
chrIV	373684	C	T	Upstream			YDL045C			
chrIV	379533	C	T	Missense	636	1	YDL040C	1906	41	0
chrIV	379929	C	T	Missense	504	1	YDL040C	1510	42	0
chrIV	394203	C	T	Upstream			YDL031W			
chrIV	395160	C	T	Missense	315	2	YDL031W	944	42	0
chrIV	399988	C	T	Missense	176	1	YDL029W	526	46	0

chrIV	417698	C	T	Missense	1273	1	YDL019C	3817	40	0
chrIV	418224	C	T	Synonymous	1097	3	YDL019C	3291	39	0
chrIV	421470	C	T	Synonymous	15	3	YDL019C	45	39	0
chrIV	438370	C	T	Synonymous	108	3	YDL007W	324	44	0
chrIV	447074	C	T	Missense	169	1	YDL002C	505	45	0
chrIV	485041	C	T	Missense	109	1	YDR019C	325	45	0
chrIV	488352	C	T	Missense	104	2	YDR022C	311	46	0
chrIV	499007	C	T	Missense	625	1	YDR028C	1873	41	0
chrIV	540883	C	T	Synonymous	107	3	YDR042C	321	35	0
chrIV	561226	C	T	Upstream			YDR052C			
chrIV	572549	C	T	Missense	634	2	YDR060W	1901	39	0
chrIV	609825	C	T	Missense	86	2	YDR081C	257	39	0
chrIV	620157	C	T	Upstream			YDR088C			
chrIV	620830	C	T	Intergenic						
chrIV	630867	C	T	Upstream			YDR093W			
chrIV	657117	C	T	Nonsense	117	3	YDR101C	351	40	0
chrIV	663338	C	T	Missense	525	1	YDR104C	1573	40	0
chrIV	684672	C	T	Synonymous	324	3	YDR117C	972	43	0
chrIV	689767	C	T	Missense	514	2	YDR119W	1541	39	0
chrIV	703845	C	T	Synonymous	204	3	YDR126W	612	47	0
chrIV	719545	C	T	Synonymous	253	3	YDR132C	759	44	0
chrIV	734046	G	A	Upstream			YDR140W			
chrIV	735466	C	T	Missense	1511	2	YDR141C	4532	42	3
chrIV	745976	C	T	Synonymous	42	3	YDR144C	126	41	0
chrIV	749865	C	T	Missense	293	2	YDR146C	878	38	0
chrIV	766204	C	T	Missense	167	1	YDR152W	499	44	0
chrIV	767380	C	T	Missense	198	1	YDR153C	592	45	0
chrIV	808034	C	T	Upstream			YDR172W			
chrIV	811953	C	T	Upstream			YDR174W			
chrIV	811978	C	T	Upstream			YDR174W			
chrIV	812407	G	A	Missense	100	1	YDR174W	298	47	0
chrIV	819166	G	A	Synonymous	11	1	YDR179C	31	45	0

chrIV	820021	C	T	Synonymous	197	1	YDR179W-A	589	41	0
chrIV	829554	C	T	Upstream			YDR183W			
chrIV	862831	G	A	Missense	260	1	YDR206W	778	36	0
chrIV	869053	C	T	Missense	277	2	YDR208W	830	45	0
chrIV	896014	C	T	Missense	327	2	YDR216W	980	42	0
chrIV	913720	G	A	Downstream			YDR223W			
chrIV	919836	G	A	Missense	756	1	YDR227W	2266	42	3
chrIV	932179	G	A	Missense	351	1	YDR234W	1051	43	0
chrIV	933175	G	A	Missense	683	1	YDR234W	2047	41	0
chrIV	967923	C	T	Upstream			YDR255C			
chrIV	1002935	C	T	Missense	190	1	YDR267C	568	44	0
chrIV	1018819	G	A	Upstream			YDR279W			
chrIV	1020684	C	T	Upstream			YDR280W			
chrIV	1033824	C	T	Synonymous	463	3	YDR285W	1389	40	0
chrIV	1035201	G	A	Upstream			YDR287W			
chrIV	1053574	C	T	Synonymous	358	3	YDR295C	1074	45	0
chrIV	1093979	G	A	Missense	73	1	YDR316W	217	44	0
chrIV	1123666	G	A	Synonymous	420	3	YDR326C	1260	40	0
chrIV	1139492	C	T	Synonymous	1187	3	YDR334W	3561	40	0
chrIV	1146820	C	T	Missense	168	1	YDR337W	502	43	0
chrIV	1165485	C	T	Upstream			YDR345C			
chrIV	1204111	C	T	Missense	34	1	YDR364C	100	38	0
chrIV	1233558	G	A	Upstream			YDR380W			
chrIV	1251060	G	A	Missense	292	2	YDR388W	875	42	0
chrIV	1253902	C	T	Missense	456	1	YDR389W	1366	43	0
chrIV	1254217	C	T	Missense	561	1	YDR389W	1681	43	0
chrIV	1255033	C	T	Synonymous	605	3	YDR390C	1815	40	0
chrIV	1263067	C	T	Upstream			YDR395W			
chrIV	1265277	G	A	Missense	652	1	YDR395W	1954	44	0
chrIV	1287021	C	T	Synonymous	306	3	YDR407C	918	39	0
chrIV	1292424	G	A	Missense	223	2	YDR410C	668	36	0

chrIV	1299116	G	A	Missense	229	1	YDR416W	685	39	0
chrIV	1315987	C	T	Missense	596	2	YDR422C	1787	42	0
chrIV	1326751	G	A	Synonymous	574	1	YDR430C	1720	40	0
chrIV	1327102	C	T	Missense	457	1	YDR430C	1369	38	0
chrIV	1354029	C	T	Missense	102	2	YDR446W	305	42	0
chrIV	1362546	C	T	Upstream			YDR452W			
chrIV	1362668	C	T	Upstream			YDR452W			
chrIV	1379933	C	T	Downstream			YDR458C			
chrIV	1410350	G	A	Upstream			YDR475C			
chrIV	1428639	C	T	Synonymous	114	3	YDR487C	342	44	0
chrIV	1429061	C	T	Upstream			YDR487C			
chrIV	1432515	C	T	Missense	585	2	YDR490C	1754	40	0
chrIV	1436163	C	T	Upstream			YDR494W			
chrIV	1447248	C	T	Upstream			YDR499W			
chrIV	1459009	C	T	Missense	71	2	YDR505C	212	42	0
chrIV	1464356	C	T	Synonymous	477	3	YDR507C	1431	39	0
chrV	9190	C	T	Intergenic						
chrV	25838	G	A	Synonymous	47	3	YEL068C	141	44	0
chrV	41342	C	T	Synonymous	204	3	YEL060C	612	42	1
chrV	56869	G	A	Missense	100	2	YEL052W	299	44	0
chrV	88287	G	A	Missense	451	1	YEL032W	1351	42	0
chrV	124300	C	T	Missense	215	2	YEL017W	644	47	0
chrV	125501	G	A	Missense	240	1	YEL016C	718	46	0
chrV	133613	C	T	Missense	165	2	YEL011W	494	41	0
chrV	141918	G	A	Synonymous	9	3	YEL007W	27	27	2
chrV	155210	G	A	Nonsense	564	2	YER001W	1691	39	0
chrV	159418	G	A	Upstream			YER004W			
chrV	184450	G	A	Upstream			YER015W			
chrV	188757	G	A	Missense	161	1	YER016W	481	39	0
chrV	192781	G	A	Upstream			YER019W			
chrV	192938	G	A	Missense	48	1	YER019W	142	42	0
chrV	210484	G	A	Upstream			YER027C			

chrV	223771	C	T	Missense	477	1	YER036C	1429	44	0
chrV	225258	G	A	Upstream			YER037W			
chrV	248295	C	T	Upstream			YER048C			
chrV	257520	C	T	Missense	147	1	YER052C	439	47	0
chrV	259434	C	T	Nonsense	69	2	YER053C	206	39	0
chrV	261574	C	T	Upstream			YER053C-A			
chrV	272028	G	A	Missense	87	3	YER058W	261	43	0
chrV	284049	C	T	Synonymous	58	3	YER064C	174	42	0
chrV	291295	C	T	Upstream			YER067W			
chrV	319112	G	A	Missense	65	1	YER079W	193	46	0
chrV	334003	G	A	Missense	396	1	YER088C	1186	41	5
chrV	334745	G	A	Synonymous	148	3	YER088C	444	41	0
chrV	342119	C	T	Missense	17	1	YER091C	49	44	0
chrV	369453	G	A	Missense	854	2	YER105C	2561	47	0
chrV	378504	C	T	Upstream			YER109C			
chrV	392203	G	A	Missense	504	1	YER114C	1510	44	0
chrV	394832	G	A	Synonymous	12	3	YER115C	36	45	0
chrV	396445	G	A	Upstream			YER117W			
chrV	419424	G	A	Missense	715	2	YER129W	2144	9	1
chrV	420233	G	A	Missense	985	1	YER129W	2953	9	0
chrV	438753	G	A	Upstream			YER136W			
chrV	491268	G	A	Upstream			YER158W-A			
chrV	507338	G	A	Synonymous	649	3	YER164W	1947	40	0
chrV	531177	C	T	Synonymous	1615	3	YER172C	4845	41	0
chrV	555296	G	A	Intergenic						
chrV	562538	C	T	Missense	30	1	YER186C	88	39	0
chrVI	37993	G	A	Synonymous	397	3	YFL049W	1191	45	0
chrVI	40381	C	T	Upstream			YFL047W			
chrVI	58963	G	A	Missense	61	2	YFL036W	182	41	0
chrVI	63939	C	T	Intron			YFL034C-B			
chrVI	65162	C	T	Upstream			YFL034W			

chrVI	75622	G	A	Synonymous	148	3	YFL031W	444	42	0
chrVI	78527	G	A	Missense	212	2	YFL029C	635	44	0
chrVI	81322	G	A	Synonymous	197	3	YFL027C	591	45	0
chrVI	84594	G	A	Missense	881	1	YFL025C	2641	38	0
chrVI	96297	G	A	Missense	111	2	YFL021W	332	36	0
chrVI	153118	C	T	Missense	3	1	YFR003C	7	42	0
chrVI	159702	G	A	Missense	135	2	YFR007W	404	44	0
chrVI	161427	C	T	Downstream			YFR008W			
chrVI	185596	G	A	Synonymous	365	3	YFR019W	1095	35	0
chrVI	190653	C	T	Missense	2051	2	YFR019W	6152	37	0
chrVI	195071	G	A	Missense	87	2	YFR021W	260	42	0
chrVI	198327	G	A	Missense	499	1	YFR022W	1495	45	0
chrVI	231909	G	A	Missense	844	1	YFR038W	2530	38	0
chrVI	237525	G	A	Missense	244	2	YFR041C	731	43	0
chrVI	240147	C	T	Missense	431	1	YFR044C	1291	42	0
chrVI	240758	C	T	Missense	227	2	YFR044C	680	43	0
chrVI	250872	G	A	Missense	311	2	YFR051C	932	44	0
chrVI	263903	G	A	Upstream			YFR055W			
chrVII	9549	C	T	Downstream			YGL258W-A			
chrVII	71147	G	A	Missense	493	1	YGL227W	1477	43	0
chrVII	114375	C	T	Missense	97	2	YGL203C	290	40	0
chrVII	132652	G	A	Synonymous	376	3	YGL195W	1128	35	0
chrVII	132812	G	A	Missense	430	1	YGL195W	1288	44	0
chrVII	158262	G	A	Synonymous	119	3	YGL181W	357	45	0
chrVII	163518	C	T	Missense	525	2	YGL179C	1574	42	0
chrVII	165838	C	T	Upstream			YGL179C			
chrVII	183032	C	T	Synonymous	215	1	YGL171W	643	38	0
chrVII	186996	G	A	Missense	313	2	YGL169W	938	42	0
chrVII	187747	C	T	Missense	908	1	YGL167C	2722	39	0
chrVII	189121	C	T	Missense	450	1	YGL167C	1348	42	0
chrVII	208026	C	T	Nonsense	332	1	YGL158W	994	36	0

chrVII	241863	C	T	Synonymous	1050	3	YGL140C	3150	35	0
chrVII	264869	C	T	Missense	331	1	YGL131C	991	39	0
chrVII	313021	T	A	Upstream			YGL100W			
chrVII	321640	CA	CAA	Upstream			YGL097W			
chrVII	327465	G	A	Intergenic						
chrVII	335194	A	C	Missense	103	3	YGL093W	309	44	0
chrVII	336217	G	A	Synonymous	444	3	YGL093W	1332	41	4
chrVII	340361	G	A	Missense	819	2	YGL092W	2456	42	0
chrVII	342619	C	T	Missense	142	1	YGL091C	424	36	0
chrVII	353345	C	T	Synonymous	96	3	YGL083W	288	43	0
chrVII	390790	G	A	Synonymous	242	3	YGL060W	726	46	0
chrVII	409540	G	A	Missense	22	2	YGL049C	65	42	0
chrVII	414477	G	A	Missense	126	1	YGL045W	376	41	0
chrVII	415848	GAA	GA	Downstream			YGL045W			
chrVII	433289	C	T	Synonymous	97	3	YGL034C	291	47	0
chrVII	435108	C	T	Upstream			YGL033W			
chrVII	465489	G	A	Upstream			YGL014W			
chrVII	467227	C	T	Synonymous	363	1	YGL014W	1087	39	0
chrVII	471965	G	A	Missense	112	1	YGL013C	334	38	0
chrVII	484527	G	A	Upstream			YGL006W-A			
chrVII	514501	G	A	Upstream			YGR013W			
chrVII	526746	G	A	Upstream			YGR021W			
chrVII	532662	G	A	Synonymous	8	3	YGR025W	24	45	0
chrVII	555628	G	A	Upstream			YGR034W			
chrVII	579709	G	A	Synonymous	78	3	YGR042W	234	43	0
chrVII	588087	C	T	Synonymous	461	3	YGR047C	1383	42	0
chrVII	589924	G	A	Missense	33	3	YGR048W	99	0	0
chrVII	597134	C	T	Missense	148	1	YGR054W	442	41	0
chrVII	609630	C	T	Upstream			YGR060W			
chrVII	664890	C	T	Missense	845	1	YGR090W	2533	41	5
chrVII	666401	G	A	Missense	21	1	YGR091W	61	40	0

chrVII	673912	G	A	Missense	576	2	YGR094W	1727	39	0
chrVII	688517	G	A	Missense	207	1	YGR099W	619	41	0
chrVII	691873	C	T	Synonymous	543	3	YGR100W	1629	41	0
chrVII	693961	G	A	Missense	200	2	YGR101W	599	45	0
chrVII	697871	C	T	Synonymous	166	3	YGR104C	498	48	0
chrVII	706768	G	A	Upstream			YGR109C			
chrVII	714975	G	A	Missense	423	1	YGR110W	1267	47	0
chrVII	718334	G	A	Missense	326	2	YGR112W	977	44	0
chrVII	720962	G	A	Missense	185	2	YGR116W	554	41	0
chrVII	774858	G	A	Upstream			YGR143W			
chrVII	780724	G	A	Missense	109	2	YGR144W	326	41	0
chrVII	807389	A	G	Missense	106	2	YGR160W	317	38	0
chrVII	808917	G	A	Synonymous	168	3	YGR161C	504	43	0
chrVII	824812	G	A	Missense	252	1	YGR162W	754	41	0
chrVII	833166	G	A	Downstream			YGR167W			
chrVII	869673	G	A	Missense	634	1	YGR186W	1900	45	0
chrVII	871986	G	A	Upstream			YGR187C			
chrVII	886414	G	A	Missense	488	1	YGR194C	1462	36	0
chrVII	893778	TACA	TA	Frameshift	120	3	YGR197C	360	39	0
chrVII	895101	G	A	Missense	137	1	YGR198W	409	45	0
chrVII	901844	C	T	Missense	143	1	YGR200C	427	38	0
chrVII	910033	G	A	Missense	274	2	YGR205W	821	40	0
chrVII	939464	C	T	Upstream			YGR222W			
chrVII	962674	G	A	Synonymous	48	3	YGR236C	144	22	0
chrVII	968842	C	T	Upstream			YGR238C			
chrVII	1004161	G	A	Upstream			YGR256W			
chrVII	1025546	C	T	Missense	64	1	YGR267C	190	44	0
chrVII	1028656	G	A	Synonymous	429	3	YGR270W	1287	42	1
chrVII	1032119	C	T	Missense	110	2	YGR271W	329	37	0
chrVII	1037470	G	A	Missense	1894	1	YGR271W	5680	42	0
chrVII	1046415	G	A	Synonymous	49	3	YGR277C	147	41	0
chrVII	1050271	G	A	Upstream			YGR279C			

chrVIII	25231	G	A	Synonymous	93	3	YHL038C	279	40	0
chrVIII	32594	C	T	Missense	55	1	YHL035C	163	30	0
chrVIII	54015	G	A	Missense	51	2	YHL026C	152	45	3
chrVIII	59325	G	A	Missense	1080	2	YHL023C	3239	41	0
chrVIII	76752	G	A	Missense	188	2	YHL014C	563	39	0
chrVIII	82644	G	A	Missense	360	1	YHL010C	1078	43	0
chrVIII	84432	C	T	Missense	210	2	YHL009C	629	44	0
chrVIII	112041	G	A	Synonymous	352	1	YHR004C	1054	45	0
chrVIII	128035	G	A	Missense	86	1	YHR011W	256	44	0
chrVIII	131489	G	A	Upstream			YHR014W			
chrVIII	134407	G	A	Upstream			YHR015W			
chrVIII	145423	C	T	Synonymous	476	3	YHR020W	1428	40	0
chrVIII	157429	G	A	Missense	1922	1	YHR023W	5764	41	0
chrVIII	166165	G	A	Missense	424	1	YHR028C	1270	36	0
chrVIII	168099	G	A	Missense	155	1	YHR029C	463	38	0
chrVIII	177750	G	A	Missense	84	1	YHR034C	250	42	0
chrVIII	185873	C	T	Missense	313	1	YHR039C	937	45	0
chrVIII	191677	C	T	Missense	379	1	YHR042W	1135	38	0
chrVIII	202533	G	A	Intergenic						
chrVIII	208627	G	A	Missense	325	2	YHR050W	974	44	0
chrVIII	236427	G	A	Missense	29	2	YHR070C-A	86	21	0
chrVIII	241747	G	A	Synonymous	28	3	YHR072W-A	84	21	0
chrVIII	247954	G	A	Missense	588	1	YHR074W	1762	45	0
chrVIII	256311	G	A	Upstream			YHR078W			
chrVIII	256934	G	A	Missense	192	2	YHR078W	575	44	0
chrVIII	272520	G	A	Upstream			YHR083W			
chrVIII	304203	G	A	Synonymous	481	3	YHR099W	1443	36	0
chrVIII	304914	G	A	Synonymous	718	3	YHR099W	2154	38	0
chrVIII	305539	G	A	Missense	927	1	YHR099W	2779	41	0
chrVIII	305786	G	A	Missense	1009	2	YHR099W	3026	39	0
chrVIII	314583	G	T	Missense	31	1	YHR100C	91	40	0

chrVIII	316405	C	A	Upstream			YHR102W			
chrVIII	317717	C	T	Synonymous	382	3	YHR102W	1146	40	0
chrVIII	337874	G	A	Upstream			YHR114W			
chrVIII	354325	G	A	Upstream			YHR123W			
chrVIII	387305	G	A	Missense	25	1	YHR143W-A	73	22	0
chrVIII	406145	G	A	Synonymous	1060	3	YHR154W	3180	44	0
chrVIII	416549	C	T	Missense	211	1	YHR158C	631	44	0
chrVIII	427583	G	A	Synonymous	532	3	YHR164C	1596	44	0
chrVIII	436840	G	A	Missense	65	2	YHR165W-A	194	39	0
chrVIII	459135	G	A	Upstream			YHR178W			
chrVIII	465181	G	A	Missense	2	1	YHR180W	4	42	0
chrVIII	474900	G	A	Synonymous	147	3	YHR185C	441	44	0
chrVIII	491490	G	A	Synonymous	248	3	YHR195W	744	47	0
chrVIII	503284	G	A	Synonymous	299	3	YHR202W	897	45	0
chrVIII	504279	C	T	Downstream			YHR202W			
chrVIII	507721	G	A	Missense	468	2	YHR204W	1403	42	0
chrIX	27276	C	T	Intergenic						
chrIX	27656	G	A	Intergenic						
chrIX	31951	G	A	Missense	206	1	YIL166C	616	40	0
chrIX	44246	G	A	Missense	808	1	YIL159W	2422	37	0
chrIX	44759	G	A	Missense	979	1	YIL159W	2935	38	0
chrIX	50990	G	A	Missense	967	2	YIL156W	2900	40	0
chrIX	72086	C	T	Synonymous	456	3	YIL147C	1368	40	0
chrIX	87374	G	A	Missense	670	2	YIL140W	2009	42	0
chrIX	107641	G	A	Missense	1866	2	YIL129C	5597	41	0
chrIX	121269	G	A	Missense	1093	2	YIL126W	3278	42	0
chrIX	121518	G	A	Missense	1176	2	YIL126W	3527	42	0
chrIX	126965	G	A	Synonymous	254	3	YIL124W	762	45	0
chrIX	129977	G	A	Upstream			YIL122W			
chrIX	137448	C	T	Missense	144	1	YIL119C	430	39	0

chrIX	138127	C	T	Upstream			YIL119C			
chrIX	140251	G	A	Missense	167	2	YIL118W	500	36	0
chrIX	140328	G	A	Missense	193	1	YIL118W	577	40	0
chrIX	148488	G	A	Synonymous	74	3	YIL115C	222	31	0
chrIX	171626	G	A	Upstream			YIL103W			
chrIX	174958	G	A	Upstream			YIL102C			
chrIX	194701	G	A	Synonymous	369	3	YIL090W	1107	42	0
chrIX	204617	G	A	Missense	13	1	YIL083C	37	42	0
chrIX	214268	G	A	Synonymous	590	3	YIL078W	1770	37	0
chrIX	214972	G	A	Downstream			YIL078W			
chrIX	228215	G	A	Synonymous	538	3	YIL072W	1614	39	0
chrIX	235342	C	T	Missense	45	1	YIL068C	133	38	0
chrIX	245295	G	A	Synonymous	89	1	YIL061C	265	41	0
chrIX	253226	G	A	Missense	234	1	YIL055C	700	44	0
chrIX	268793	G	A	Missense	48	2	YIL046W	143	40	0
chrIX	271091	G	A	Upstream			YIL045W			
chrIX	278901	G	A	Missense	159	2	YIL039W	476	39	0
chrIX	282785	G	A	Upstream			YIL038C			
chrIX	291789	G	A	Upstream			YIL031W			
chrIX	293279	T	A	Missense	216	2	YIL031W	647	36	0
chrIX	311452	G	A	Synonymous	96	3	YIL022W	288	34	0
chrIX	315753	G	A	Missense	221	1	YIL019W	661	34	0
chrIX	327497	G	A	Nonsense	465	3	YIL014W	1395	39	0
chrIX	333465	G	A	Upstream			YIL011W			
chrIX	346250	G	A	Missense	187	1	YIL005W	559	42	0
chrIX	366203	G	A	Missense	1236	1	YIR006C	3706	44	0
chrIX	373445	G	A	Missense	288	1	YIR008C	862	41	0
chrIX	382396	G	A	Upstream			YIR016W			
chrIX	395571	C	T	Intergenic						
chrIX	402074	G	A	Synonymous	766	3	YIR023W	2298	42	0
chrX	51959	G	A	Missense	398	2	YJL204C	1193	34	5
chrX	54073	G	A	Missense	73	2	YJL202C	218	20	0

chrX	54840	G	A	Synonymous	154	3	YJL201W	462	41	0
chrX	59367	G	A	Upstream			YJL200C			
chrX	82653	G	A	Synonymous	108	3	YJL185C	324	44	0
chrX	113662	G	A	Upstream			YJL163C			
chrX	113850	G	A	Upstream			YJL163C			
chrX	120167	G	A	Upstream			YJL159W			
chrX	130451	C	T	Missense	65	1	YJL155C	193	44	0
chrX	131251	G	A	Synonymous	895	3	YJL154C	2685	37	0
chrX	137519	C	T	Synonymous	47	3	YJL149W	141	41	0
chrX	149812	G	A	Synonymous	193	3	YJL141C	579	39	0
chrX	150456	C	T	Upstream			YJL140W			
chrX	167571	C	T	Synonymous	1599	3	YJL130C	4797	39	0
chrX	186876	G	A	Upstream			YJL125C			
chrX	186889	G	A	Upstream			YJL125C			
chrX	218044	C	T	Synonymous	271	3	YJL108C	813	45	0
chrX	218654	C	T	Missense	68	2	YJL108C	203	41	0
chrX	245980	G	A	Synonymous	130	3	YJL097W	390	46	0
chrX	256240	G	A	Synonymous	191	3	YJL093C	573	44	0
chrX	316107	G	A	Synonymous	123	3	YJL063C	369	44	0
chrX	330166	G	A	Upstream			YJL057C			
chrX	336633	C	T	Missense	246	1	YJL053W	736	39	0
chrX	344269	T	A	Missense	583	2	YJL050W	1748	43	0
chrX	354036	C	T	Intergenic						
chrX	403689	A	G	Missense	265	1	YJL019W	793	41	0
chrX	403700	G	A	Synonymous	268	3	YJL019W	804	42	0
chrX	405676	G	A	Missense	30	2	YJL016W	89	41	0
chrX	407374	G	A	Missense	18	2	YJL015C	53	39	0
chrX	420402	G	A	Missense	421	2	YJL008C	1262	40	0
chrX	434434	C	T	Missense	73	2	YJL002C	218	44	0
chrX	442752	G	A	Upstream			YJR003C			
chrX	451500	C	T	Missense	161	1	YJR007W	481	36	0
chrX	455208	G	A	Upstream			YJR009C			

chrX	458118	C	T	Missense	82	1	YJR010C-A	244	22	0
chrX	468691	C	T	Missense	270	2	YJR021C	809	44	0
chrX	490657	G	A	Synonymous	52	3	YJR031C	156	36	0
chrX	494347	G	A	Missense	702	2	YJR033C	2105	39	0
chrX	498368	G	A	Synonymous	338	3	YJR035W	1014	39	0
chrX	502528	G	A	Synonymous	288	3	YJR036C	864	42	0
chrX	504074	G	A	Missense	47	1	YJR039W	139	44	0
chrX	518878	C	T	Missense	103	2	YJR044C	308	40	0
chrX	534943	C	T	Missense	306	2	YJR053W	917	44	47
chrX	539354	G	A	Downstream			YJR055W			
chrX	548862	G	A	Missense	35	2	YJR060W	104	39	0
chrX	551990	C	T	Missense	494	1	YJR061W	1480	36	0
chrX	559139	G	A	Missense	7	2	YJR065C	20	45	0
chrX	582354	G	A	Upstream			YJR082C			
chrX	604163	C	T	Missense	14	2	YJR093C	41	41	0
chrX	626351	G	A	Missense	505	2	YJR106W	1514	39	0
chrX	635589	G	A	Missense	659	1	YJR110W	1975	36	0
chrX	639793	G	A	Upstream			YJR115W			
chrX	667695	G	A	Missense	18	1	YJR131W	52	42	0
chrX	684025	G	A	Upstream			YJR138W			
chrX	687593	G	A	Synonymous	1009	3	YJR138W	3027	42	0
chrX	720995	G	A	Missense	444	1	YJR152W	1330	41	0
chrXI	6713	C	T	Missense	203	1	YKL221W	607	39	0
chrXI	18193	C	T	Synonymous	49	3	YKL218C	147	42	0
chrXI	25126	C	T	Upstream			YKL216W			
chrXI	30378	C	T	Missense	104	1	YKL215C	310	46	0
chrXI	31203	C	T	Missense	164	2	YKL214C	491	44	0
chrXI	41088	G	A	Synonymous	642	3	YKL210W	1926	39	0
chrXI	53574	G	A	Upstream			YKL204W			
chrXI	78570	G	A	Missense	26	2	YKL194C	77	42	0
chrXI	98751	G	A	Missense	11	1	YKL183W	31	39	0
chrXI	104091	G	A	Missense	1141	1	YKL182W	3421	38	0

chrXI	111960	C	T	Missense	182	1	YKL179C	544	39	0
chrXI	121928	C	T	Nonsense	103	3	YKL174C	309	41	0
chrXI	124159	G	A	Missense	548	2	YKL173W	1643	38	0
chrXI	151229	G	A	Upstream			YKL161C			
chrXI	160331	C	T	Missense	338	1	YKL155C	1012	44	0
chrXI	178607	G	A	Synonymous	31	3	YKL142W	93	43	0
chrXI	200602	GAG	GT	Upstream			YKL129C			
chrXI	201421	C	T	Missense	117	3	YKL128C	351	42	0
chrXI	201569	G	A	Missense	68	2	YKL128C	203	44	0
chrXI	201605	A	G	Missense	56	2	YKL128C	167	41	0
chrXI	202265	G	A	Upstream			YKL128C			
chrXI	228607	G	A	Missense	65	1	YKL111C	193	39	0
chrXI	229126	G	A	Missense	252	2	YKL110C	755	42	0
chrXI	240336	G	A	Missense	750	1	YKL105C	2248	42	0
chrXI	264066	G	A	Missense	240	1	YKL094W	718	38	0
chrXI	275085	G	A	Upstream			YKL088W			
chrXI	279125	G	A	Missense	1	3	YKL085W	3	36	0
chrXI	280278	G	A	Upstream			YKL083W			
chrXI	291793	G	A	Missense	114	2	YKL077W	341	39	0
chrXI	294236	GAC	GA	Frameshift	24	2	YKL075C	71	42	0
chrXI	295783	G	A	Synonymous	137	3	YKL074C	411	42	0
chrXI	303473	G	A	Intergenic						
chrXI	322367	C	T	Upstream			YKL062W			
chrXI	346943	G	A	Upstream			YKL049C			
chrXI	356237	C	T	Upstream			YKL043W			
chrXI	376093	C	T	Missense	213	2	YKL033W	638	43	0
chrXI	376714	G	A	Missense	420	2	YKL033W	1259	44	0
chrXI	387024	G	A	Missense	423	1	YKL028W	1267	44	0
chrXI	391833	G	A	Synonymous	149	3	YKL025C	447	43	0
chrXI	397170	C	T	Missense	27	1	YKL022C	79	41	0
chrXI	423091	C	T	Missense	929	1	YKL010C	2785	41	0
chrXI	424052	C	T	Synonymous	608	3	YKL010C	1824	39	0

chrXI	425388	G	A	Missense	163	2	YKL010C	488	42	0
chrXI	439988	C	T	Upstream			YKL001C			
chrXI	445220	CTT	CT	Upstream			YKR003W			
chrXI	451568	GGAACACG	GG	Missense	45	3	YKR007W	135	44	0
chrXI	451568	GGAACACG	GG	Missense	45	3	YKR007W	135	44	0
chrXI	465672	G	A	Missense	134	1	YKR014C	400	41	0
chrXI	500266	G	A	Upstream			YKR030W			
chrXI	502558	G	A	Missense	1280	1	YKR031C	3838	42	0
chrXI	507290	G	A	Missense	131	3	YKR034W	393	41	0
chrXI	508626	C	T	Upstream			YKR035C			
chrXI	528818	C	T	Missense	335	2	YKR050W	1004	39	0
chrXI	530205	C	T	Synonymous	797	3	YKR050W	2391	41	0
chrXI	547382	C	T	Missense	182	1	YKR054C	544	14	0
chrXI	563591	C	T	Missense	349	1	YKR064W	1045	40	0
chrXI	568800	G	A	Missense	295	1	YKR067W	883	46	0
chrXI	609683	G	A	Synonymous	246	3	YKR090W	738	45	0
chrXI	611352	G	A	Upstream			YKR091W			
chrXI	612375	G	A	Downstream			YKR091W			
chrXI	614793	G	A	Upstream			YKR093W			
chrXI	630809	G	A	Upstream			YKR097W			
chrXI	645532	G	A	Upstream			YKR102W			
chrXI	650601	G	A	Intergenic						
chrXII	24263	G	A	Missense	232	2	YLL058W	695	41	0
chrXII	37861	C	T	Synonymous	537	3	YLL051C	1611	43	0
chrXII	63295	C	T	Nonsense	117	3	YLL040C	351	42	0
chrXII	69576	G	A	Missense	333	1	YLL035W	997	39	0
chrXII	90829	C	T	Missense	736	2	YLL026W	2207	38	0
chrXII	105282	G	A	Missense	1446	1	YLL021W	4336	40	0
chrXII	113416	C	T	Intergenic						
chrXII	114614	C	T	Intergenic						
chrXII	126775	C	T	Synonymous	414	3	YLL012W	1242	39	0
chrXII	137853	C	T	Missense	423	2	YLL006W	1268	42	0

chrXII	138058	C	T	Missense	830	2	YLL005C	2489	41	2
chrXII	153655	C	T	Missense	108	2	YLR001C	323	43	0
chrXII	161149	C	T	Synonymous	367	3	YLR005W	1101	45	0
chrXII	167481	C	T	Missense	108	1	YLR010C	322	41	0
chrXII	182381	C	T	Missense	342	2	YLR020C	1025	41	0
chrXII	185012	C	T	Upstream			YLR022C			
chrXII	187077	C	T	Missense	18	2	YLR023C	53	41	0
chrXII	187243	G	A	Upstream			YLR023C			
chrXII	191494	C	T	Synonymous	596	3	YLR024C	1788	39	0
chrXII	192843	C	T	Missense	147	1	YLR024C	439	41	0
chrXII	195500	C	T	Missense	325	1	YLR026C	973	40	0
chrXII	206284	G	A	Missense	432	1	YLR032W	1294	39	0
chrXII	223261	C	T	Upstream			YLR037C			
chrXII	225605	C	T	Missense	998	1	YLR039C	2992	45	0
chrXII	225862	C	T	Missense	912	2	YLR039C	2735	39	0
chrXII	227545	G	A	Missense	351	2	YLR039C	1052	43	0
chrXII	265682	G	A	Missense	76	2	YLR064W	227	42	0
chrXII	266034	C	T	Synonymous	193	3	YLR064W	579	46	0
chrXII	271778	C	T	Missense	713	2	YLR069C	2138	44	0
chrXII	318099	T	G	Downstream			YLR088W			
chrXII	326727	T	A	Missense	183	2	YLR093C	548	42	0
chrXII	337040	C	T	Missense	76	1	YLR097C	226	39	0
chrXII	353046	G	A	Missense	3565	1	YLR106C	10693	40	0
chrXII	353207	G	A	Missense	3511	2	YLR106C	10532	32	0
chrXII	354891	C	T	Missense	2950	1	YLR106C	8848	36	0
chrXII	362416	C	T	Synonymous	441	3	YLR106C	1323	30	0
chrXII	369819	C	T	Missense	94	1	YLR110C	280	44	0
chrXII	398721	G	A	Missense	33	2	YLR128W	98	41	0
chrXII	401056	C	T	Missense	467	2	YLR129W	1400	39	0
chrXII	406157	C	T	Synonymous	222	3	YLR131C	666	38	0
chrXII	416199	C	T	Missense	154	1	YLR136C	460	44	0
chrXII	519460	G	A	Synonymous	507	3	YLR182W	1521	42	0

chrXII	551628	C	T	Synonymous	111	3	YLR203C	333	38	0
chrXII	554153	C	T	Upstream			YLR206W			
chrXII	581493	C	T	Synonymous	93	3	YLR222C	279	41	0
chrXII	589123	C	T	Upstream			YLR226W			
chrXII	602042	C	T	Missense	141	2	YLR228C	422	45	0
chrXII	605053	CTT	CT	Upstream			YLR229C			
chrXII	617376	G	A	Upstream			YLR240W			
chrXII	624121	G	A	Upstream			YLR243W			
chrXII	649597	G	A	Missense	1061	3	YLR256W	3183	39	0
chrXII	668474	C	T	Missense	140	1	YLR262C	418	43	0
chrXII	679303	G	A	Synonymous	364	3	YLR267W	1092	42	0
chrXII	692800	G	A	Missense	416	1	YLR274W	1246	38	0
chrXII	698853	C	T	Missense	215	1	YLR277C	643	45	0
chrXII	698977	C	T	Synonymous	173	3	YLR277C	519	44	0
chrXII	719131	G	A	Missense	111	2	YLR291C	332	43	0
chrXII	725837	C	T	Upstream			YLR299W			
chrXII	725861	C	T	Upstream			YLR299W			
chrXII	753524	G	A	Missense	1157	2	YLR310C	3470	41	0
chrXII	759418	C	T	Upstream			YLR312W-A			
chrXII	769258	C	T	Downstream			YLR318W			
chrXII	827700	G	A	Synonymous	58	3	YLR348C	174	45	0
chrXII	834502	G	A	Missense	51	1	YLR353W	151	43	0
chrXII	857964	G	A	Missense	142	2	YLR368W	425	42	0
chrXII	878060	G	A	Upstream			YLR380W			
chrXII	883542	C	T	Missense	404	1	YLR382C	1210	38	4
chrXII	886653	G	T	Missense	456	1	YLR383W	1366	42	0
chrXII	888598	C	T	Missense	1104	2	YLR383W	3311	37	0
chrXII	907949	G	A	Upstream			YLR394W			
chrXII	911526	G	A	Missense	262	2	YLR396C	785	40	0
chrXII	915499	C	T	Missense	1174	2	YLR398C	3521	41	0
chrXII	915751	G	A	Missense	1090	2	YLR398C	3269	44	0

chrXII	915999	G	A	Synonymous	1007	3	YLR398C	3021	39	0
chrXII	916620	C	T	Synonymous	800	3	YLR398C	2400	41	0
chrXII	968720	G	A	Missense	942	1	YLR422W	2824	40	0
chrXII	969472	G	A	Synonymous	1192	3	YLR422W	3576	38	0
chrXII	1008617	G	A	Synonymous	877	1	YLR436C	2629	39	0
chrXII	1013043	G	A	Synonymous	181	3	YLR438W	543	43	0
chrXII	1014224	C	T	Upstream			YLR439W			
chrXII	1014224	C	T	Upstream			YLR439W			
chrXII	1021820	C	T	Synonymous	144	3	YLR442C	432	40	0
chrXII	1036160	G	A	Missense	23	2	YLR451W	68	41	0
chrXIII	16574	C	T	Missense	35	1	YML128C	103	41	0
chrXIII	26820	C	T	Upstream			YML121W			
chrXIII	49759	G	A	Downstream			YML111W			
chrXIII	60651	G	A	Missense	558	1	YML104C	1672	36	0
chrXIII	73269	G	A	Synonymous	882	3	YML100W	2646	40	1
chrXIII	74281	G	A	Missense	18	2	YML099W-A	53	23	0
chrXIII	77037	C	T	Missense	2	1	YML099C	4	41	0
chrXIII	85049	G	A	Missense	654	1	YML093W	1960	29	0
chrXIII	86559	G	A	Missense	61	1	YML092C	181	43	0
chrXIII	86559	G	A	Missense	61	1	YML092C	181	43	0
chrXIII	101737	C	T	Upstream			YML082W			
chrXIII	108825	G	A	Missense	7	2	YML080W	20	44	0
chrXIII	137849	G	A	Synonymous	100	3	YML068W	300	42	0
chrXIII	151964	G	A	Missense	32	1	YML060W	94	41	0
chrXIII	155002	C	T	Missense	1086	2	YML059C	3257	42	0
chrXIII	164392	G	A	Upstream			YML055W			
chrXIII	164392	G	A	Upstream			YML055W			
chrXIII	182064	G	A	Synonymous	197	3	YML046W	591	33	0
chrXIII	208466	C	T	Missense	132	2	YML035C	395	36	0
chrXIII	250324	A	G	Missense	883	2	YML010W	2648	40	0
chrXIII	255805	G	A	Downstream			YML007W			

chrXIII	268770	G	A	Downstream			YMR001C			
chrXIII	268877	G	A	Downstream			YMR001C			
chrXIII	273918	G	A	Upstream			YMR004W			
chrXIII	273918	G	A	Upstream			YMR004W			
chrXIII	283087	C	T	Upstream			YMR008C			
chrXIII	287877	C	T	Upstream			YMR011W			
chrXIII	307948	G	A	Missense	154	1	YMR017W	460	41	0
chrXIII	325688	C	T	Upstream			YMR027W			
chrXIII	336888	G	A	Missense	531	1	YMR032W	1591	39	1
chrXIII	340736	G	A	Upstream			YMR035W			
chrXIII	352773	G	A	Synonymous	57	3	YMR042W	171	46	0
chrXIII	369603	G	A	Synonymous	305	3	YMR049C	915	44	0
chrXIII	386967	G	A	Missense	19	1	YMR055C	55	41	0
chrXIII	392941	G	A	Missense	63	1	YMR061W	187	41	0
chrXIII	401723	G	A	Synonymous	61	3	YMR066W	183	39	0
chrXIII	406894	G	A	Synonymous	197	3	YMR068W	591	45	0
chrXIII	407646	G	A	Upstream			YMR069W			
chrXIII	409890	G	A	Missense	246	2	YMR070W	737	41	0
chrXIII	411660	G	A	Missense	31	2	YMR072W	92	44	0
chrXIII	412313	A	C	Nonsense	187	3	YMR073C	561	43	0
chrXIII	420105	G	A	Upstream			YMR076C			
chrXIII	424040	G	A	Missense	230	2	YMR078C	689	42	0
chrXIII	440954	G	A	Missense	583	1	YMR086W	1747	44	0
chrXIII	477779	G	A	Upstream			YMR105W-A			
chrXIII	480993	G	A	Upstream			YMR106C			
chrXIII	480993	G	A	Upstream			YMR106C			
chrXIII	497778	G	A	Nonsense	25	3	YMR115W	75	44	0
chrXIII	511030	C	T	Missense	16	1	YMR122C	46	42	0
chrXIII	519476	G	A	Missense	539	2	YMR125W	1616	43	0
chrXIII	523903	G	A	Missense	70	1	YMR128W	208	45	0
chrXIII	595720	G	A	Missense	279	1	YMR167W	835	39	0

chrXIII	616860	G	A	Missense	99	1	YMR177W	295	38	0
chrXIII	652808	G	A	Downstream			YMR194C-A			
chrXIII	655345	G	A	Synonymous	90	3	YMR196W	270	39	2
chrXIII	668927	G	A	Missense	146	1	YMR203W	436	44	0
chrXIII	677001	G	A	Downstream			YMR206W			
chrXIII	695213	C	T	Upstream			YMR214W			
chrXIII	708500	G	A	Synonymous	456	3	YMR219W	1368	37	0
chrXIII	708850	G	A	Missense	573	2	YMR219W	1718	40	0
chrXIII	713302	G	A	Synonymous	329	3	YMR220W	987	41	0
chrXIII	737517	G	A	Missense	198	1	YMR232W	592	39	0
chrXIII	741199	G	A	Missense	312	1	YMR234W	934	42	0
chrXIII	757087	G	A	Upstream			YMR244W			
chrXIII	778459	G	A	Synonymous	153	3	YMR255W	459	42	0
chrXIII	785360	G	A	Missense	1175	2	YMR259C	3524	39	0
chrXIII	800256	G	A	Nonsense	580	2	YMR266W	1739	44	0
chrXIII	823648	G	A	Missense	296	1	YMR278W	886	45	0
chrXIII	823757	C	T	Missense	332	2	YMR278W	995	39	0
chrXIII	831072	C	T	Synonymous	86	3	YMR280C	258	35	0
chrXIII	831164	G	A	Missense	56	1	YMR280C	166	42	0
chrXIII	834227	G	A	Missense	291	2	YMR282C	872	41	0
chrXIII	842289	G	A	Upstream			YMR285C			
chrXIII	843308	G	A	Missense	680	1	YMR287C	2038	42	0
chrXIII	843308	G	A	Missense	680	1	YMR287C	2038	42	0
chrXIII	845263	C	T	Missense	28	2	YMR287C	83	38	0
chrXIII	864218	G	A	Missense	134	1	YMR298W	400	45	0
chrXIII	866913	G	A	Missense	60	2	YMR300C	179	43	0
chrXIV	21369	G	A	Missense	422	2	YNL329C	1265	44	0
chrXIV	21826	G	A	Missense	270	1	YNL329C	808	39	0
chrXIV	56351	G	A	Upstream			YNL308C			
chrXIV	64807	G	A	Upstream			YNL300W			
chrXIV	85423	G	A	Synonymous	131	3	YNL291C	393	42	0

chrXIV	115700	G	A	Synonymous	810	3	YNL278W	2430	41	0
chrXIV	120489	G	A	Missense	408	1	YNL275W	1222	42	0
chrXIV	124435	G	A	Missense	518	2	YNL273W	1553	42	0
chrXIV	141161	G	A	Missense	95	2	YNL267W	284	41	0
chrXIV	170163	G	A	Missense	49	2	YNL253W	146	39	0
chrXIV	184357	G	A	Missense	495	1	YNL247W	1483	40	0
chrXIV	194064	C	T	Missense	914	2	YNL242W	2741	39	0
chrXIV	195058	G	A	Synonymous	1245	3	YNL242W	3735	34	3
chrXIV	195470	G	A	Missense	1383	1	YNL242W	4147	34	0
chrXIV	199112	G	A	Missense	289	2	YNL240C	866	42	0
chrXIV	245782	C	T	Missense	56	1	YNL214W	166	47	0
chrXIV	251261	G	A	Missense	111	1	YNL210W	331	46	0
chrXIV	256656	G	A	Downstream			YNL207W			
chrXIV	278380	G	A	Missense	627	1	YNL192W	1879	43	0
chrXIV	290409	G	A	Synonymous	304	3	YNL186W	912	41	0
chrXIV	310325	G	A	Upstream			YNL172W			
chrXIV	359633	G	A	Missense	13	2	YNL141W	38	45	0
chrXIV	370643	G	A	Synonymous	92	3	YNL136W	276	42	0
chrXIV	386669	G	A	Missense	895	1	YNL127W	2683	38	0
chrXIV	389566	C	T	Synonymous	780	3	YNL126W	2340	44	0
chrXIV	393390	G	A	Missense	167	1	YNL124W	499	42	0
chrXIV	412642	G	A	Upstream			YNL112W			
chrXIV	417881	T	C	Missense	203	2	YNL110C	608	41	0
chrXIV	431279	G	A	Missense	398	2	YNL102W	1193	42	0
chrXIV	435775	G	A	Synonymous	259	3	YNL101W	777	41	0
chrXIV	455918	G	A	Missense	1171	1	YNL091W	3511	39	0
chrXIV	456868	G	A	Missense	101	2	YNL089C	302	40	0
chrXIV	480930	G	A	Missense	388	2	YNL078W	1163	40	0
chrXIV	487639	G	A	Synonymous	162	3	YNL074C	486	43	0
chrXIV	504311	G	A	Synonymous	196	3	YNL065W	588	39	0
chrXIV	530920	G	A	Missense	327	1	YNL053W	979	42	0
chrXIV	543361	G	A	Synonymous	133	3	YNL045W	399	44	0

chrXIV	557466	G	A	Synonymous	149	3	YNL038W	447	44	0
chrXIV	581742	G	A	Upstream			YNL026W			
chrXIV	589786	G	A	Synonymous	459	1	YNL023C	1375	39	0
chrXIV	610189	C	T	Synonymous	140	3	YNL012W	420	21	0
chrXIV	627170	C	T	Upstream			YNL001W			
chrXIV	631784	G	A	Upstream			YNR001C			
chrXIV	631835	A	T	Upstream			YNR001C			
chrXIV	640891	G	A	Missense	166	1	YNR008W	496	42	0
chrXIV	665472	G	A	Missense	45	2	YNR019W	134	45	0
chrXIV	671273	G	A	Missense	286	1	YNR023W	856	39	0
chrXIV	672552	G	A	Synonymous	48	3	YNR024W	144	46	0
chrXIV	699924	G	A	Missense	79	1	YNR040W	235	40	0
chrXIV	706448	G	A	Missense	104	1	YNR045W	310	42	0
chrXIV	706478	G	A	Missense	114	1	YNR045W	340	44	0
chrXIV	719709	G	A	Synonymous	313	3	YNR052C	939	41	0
chrXIV	727109	G	A	Intergenic						
chrXIV	733843	G	A	Missense	76	2	YNR057C	227	46	0
chrXIV	736168	G	A	Upstream			YNR059W			
chrXIV	756213	C	T	Missense	963	1	YNR067C	2887	42	1
chrXIV	777792	G	A	Missense	316	2	YNR074C	947	45	0
chrXV	3357	G	A	Upstream			YOL164W-A			
chrXV	7733	G	A	Missense	520	2	YOL164W	1559	43	0
chrXV	12636	G	A	Upstream			YOL161C			
chrXV	33224	G	A	Intergenic						
chrXV	33230	G	A	Intergenic						
chrXV	50088	C	T	Missense	901	1	YOL145C	2701	44	0
chrXV	63333	C	T	Missense	673	2	YOL138C	2018	44	0
chrXV	66457	G	A	Synonymous	279	3	YOL137W	837	45	6
chrXV	66633	C	T	Missense	338	2	YOL137W	1013	43	0
chrXV	66681	G	A	Missense	354	2	YOL137W	1061	42	0
chrXV	77084	C	T	Upstream			YOL129W			

chrXV	102365	G	A	Synonymous	297	3	YOL115W	891	34	0
chrXV	116301	G	A	Upstream			YOL105C			
chrXV	134155	G	T	Missense	562	1	YOL098C	1684	40	0
chrXV	140369	C	T	Missense	327	1	YOL095C	979	42	0
chrXV	142022	G	A	Synonymous	178	3	YOL094C	534	46	0
chrXV	148411	C	T	Missense	344	1	YOL090W	1030	47	0
chrXV	149416	G	A	Missense	679	1	YOL090W	2035	44	0
chrXV	150352	G	C	Downstream			YOL090W			
chrXV	159188	G	A	Missense	6	1	YOL086W-A	16	22	0
chrXV	167072	C	T	Upstream			YOL083C-A			
chrXV	208153	C	T	Missense	705	1	YOL063C	2113	41	0
chrXV	214929	C	T	Synonymous	403	3	YOL060C	1209	39	5
chrXV	221018	G	A	Synonymous	84	3	YOL057W	252	40	0
chrXV	226442	G	A	Upstream			YOL055C			
chrXV	257004	G	A	Missense	87	2	YOL036W	260	38	0
chrXV	263217	G	A	Upstream			YOL033W			
chrXV	274311	C	T	Missense	15	1	YOL026C	43	46	0
chrXV	276292	G	A	Missense	446	1	YOL025W	1336	40	0
chrXV	277745	G	A	Upstream			YOL023W			
chrXV	278437	G	A	Missense	128	1	YOL023W	382	40	0
chrXV	281719	G	A	Upstream			YOL022C			
chrXV	317979	G	A	Missense	348	1	YOL004W	1042	39	5
chrXV	323587	C	T	Missense	260	1	YOL002C	778	45	0
chrXV	327047	G	A	Synonymous	72	3	YOR001W	216	42	0
chrXV	328699	C	T	Missense	623	2	YOR001W	1868	41	0
chrXV	330140	ATT	AT	Frameshift	242	1	YOR002W	724	37	0
chrXV	330140	ATT	AT	Frameshift	242	1	YOR002W	724	39	0
chrXV	332282	G	A	Synonymous	276	3	YOR003W	828	42	0
chrXV	345828	G	A	Downstream			YOR009W			
chrXV	370394	G	A	Downstream			YOR019W			
chrXV	380559	C	T	Missense	260	2	YOR026W	779	43	0

chrXV	382958	C	T	Downstream			YOR027W			
chrXV	383449	G	A	Downstream			YOR028C			
chrXV	433557	G	A	Downstream			YOR057W			
chrXV	433794	C	T	Missense	851	2	YOR058C	2552	41	0
chrXV	447518	G	A	Missense	27	2	YOR065W	80	45	0
chrXV	468754	G	A	Synonymous	181	3	YOR075W	543	42	0
chrXV	497190	C	T	Downstream			YOR092W			
chrXV	497190	C	T	Downstream			YOR092W			
chrXV	497316	G	A	Downstream			YOR093C			
chrXV	507960	G	A	Synonymous	1073	3	YOR098C	3219	39	0
chrXV	513969	G	A	Missense	104	1	YOR100C	310	41	0
chrXV	547442	G	A	Missense	196	1	YOR118W	586	43	0
chrXV	559867	G	A	Upstream			YOR125C			
chrXV	561985	G	A	Synonymous	272	3	YOR127W	816	43	1
chrXV	570251	C	T	Nonsense	186	2	YOR130C	557	43	0
chrXV	581072	G	A	Missense	275	1	YOR136W	823	43	0
chrXV	581286	A	G	Missense	346	2	YOR136W	1037	41	0
chrXV	582198	G	A	Missense	495	2	YOR137C	1484	42	0
chrXV	587044	G	A	Missense	22	1	YOR140W	64	38	0
chrXV	618060	G	A	Missense	77	1	YOR152C	229	41	0
chrXV	637434	C	T	Synonymous	375	3	YOR161C	1125	42	0
chrXV	640214	C	T	Synonymous	593	3	YOR162C	1779	44	0
chrXV	646709	G	A	Missense	715	2	YOR165W	2144	40	0
chrXV	660954	C	T	Missense	241	1	YOR175C	721	39	0
chrXV	662512	C	T	Missense	38	1	YOR176W	112	40	0
chrXV	662512	C	T	Missense	38	1	YOR176W	112	40	0
chrXV	667429	G	A	Upstream			YOR177C			
chrXV	674930	C	T	Missense	80	1	YOR180C	238	44	0
chrXV	689051	G	A	Missense	1095	3	YOR188W	3285		
chrXV	697972	C	T	Intergenic						
chrXV	717958	G	A	Synonymous	291	3	YOR197W	873	46	0
chrXV	750531	G	A	Missense	411	1	YOR217W	1231	45	0

chrXV	750861	C	T	Missense	521	1	YOR217W	1561	41	0
chrXV	752414	C	T	Missense	866	1	YOR219C	2596	47	0
chrXV	766927	C	T	Missense	284	2	YOR228C	851	44	0
chrXV	775519	G	A	Upstream			YOR233W			
chrXV	780252	C	T	Upstream			YOR236W			
chrXV	785712	G	A	Synonymous	285	3	YOR239W	855	19	0
chrXV	822891	G	A	Synonymous	659	3	YOR267C	1977	39	6
chrXV	823136	C	T	Missense	578	1	YOR267C	1732	38	0
chrXV	835667	C	T	Synonymous	255	3	YOR273C	765	40	0
chrXV	835667	C	T	Synonymous	255	3	YOR273C	765	40	0
chrXV	841185	C	T	Upstream			YOR276W			
chrXV	850730	G	A	Downstream			YOR286W			
chrXV	851933	C	T	Upstream			YOR287C			
chrXV	865112	C	T	Missense	1313	2	YOR291W	3938	41	0
chrXV	873804	G	A	Synonymous	1201	3	YOR296W	3603	35	7
chrXV	875138	C	T	Missense	62	1	YOR297C	184	37	0
chrXV	923194	C	T	Upstream			YOR323C			
chrXV	941329	C	T	Missense	686	2	YOR330C	2057	46	0
chrXV	954259	C	T	Upstream			YOR337W			
chrXV	955816	C	T	Synonymous	491	3	YOR337W	1473	45	0
chrXV	982791	C	T	Missense	322	1	YOR346W	964	46	0
chrXV	1000654	C	T	Missense	59	1	YOR353C	175	43	0
chrXV	1001929	C	T	Missense	433	1	YOR354C	1297	45	0
chrXV	1007216	G	A	Upstream			YOR356W			
chrXV	1013996	C	T	Missense	276	1	YOR360C	826	41	0
chrXV	1013996	C	T	Missense	276	1	YOR360C	826	43	0
chrXV	1028370	C	T	Missense	86	1	YOR369C	256	43	0
chrXV	1029736	G	A	Missense	420	2	YOR370C	1259	39	0
chrXV	1029858	G	A	Synonymous	379	3	YOR370C	1137	42	1
chrXV	1053579	C	T	Intergenic						
chrXVI	20619	C	T	Intergenic						
chrXVI	28525	G	A	Upstream			YPL272C			

chrXVI	34511	G	A	Missense	500	2	YPL269W	1499	43	0
chrXVI	36781	C	T	Synonymous	516	1	YPL268W	1546	45	6
chrXVI	76554	G	A	Upstream			YPL249C-A			
chrXVI	83514	G	A	Missense	228	2	YPL247C	683	40	0
chrXVI	103765	C	T	Synonymous	178	3	YPL235W	534	44	0
chrXVI	128957	G	A	Missense	61	2	YPL223C	182	46	0
chrXVI	154527	C	T	Synonymous	562	3	YPL210C	1686	38	0
chrXVI	159067	C	T	Missense	409	2	YPL208W	1226	40	0
chrXVI	185701	C	T	Synonymous	675	3	YPL190C	2025	38	0
chrXVI	192454	G	A	Missense	350	2	YPL188W	1049	40	0
chrXVI	222784	C	T	Upstream			YPL173W			
chrXVI	238903	G	A	Missense	59	1	YPL165C	175	23	0
chrXVI	239027	C	T	Synonymous	17	3	YPL165C	51	20	0
chrXVI	242566	C	T	Synonymous	45	3	YPL163C	135	42	0
chrXVI	250163	G	A	Synonymous	1058	3	YPL160W	3174	40	1
chrXVI	285091	G	A	Missense	324	2	YPL141C	971	41	0
chrXVI	296708	G	A	Upstream			YPL135W			
chrXVI	300290	C	T	Synonymous	313	3	YPL133C	939	44	0
chrXVI	313258	G	A	Upstream			YPL125W			
chrXVI	323057	C	T	Synonymous	329	3	YPL120W	987	46	0
chrXVI	331040	G	A	Missense	479	1	YPL116W	1435	46	0
chrXVI	360022	G	A	Missense	95	2	YPL102C	284	43	0
chrXVI	379777	TAA	TA	Frameshift	458	1	YPL089C	1372	41	0
chrXVI	390730	G	A	Missense	1222	1	YPL085W	3664	41	0
chrXVI	393252	G	A	Synonymous	2062	3	YPL085W	6186	30	0
chrXVI	406254	G	A	Missense	82	2	YPL080C	245	42	0
chrXVI	413917	G	A	Missense	305	1	YPL075W	913	39	0
chrXVI	432027	G	A	Missense	45	1	YPL062W	133	41	0
chrXVI	450798	G	A	Upstream			YPL058C			
chrXVI	477786	C	T	Missense	406	1	YPL040C	1216	37	0
chrXVI	485987	C	T	Upstream			YPL034W			
chrXVI	489953	G	A	Missense	471	2	YPL032C	1412	39	0

chrXVI	496707	G	A	Missense	401	2	YPL029W	1202	41	0
chrXVI	503210	C	T	Upstream			YPL024W			
chrXVI	514367	AGA	AA	Upstream			YPL020C			
chrXVI	530508	G	A	Synonymous	262	3	YPL012W	786	38	0
chrXVI	534575	G	A	Synonymous	43	3	YPL011C	129	43	0
chrXVI	552508	G	A	Synonymous	163	3	YPL003W	489	45	0
chrXVI	558864	C	T	Synonymous	160	3	YPR002W	480	44	0
chrXVI	562535	C	T	Missense	412	1	YPR003C	1234	43	0
chrXVI	567486	G	A	Missense	504	2	YPR006C	1511	37	0
chrXVI	602461	G	A	Synonymous	299	3	YPR021C	897	41	11
chrXVI	623980	C	T	Synonymous	107	3	YPR028W	321	21	0
chrXVI	632746	G	A	Missense	411	2	YPR031W	1232	37	0
chrXVI	644195	C	T	Synonymous	120	3	YPR036W	360	40	0
chrXVI	659639	G	A	Missense	153	2	YPR048W	458	44	0
chrXVI	663663	T	A	Synonymous	337	3	YPR049C	1011	44	11
chrXVI	695078	C	T	Missense	21	1	YPR074W-A	61	21	0
chrXVI	732935	G	A	Missense	196	2	YPR103W	587	40	0
chrXVI	740758	G	A	Missense	233	2	YPR106W	698	41	0
chrXVI	744164	A	T	Upstream			YPR109W			
chrXVI	757526	G	A	Missense	884	1	YPR115W	2650	39	0
chrXVI	774255	C	T	Missense	310	1	YPR120C	928	42	0
chrXVI	777600	C	T	Upstream			YPR121W			
chrXVI	782358	G	A	Missense	105	2	YPR122W	314	41	0
chrXVI	782879	G	A	Missense	279	1	YPR122W	835	42	0
chrXVI	794148	G	A	Synonymous	117	3	YPR131C	351	44	0
chrXVI	819435	G	A	Upstream			YPR142C			
chrXVI	840101	G	A	Upstream			YPR156C			
chrXVI	891920	C	T	Synonymous	53	3	YPR176C	159	45	1
chrXVI	908569	C	T	Missense	451	2	YPR185W	1352	46	0
chrXVI	911953	C	T	Upstream			YPR189W			
chrXVI	915280	G	A	Missense	873	1	YPR189W	2617	43	0

chrXVI	918342	C	T	Missense	234	1	YPR190C	700	39	0
chrXVI	921446	G	A	Upstream			YPR192W			
chrXVI	924091	G	A	Upstream			YPR193C			
chrXVI	927491	G	A	Upstream			YPR194C			

Supplementary Table S3

	Ethyl Acetate (mg/L)				
SEGREGANT OF TDA1(4)/ER7A	1st fermentation	2nd fermentation	3rd fermentation	Average	Comment
154	9.9	10.7		10.3	segregant unable to ferment to dryness
118	10.0	10.7	12.5	11.1	selected pool (< 20 mg/L ethyl acetate)
363	10.6	12.6	11.7	11.6	selected pool (< 20 mg/L ethyl acetate)
107	12.4	12.7	12.2	12.4	selected pool (< 20 mg/L ethyl acetate)
294	13.3	10.2	11.9	11.8	selected pool (< 20 mg/L ethyl acetate)
306	13.1	14.2	13.6	13.6	selected pool (< 20 mg/L ethyl acetate)
353	12.2	14.4	13.0	13.2	selected pool (< 20 mg/L ethyl acetate)
235	13.7	14.6	12.6	13.6	selected pool (< 20 mg/L ethyl acetate)
14	14.7	12.2	13.3	13.4	selected pool (< 20 mg/L ethyl acetate)
345	14.7	14.6	14.6	14.6	selected pool (< 20 mg/L ethyl acetate)
65	14.9	14.4	13.2	14.2	selected pool (< 20 mg/L ethyl acetate)
248	12.8	15.0	14.4	14.0	selected pool (< 20 mg/L ethyl acetate)
28	15.3	13.3	14.1	14.2	selected pool (< 20 mg/L ethyl acetate)
79	12.7	13.1	15.5	13.7	selected pool (< 20 mg/L ethyl acetate)
323	10.8	15.5	14.9	13.7	selected pool (< 20 mg/L ethyl acetate)
146	15.0	14.3	15.5	14.9	selected pool (< 20 mg/L ethyl acetate)
22	15.5	12.3	15.3	14.4	selected pool (< 20 mg/L ethyl acetate)
101	13.2	15.6	12.1	13.6	selected pool (< 20 mg/L ethyl acetate)
277	13.0	15.6	12.7	13.8	selected pool (< 20 mg/L ethyl acetate)
160	14.6	15.2	15.7	15.1	selected pool (< 20 mg/L ethyl acetate)
112	13.2	12.4	15.7	13.8	selected pool (< 20 mg/L ethyl acetate)
185	16.9	15.2	15.3	15.8	selected pool (< 20 mg/L ethyl acetate)
375	15.8	14.1	15.5	15.1	selected pool (< 20 mg/L ethyl acetate)
29	15.9	15.8	15.0	15.6	selected pool (< 20 mg/L ethyl acetate)
194	16.2	14.5	15.5	15.4	selected pool (< 20 mg/L ethyl acetate)
245	16.9	16.6	16.9	16.8	selected pool (< 20 mg/L ethyl acetate)

39	17.4	17.1	16.7	17.1	selected pool (< 20 mg/L ethyl acetate)
385	13.7	17.4	16.7	15.9	selected pool (< 20 mg/L ethyl acetate)
372	17.3	15.6	17.5	16.8	selected pool (< 20 mg/L ethyl acetate)
384	16.7	17.6	16.1	16.8	selected pool (< 20 mg/L ethyl acetate)
348	17.7	17.2	13.5	16.1	selected pool (< 20 mg/L ethyl acetate)
123	17.6	17.7	17.1	17.5	selected pool (< 20 mg/L ethyl acetate)
57	14.2	14.7	17.8	15.6	selected pool (< 20 mg/L ethyl acetate)
193	16.3	16.9	17.9	17.0	selected pool (< 20 mg/L ethyl acetate)
338	18.1	14.8	18.6	17.1	selected pool (< 20 mg/L ethyl acetate)
59	18.7	17.3	18.8	18.3	selected pool (< 20 mg/L ethyl acetate)
228	18.9	19.4	19.3	19.2	selected pool (< 20 mg/L ethyl acetate)
219	18.3	19.6	19.0	19.0	selected pool (< 20 mg/L ethyl acetate)
25	19.8	18.8	18.2	18.9	selected pool (< 20 mg/L ethyl acetate)
208	18.1	19.8	17.4	18.4	selected pool (< 20 mg/L ethyl acetate)
234	19.8	18.6	19.9	19.4	selected pool (< 20 mg/L ethyl acetate)
386	17.7	19.0	19.9	18.9	selected pool (< 20 mg/L ethyl acetate)
104	20.0	19.7		19.9	at least one fermentation with > 20 mg/L ethyl acetate
325	19.0	15.0	20.1	18.0	at least one fermentation with > 20 mg/L ethyl acetate
269	18.3	20.1		19.2	at least one fermentation with > 20 mg/L ethyl acetate
108	18.8	20.1		19.4	at least one fermentation with > 20 mg/L ethyl acetate
41	20.1			20.1	at least one fermentation with > 20 mg/L ethyl acetate
231	20.2			20.2	at least one fermentation with > 20 mg/L ethyl acetate
127	20.2			20.2	at least one fermentation with > 20 mg/L ethyl acetate
149	18.8	20.3		19.5	at least one fermentation with > 20 mg/L ethyl acetate
4	20.3			20.3	at least one fermentation with > 20 mg/L ethyl acetate
265	20.3			20.3	at least one fermentation with > 20 mg/L ethyl acetate
256	19.0	20.4		19.7	at least one fermentation with > 20 mg/L ethyl acetate
213	20.4			20.4	at least one fermentation with > 20 mg/L ethyl acetate
371	20.4			20.4	at least one fermentation with > 20 mg/L ethyl acetate
369	19.1	19.0	20.5	19.5	at least one fermentation with > 20 mg/L ethyl acetate
253	11.9	20.5		16.2	at least one fermentation with > 20 mg/L ethyl acetate
47	18.8	20.5		19.6	at least one fermentation with > 20 mg/L ethyl acetate

131	20.5			20.5	at least one fermentation with > 20 mg/L ethyl acetate
182	20.5			20.5	at least one fermentation with > 20 mg/L ethyl acetate
136	20.5			20.5	at least one fermentation with > 20 mg/L ethyl acetate
119	20.5			20.5	at least one fermentation with > 20 mg/L ethyl acetate
201	20.5			20.5	at least one fermentation with > 20 mg/L ethyl acetate
8	20.6			20.6	at least one fermentation with > 20 mg/L ethyl acetate
145	20.6			20.6	at least one fermentation with > 20 mg/L ethyl acetate
335	19.6	20.6		20.1	at least one fermentation with > 20 mg/L ethyl acetate
282	16.0	20.7		18.4	at least one fermentation with > 20 mg/L ethyl acetate
232	20.8			20.8	at least one fermentation with > 20 mg/L ethyl acetate
174	19.7	20.8		20.2	at least one fermentation with > 20 mg/L ethyl acetate
195	15.9	19.4	20.8	18.7	at least one fermentation with > 20 mg/L ethyl acetate
181	20.9			20.9	at least one fermentation with > 20 mg/L ethyl acetate
170	20.9			20.9	at least one fermentation with > 20 mg/L ethyl acetate
319	19.7	18.7	21.0	19.8	at least one fermentation with > 20 mg/L ethyl acetate
128	21.0			21.0	at least one fermentation with > 20 mg/L ethyl acetate
75	21.1			21.1	at least one fermentation with > 20 mg/L ethyl acetate
142	21.2			21.2	at least one fermentation with > 20 mg/L ethyl acetate
60	21.3			21.3	at least one fermentation with > 20 mg/L ethyl acetate
241	21.3			21.3	at least one fermentation with > 20 mg/L ethyl acetate
31	18.4	21.4		19.9	at least one fermentation with > 20 mg/L ethyl acetate
320	19.5	17.2	21.4	19.4	at least one fermentation with > 20 mg/L ethyl acetate
183	18.3	18.4	21.4	19.4	at least one fermentation with > 20 mg/L ethyl acetate
347	21.5			21.5	at least one fermentation with > 20 mg/L ethyl acetate
352	21.5			21.5	at least one fermentation with > 20 mg/L ethyl acetate
168	21.5			21.5	at least one fermentation with > 20 mg/L ethyl acetate
157	16.6	21.6		19.1	at least one fermentation with > 20 mg/L ethyl acetate
205	21.6			21.6	at least one fermentation with > 20 mg/L ethyl acetate
86	19.3	21.6		20.5	at least one fermentation with > 20 mg/L ethyl acetate
330	21.8			21.8	at least one fermentation with > 20 mg/L ethyl acetate
336	19.7	21.8		20.7	at least one fermentation with > 20 mg/L ethyl acetate
152	21.8			21.8	at least one fermentation with > 20 mg/L ethyl acetate

72	21.8			21.8	at least one fermentation with > 20 mg/L ethyl acetate
165	21.9			21.9	at least one fermentation with > 20 mg/L ethyl acetate
178	21.9			21.9	at least one fermentation with > 20 mg/L ethyl acetate
38	20.1	21.9		21.0	at least one fermentation with > 20 mg/L ethyl acetate
257	19.4	22.0		20.7	at least one fermentation with > 20 mg/L ethyl acetate
95	22.0			22.0	at least one fermentation with > 20 mg/L ethyl acetate
261	19.6	22.0		20.8	at least one fermentation with > 20 mg/L ethyl acetate
203	22.0			22.0	at least one fermentation with > 20 mg/L ethyl acetate
126	22.0			22.0	at least one fermentation with > 20 mg/L ethyl acetate
229	18.7	19.9	22.1	20.2	at least one fermentation with > 20 mg/L ethyl acetate
310	22.1			22.1	at least one fermentation with > 20 mg/L ethyl acetate
318	22.2			22.2	at least one fermentation with > 20 mg/L ethyl acetate
18	22.3			22.3	at least one fermentation with > 20 mg/L ethyl acetate
45	22.4			22.4	at least one fermentation with > 20 mg/L ethyl acetate
329	22.4			22.4	at least one fermentation with > 20 mg/L ethyl acetate
49	22.5			22.5	at least one fermentation with > 20 mg/L ethyl acetate
326	22.6			22.6	at least one fermentation with > 20 mg/L ethyl acetate
314	22.6			22.6	at least one fermentation with > 20 mg/L ethyl acetate
376	22.6			22.6	at least one fermentation with > 20 mg/L ethyl acetate
139	22.6			22.6	at least one fermentation with > 20 mg/L ethyl acetate
5	22.7			22.7	at least one fermentation with > 20 mg/L ethyl acetate
53	22.7			22.7	at least one fermentation with > 20 mg/L ethyl acetate
249	22.8			22.8	at least one fermentation with > 20 mg/L ethyl acetate
355	22.8			22.8	at least one fermentation with > 20 mg/L ethyl acetate
99	22.8			22.8	at least one fermentation with > 20 mg/L ethyl acetate
252	18.1	22.8		20.5	at least one fermentation with > 20 mg/L ethyl acetate
259	18.4	22.8		20.6	at least one fermentation with > 20 mg/L ethyl acetate
151	22.9			22.9	at least one fermentation with > 20 mg/L ethyl acetate
20	22.9			22.9	at least one fermentation with > 20 mg/L ethyl acetate
301	23.1			23.1	at least one fermentation with > 20 mg/L ethyl acetate
138	23.1			23.1	at least one fermentation with > 20 mg/L ethyl acetate
307	23.2			23.2	at least one fermentation with > 20 mg/L ethyl acetate

289	23.2			23.2	at least one fermentation with > 20 mg/L ethyl acetate
163	23.2			23.2	at least one fermentation with > 20 mg/L ethyl acetate
51	23.3			23.3	at least one fermentation with > 20 mg/L ethyl acetate
246	23.3			23.3	at least one fermentation with > 20 mg/L ethyl acetate
78	23.5			23.5	at least one fermentation with > 20 mg/L ethyl acetate
268	18.9	23.5		21.2	at least one fermentation with > 20 mg/L ethyl acetate
267	23.5			23.5	at least one fermentation with > 20 mg/L ethyl acetate
173	23.6			23.6	at least one fermentation with > 20 mg/L ethyl acetate
188	19.5	23.6		21.6	at least one fermentation with > 20 mg/L ethyl acetate
17	23.6			23.6	at least one fermentation with > 20 mg/L ethyl acetate
304	23.7			23.7	at least one fermentation with > 20 mg/L ethyl acetate
247	23.7			23.7	at least one fermentation with > 20 mg/L ethyl acetate
281	23.8			23.8	at least one fermentation with > 20 mg/L ethyl acetate
11	23.8			23.8	at least one fermentation with > 20 mg/L ethyl acetate
244	15.5	23.8		19.7	at least one fermentation with > 20 mg/L ethyl acetate
90	23.9			23.9	at least one fermentation with > 20 mg/L ethyl acetate
198	18.8	18.1	23.9	20.3	at least one fermentation with > 20 mg/L ethyl acetate
264	23.9			23.9	at least one fermentation with > 20 mg/L ethyl acetate
114	24.0			24.0	at least one fermentation with > 20 mg/L ethyl acetate
358	19.3	24.0		21.7	at least one fermentation with > 20 mg/L ethyl acetate
55	19.8	24.1		22.0	at least one fermentation with > 20 mg/L ethyl acetate
162	24.2			24.2	at least one fermentation with > 20 mg/L ethyl acetate
207	24.3			24.3	at least one fermentation with > 20 mg/L ethyl acetate
223	24.6			24.6	at least one fermentation with > 20 mg/L ethyl acetate
332	24.8			24.8	at least one fermentation with > 20 mg/L ethyl acetate
210	24.9			24.9	at least one fermentation with > 20 mg/L ethyl acetate
215	24.9			24.9	at least one fermentation with > 20 mg/L ethyl acetate
171	25.0			25.0	at least one fermentation with > 20 mg/L ethyl acetate
285	25.1			25.1	at least one fermentation with > 20 mg/L ethyl acetate
137	25.1			25.1	at least one fermentation with > 20 mg/L ethyl acetate
71	25.2			25.2	at least one fermentation with > 20 mg/L ethyl acetate
130	25.2			25.2	at least one fermentation with > 20 mg/L ethyl acetate

297	25.2			25.2	at least one fermentation with > 20 mg/L ethyl acetate
383	25.2			25.2	at least one fermentation with > 20 mg/L ethyl acetate
224	25.2			25.2	at least one fermentation with > 20 mg/L ethyl acetate
308	25.3			25.3	at least one fermentation with > 20 mg/L ethyl acetate
270	16.0	25.3		20.6	at least one fermentation with > 20 mg/L ethyl acetate
176	25.3			25.3	at least one fermentation with > 20 mg/L ethyl acetate
62	25.4			25.4	at least one fermentation with > 20 mg/L ethyl acetate
134	25.4			25.4	at least one fermentation with > 20 mg/L ethyl acetate
143	25.6			25.6	at least one fermentation with > 20 mg/L ethyl acetate
88	25.7			25.7	at least one fermentation with > 20 mg/L ethyl acetate
359	25.8			25.8	at least one fermentation with > 20 mg/L ethyl acetate
361	25.8			25.8	at least one fermentation with > 20 mg/L ethyl acetate
135	25.9			25.9	at least one fermentation with > 20 mg/L ethyl acetate
342	26.0			26.0	at least one fermentation with > 20 mg/L ethyl acetate
110	20.0	26.0		23.0	at least one fermentation with > 20 mg/L ethyl acetate
63	26.0			26.0	at least one fermentation with > 20 mg/L ethyl acetate
46	26.1			26.1	at least one fermentation with > 20 mg/L ethyl acetate
199	26.1			26.1	at least one fermentation with > 20 mg/L ethyl acetate
83	26.3			26.3	at least one fermentation with > 20 mg/L ethyl acetate
360	26.4			26.4	at least one fermentation with > 20 mg/L ethyl acetate
196	26.6			26.6	at least one fermentation with > 20 mg/L ethyl acetate
315	26.8			26.8	at least one fermentation with > 20 mg/L ethyl acetate
10	26.8			26.8	at least one fermentation with > 20 mg/L ethyl acetate
254	15.1	26.9		21.0	at least one fermentation with > 20 mg/L ethyl acetate
296	26.9			26.9	at least one fermentation with > 20 mg/L ethyl acetate
337	27.0			27.0	at least one fermentation with > 20 mg/L ethyl acetate
287	27.1			27.1	at least one fermentation with > 20 mg/L ethyl acetate
189	27.4			27.4	at least one fermentation with > 20 mg/L ethyl acetate
316	27.5			27.5	at least one fermentation with > 20 mg/L ethyl acetate
106	27.5			27.5	at least one fermentation with > 20 mg/L ethyl acetate
167	27.6			27.6	at least one fermentation with > 20 mg/L ethyl acetate
37	27.6			27.6	at least one fermentation with > 20 mg/L ethyl acetate

67	28.1			28.1	at least one fermentation with > 20 mg/L ethyl acetate
302	28.1			28.1	at least one fermentation with > 20 mg/L ethyl acetate
117	28.2			28.2	at least one fermentation with > 20 mg/L ethyl acetate
54	28.2			28.2	at least one fermentation with > 20 mg/L ethyl acetate
84	28.3			28.3	at least one fermentation with > 20 mg/L ethyl acetate
36	28.3			28.3	at least one fermentation with > 20 mg/L ethyl acetate
263	19.5	28.4		23.9	at least one fermentation with > 20 mg/L ethyl acetate
202	28.4			28.4	at least one fermentation with > 20 mg/L ethyl acetate
44	28.5			28.5	at least one fermentation with > 20 mg/L ethyl acetate
217	28.6			28.6	at least one fermentation with > 20 mg/L ethyl acetate
260	28.6			28.6	at least one fermentation with > 20 mg/L ethyl acetate
172	28.6			28.6	at least one fermentation with > 20 mg/L ethyl acetate
262	29.4			29.4	at least one fermentation with > 20 mg/L ethyl acetate
164	29.4			29.4	at least one fermentation with > 20 mg/L ethyl acetate
344	29.8			29.8	at least one fermentation with > 20 mg/L ethyl acetate
365	30.1			30.1	at least one fermentation with > 20 mg/L ethyl acetate
24	30.2			30.2	at least one fermentation with > 20 mg/L ethyl acetate
239	30.2			30.2	at least one fermentation with > 20 mg/L ethyl acetate
64	30.2			30.2	at least one fermentation with > 20 mg/L ethyl acetate
279	30.3			30.3	at least one fermentation with > 20 mg/L ethyl acetate
74	30.3			30.3	at least one fermentation with > 20 mg/L ethyl acetate
116	30.6			30.6	at least one fermentation with > 20 mg/L ethyl acetate
278	30.6			30.6	at least one fermentation with > 20 mg/L ethyl acetate
70	30.7			30.7	at least one fermentation with > 20 mg/L ethyl acetate
179	30.9			30.9	at least one fermentation with > 20 mg/L ethyl acetate
26	30.9			30.9	at least one fermentation with > 20 mg/L ethyl acetate
364	31.0			31.0	at least one fermentation with > 20 mg/L ethyl acetate
298	31.1			31.1	at least one fermentation with > 20 mg/L ethyl acetate
233	31.1			31.1	at least one fermentation with > 20 mg/L ethyl acetate
211	31.1			31.1	at least one fermentation with > 20 mg/L ethyl acetate
200	31.2			31.2	at least one fermentation with > 20 mg/L ethyl acetate
288	31.2			31.2	at least one fermentation with > 20 mg/L ethyl acetate

209	31.2			31.2	at least one fermentation with > 20 mg/L ethyl acetate
238	31.6			31.6	at least one fermentation with > 20 mg/L ethyl acetate
144	31.8			31.8	at least one fermentation with > 20 mg/L ethyl acetate
312	32.0			32.0	at least one fermentation with > 20 mg/L ethyl acetate
378	32.0			32.0	at least one fermentation with > 20 mg/L ethyl acetate
166	32.0			32.0	at least one fermentation with > 20 mg/L ethyl acetate
43	32.1			32.1	at least one fermentation with > 20 mg/L ethyl acetate
368	32.4			32.4	at least one fermentation with > 20 mg/L ethyl acetate
266	32.6			32.6	at least one fermentation with > 20 mg/L ethyl acetate
290	33.0			33.0	at least one fermentation with > 20 mg/L ethyl acetate
192	33.1			33.1	at least one fermentation with > 20 mg/L ethyl acetate
187	33.1			33.1	at least one fermentation with > 20 mg/L ethyl acetate
373	33.1			33.1	at least one fermentation with > 20 mg/L ethyl acetate
197	33.1			33.1	at least one fermentation with > 20 mg/L ethyl acetate
50	33.3			33.3	at least one fermentation with > 20 mg/L ethyl acetate
346	33.3			33.3	at least one fermentation with > 20 mg/L ethyl acetate
243	33.5			33.5	at least one fermentation with > 20 mg/L ethyl acetate
42	33.5			33.5	at least one fermentation with > 20 mg/L ethyl acetate
2	33.6			33.6	at least one fermentation with > 20 mg/L ethyl acetate
370	33.7			33.7	at least one fermentation with > 20 mg/L ethyl acetate
33	33.7			33.7	at least one fermentation with > 20 mg/L ethyl acetate
96	33.9			33.9	at least one fermentation with > 20 mg/L ethyl acetate
206	33.9			33.9	at least one fermentation with > 20 mg/L ethyl acetate
250	33.9			33.9	at least one fermentation with > 20 mg/L ethyl acetate
225	34.0			34.0	at least one fermentation with > 20 mg/L ethyl acetate
309	34.1			34.1	at least one fermentation with > 20 mg/L ethyl acetate
357	34.2			34.2	at least one fermentation with > 20 mg/L ethyl acetate
13	34.2			34.2	at least one fermentation with > 20 mg/L ethyl acetate
240	34.2			34.2	at least one fermentation with > 20 mg/L ethyl acetate
293	34.5			34.5	at least one fermentation with > 20 mg/L ethyl acetate
356	34.6			34.6	at least one fermentation with > 20 mg/L ethyl acetate
218	34.6			34.6	at least one fermentation with > 20 mg/L ethyl acetate

158	34.7			34.7	at least one fermentation with > 20 mg/L ethyl acetate
381	34.8			34.8	at least one fermentation with > 20 mg/L ethyl acetate
113	35.2			35.2	at least one fermentation with > 20 mg/L ethyl acetate
7	35.5			35.5	at least one fermentation with > 20 mg/L ethyl acetate
190	35.6			35.6	at least one fermentation with > 20 mg/L ethyl acetate
322	35.7			35.7	at least one fermentation with > 20 mg/L ethyl acetate
351	36.0			36.0	at least one fermentation with > 20 mg/L ethyl acetate
186	36.1			36.1	at least one fermentation with > 20 mg/L ethyl acetate
15	36.1			36.1	at least one fermentation with > 20 mg/L ethyl acetate
73	36.2			36.2	at least one fermentation with > 20 mg/L ethyl acetate
313	36.5			36.5	at least one fermentation with > 20 mg/L ethyl acetate
102	36.5			36.5	at least one fermentation with > 20 mg/L ethyl acetate
321	36.6			36.6	at least one fermentation with > 20 mg/L ethyl acetate
350	36.7			36.7	at least one fermentation with > 20 mg/L ethyl acetate
382	36.8			36.8	at least one fermentation with > 20 mg/L ethyl acetate
122	36.8			36.8	at least one fermentation with > 20 mg/L ethyl acetate
100	36.8			36.8	at least one fermentation with > 20 mg/L ethyl acetate
286	37.1			37.1	at least one fermentation with > 20 mg/L ethyl acetate
147	37.1			37.1	at least one fermentation with > 20 mg/L ethyl acetate
150	37.3			37.3	at least one fermentation with > 20 mg/L ethyl acetate
132	37.4			37.4	at least one fermentation with > 20 mg/L ethyl acetate
23	37.4			37.4	at least one fermentation with > 20 mg/L ethyl acetate
98	37.6			37.6	at least one fermentation with > 20 mg/L ethyl acetate
92	37.8			37.8	at least one fermentation with > 20 mg/L ethyl acetate
156	37.9			37.9	at least one fermentation with > 20 mg/L ethyl acetate
153	38.0			38.0	at least one fermentation with > 20 mg/L ethyl acetate
237	38.1			38.1	at least one fermentation with > 20 mg/L ethyl acetate
283	38.1			38.1	at least one fermentation with > 20 mg/L ethyl acetate
133	38.2			38.2	at least one fermentation with > 20 mg/L ethyl acetate
80	38.2			38.2	at least one fermentation with > 20 mg/L ethyl acetate
273	38.2			38.2	at least one fermentation with > 20 mg/L ethyl acetate
35	38.4			38.4	at least one fermentation with > 20 mg/L ethyl acetate

161	38.6			38.6	at least one fermentation with > 20 mg/L ethyl acetate
227	38.7			38.7	at least one fermentation with > 20 mg/L ethyl acetate
317	39.0			39.0	at least one fermentation with > 20 mg/L ethyl acetate
221	39.1			39.1	at least one fermentation with > 20 mg/L ethyl acetate
1	39.2			39.2	at least one fermentation with > 20 mg/L ethyl acetate
276	39.4			39.4	at least one fermentation with > 20 mg/L ethyl acetate
300	39.4			39.4	at least one fermentation with > 20 mg/L ethyl acetate
299	39.5			39.5	at least one fermentation with > 20 mg/L ethyl acetate
303	39.7			39.7	at least one fermentation with > 20 mg/L ethyl acetate
61	39.8			39.8	at least one fermentation with > 20 mg/L ethyl acetate
272	40.0			40.0	at least one fermentation with > 20 mg/L ethyl acetate
125	40.3			40.3	at least one fermentation with > 20 mg/L ethyl acetate
343	40.4			40.4	at least one fermentation with > 20 mg/L ethyl acetate
177	40.5			40.5	at least one fermentation with > 20 mg/L ethyl acetate
21	40.6			40.6	at least one fermentation with > 20 mg/L ethyl acetate
204	40.8			40.8	at least one fermentation with > 20 mg/L ethyl acetate
180	40.9			40.9	at least one fermentation with > 20 mg/L ethyl acetate
34	41.1			41.1	at least one fermentation with > 20 mg/L ethyl acetate
324	41.2			41.2	at least one fermentation with > 20 mg/L ethyl acetate
220	41.3			41.3	at least one fermentation with > 20 mg/L ethyl acetate
191	41.4			41.4	at least one fermentation with > 20 mg/L ethyl acetate
341	41.5			41.5	at least one fermentation with > 20 mg/L ethyl acetate
111	41.5			41.5	at least one fermentation with > 20 mg/L ethyl acetate
251	41.5			41.5	at least one fermentation with > 20 mg/L ethyl acetate
214	41.6			41.6	at least one fermentation with > 20 mg/L ethyl acetate
184	41.7			41.7	at least one fermentation with > 20 mg/L ethyl acetate
91	41.8			41.8	at least one fermentation with > 20 mg/L ethyl acetate
6	42.0			42.0	at least one fermentation with > 20 mg/L ethyl acetate
379	42.4			42.4	at least one fermentation with > 20 mg/L ethyl acetate
230	43.1			43.1	at least one fermentation with > 20 mg/L ethyl acetate
140	43.2			43.2	at least one fermentation with > 20 mg/L ethyl acetate
275	43.6			43.6	at least one fermentation with > 20 mg/L ethyl acetate

169	44.0			44.0	at least one fermentation with > 20 mg/L ethyl acetate
124	44.1			44.1	at least one fermentation with > 20 mg/L ethyl acetate
226	44.2			44.2	at least one fermentation with > 20 mg/L ethyl acetate
374	44.2			44.2	at least one fermentation with > 20 mg/L ethyl acetate
16	44.3			44.3	at least one fermentation with > 20 mg/L ethyl acetate
82	44.7			44.7	at least one fermentation with > 20 mg/L ethyl acetate
305	44.8			44.8	at least one fermentation with > 20 mg/L ethyl acetate
292	44.8			44.8	at least one fermentation with > 20 mg/L ethyl acetate
148	45.5			45.5	at least one fermentation with > 20 mg/L ethyl acetate
40	45.7			45.7	at least one fermentation with > 20 mg/L ethyl acetate
334	45.9			45.9	at least one fermentation with > 20 mg/L ethyl acetate
327	46.0			46.0	at least one fermentation with > 20 mg/L ethyl acetate
12	46.5			46.5	at least one fermentation with > 20 mg/L ethyl acetate
216	46.7			46.7	at least one fermentation with > 20 mg/L ethyl acetate
271	46.7			46.7	at least one fermentation with > 20 mg/L ethyl acetate
380	46.9			46.9	at least one fermentation with > 20 mg/L ethyl acetate
175	46.9			46.9	at least one fermentation with > 20 mg/L ethyl acetate
159	47.1			47.1	at least one fermentation with > 20 mg/L ethyl acetate
339	47.2			47.2	at least one fermentation with > 20 mg/L ethyl acetate
362	47.3			47.3	at least one fermentation with > 20 mg/L ethyl acetate
366	47.3			47.3	at least one fermentation with > 20 mg/L ethyl acetate
311	47.6			47.6	at least one fermentation with > 20 mg/L ethyl acetate
377	48.1			48.1	at least one fermentation with > 20 mg/L ethyl acetate
291	48.4			48.4	at least one fermentation with > 20 mg/L ethyl acetate
258	48.6			48.6	at least one fermentation with > 20 mg/L ethyl acetate
58	48.7			48.7	at least one fermentation with > 20 mg/L ethyl acetate
274	49.0			49.0	at least one fermentation with > 20 mg/L ethyl acetate
97	49.2			49.2	at least one fermentation with > 20 mg/L ethyl acetate
85	49.3			49.3	at least one fermentation with > 20 mg/L ethyl acetate
212	49.7			49.7	at least one fermentation with > 20 mg/L ethyl acetate
328	50.0			50.0	at least one fermentation with > 20 mg/L ethyl acetate
367	50.5			50.5	at least one fermentation with > 20 mg/L ethyl acetate

93	50.5			50.5	at least one fermentation with > 20 mg/L ethyl acetate
89	51.3			51.3	at least one fermentation with > 20 mg/L ethyl acetate
141	53.0			53.0	at least one fermentation with > 20 mg/L ethyl acetate
242	53.0			53.0	at least one fermentation with > 20 mg/L ethyl acetate
222	53.1			53.1	at least one fermentation with > 20 mg/L ethyl acetate
349	53.4			53.4	at least one fermentation with > 20 mg/L ethyl acetate
109	53.5			53.5	at least one fermentation with > 20 mg/L ethyl acetate
76	53.5			53.5	at least one fermentation with > 20 mg/L ethyl acetate
340	53.8			53.8	at least one fermentation with > 20 mg/L ethyl acetate
295	54.3			54.3	at least one fermentation with > 20 mg/L ethyl acetate
30	55.3			55.3	at least one fermentation with > 20 mg/L ethyl acetate
27	55.8			55.8	at least one fermentation with > 20 mg/L ethyl acetate
255	55.8			55.8	at least one fermentation with > 20 mg/L ethyl acetate
354	56.2			56.2	at least one fermentation with > 20 mg/L ethyl acetate
81	56.4			56.4	at least one fermentation with > 20 mg/L ethyl acetate
284	56.7			56.7	at least one fermentation with > 20 mg/L ethyl acetate
56	56.8			56.8	at least one fermentation with > 20 mg/L ethyl acetate
66	57.6			57.6	at least one fermentation with > 20 mg/L ethyl acetate
3	57.7			57.7	at least one fermentation with > 20 mg/L ethyl acetate
69	57.9			57.9	at least one fermentation with > 20 mg/L ethyl acetate
115	57.9			57.9	at least one fermentation with > 20 mg/L ethyl acetate
32	58.4			58.4	at least one fermentation with > 20 mg/L ethyl acetate
105	59.5			59.5	at least one fermentation with > 20 mg/L ethyl acetate
129	59.8			59.8	at least one fermentation with > 20 mg/L ethyl acetate
280	60.3			60.3	at least one fermentation with > 20 mg/L ethyl acetate
120	60.4			60.4	at least one fermentation with > 20 mg/L ethyl acetate
77	61.6			61.6	at least one fermentation with > 20 mg/L ethyl acetate
19	62.3			62.3	at least one fermentation with > 20 mg/L ethyl acetate
103	62.7			62.7	at least one fermentation with > 20 mg/L ethyl acetate
121	63.6			63.6	at least one fermentation with > 20 mg/L ethyl acetate
87	64.7			64.7	at least one fermentation with > 20 mg/L ethyl acetate
52	65.7			65.7	at least one fermentation with > 20 mg/L ethyl acetate

331	66.1			66.1	at least one fermentation with > 20 mg/L ethyl acetate
48	66.2			66.2	at least one fermentation with > 20 mg/L ethyl acetate
68	67.6			67.6	at least one fermentation with > 20 mg/L ethyl acetate
94	67.6			67.6	at least one fermentation with > 20 mg/L ethyl acetate
9	72.4			72.4	at least one fermentation with > 20 mg/L ethyl acetate
155	74.8			74.8	at least one fermentation with > 20 mg/L ethyl acetate
236	79.4			79.4	at least one fermentation with > 20 mg/L ethyl acetate
333	80.3			80.3	at least one fermentation with > 20 mg/L ethyl acetate

Supplementary Table S4. Primers used in this study.

Underlined nucleotides indicate nucleotides that are complementary to plasmid DNA. For allele specific PCR, bold nucleotides indicate ER7A mutations compared to S288c.

Name	Function	Sequence
	**Deletions and verification**	
URA3Δ FW	*URA3* deletion cassette (pUG6)	ACTGCACAGAACAAAAACCTGCAGGAAACGAAGATAAATCCAGCTGAAGCTTCGTACGCTGCAG
URA3Δ RV	*URA3* deletion cassette (pUG6)	GTGAGTTTAGTATACATGCATTTACTTATAATACAGTTTTGCATAGGCCACTAGTGGATCTGATATC
URA3 -297 FW	Verification of *URA3* deletion, upstream of *URA3*	CGAGGCATATTTATGGTGAAG
URA3 +239 RV	Verification of *URA3* deletion, downstream of *URA3*	CTTTGGAGTTCAATGCGTCCATC
KANMX FW	Verification of *URA3* deletion, inside KANMX	CCAGAGTTGTTTCTGAAACATG
KANMX RV	Verification of deletion, inside KANMX	CTCGTCCAACATCAATACAAC
GIN11 FW	FW primer for part B of deletion cassette for gene replacement	CCAGAAATAAGGCTAAAAAACTAATCGCATTATCATCC
GIN11 RV	RV primer for part A of deletion cassette for gene replacement	GCACATTTCCAAGTTCAGTATTACTTATGGACAC
PMA1Δ FW	*PMA1* deletion cassette part A (pBluescriptII_NAT1_GIN11_SSK1) part A and B have 600 bp overlap and were transformed in equimolar amounts	AAGAAAATCATTGAAAAGAATAAGAAGATAAGAAAGATTTAATTATCAAACAATATCAATCCAGATCTGTTTAGCTTGCCTTGTCC
PMA1Δ RV	*PMA1* deletion cassette part B (pBluescriptII_NAT1_GIN11_SSK1) part A and B have 600 bp overlap and were transformed in equimolar amounts	AGAAAAATCAAGTTGATTAAAATGTGACAAAAATTATGATTAAATGCTACTTCAACAGGACGATAAGCTTGATATCGAATTCCTGCAG
PMA1 insertion FW	*PMA1* insertion cassette	CTCAGCTTTGCTAAAGTGCAAAAAGTCGTTTACGTCACCATCGCTGACGGGGAAAAAACAAAACAAGCTTC
PMA1 insertion RV	*PMA1* insertion cassette	GCGCCAGGGTAGTATACTATAGAAGGTCAGACTAAACTGAGTCATCTAGAGTAATGACGCCTTAGTAGC
PMA1 A FW	Verification of *PMA1* insertion, upstream	CATTTCTTTACTAAACCCGAC
PMA1 A RV	Verification of *PMA1* insertion, upstream	CATCGACCACGTTTCGTCACCGGTCA
PMA1 B FW	Verification of *PMA1* insertion, downstream	AAACGCACACAAGTCATAATTATTC
PMA1 B RV	Verification of *PMA1* insertion, downstream	CTTGTGGAGAGGTGACTTCATGAAC
CEM1Δ FW	*CEM1* deletion cassette part A (pBluescriptII_NAT1_GIN11_SSK1) part A and B have 600 bp overlap and were transformed in equimolar amounts	TAGCAACTTGAAAGTGACAGGAGCCACAACGGATTAAAATTTAATTTCTAGTAAAGAAATCAATCCAGATCTGTTTAGCTTGCCTTGTCC
CEM1Δ RV	*CEM1* deletion cassette part B (pBluescriptII_NAT1_GIN11_SSK1) part A and B have 600 bp overlap and were transformed in equimolar amounts	AATAGATAGCATATAATTCAAAAACGATTATAATGGGATATATATATAAGTTTAATAATACGATAAGCTTGATATCGAATTCCTGCAG
CEM1 insertion FW	*CEM1* insertion cassette	CGACGGTACCATTATCATCTCTCAACCAGCCATTGCTGCATTCTGGAGGGATTTCGGTAAG
CEM1 insertion RV	*CEM1* insertion cassette	CTATCGAGATCAGGAAAATTTAAAGGTGGTGAATGGGTAGGGGGTGCAACTACTGCAGGTG
CEM1 A FW	Verification of *CEM1* insertion, upstream	CCAGATCAGTGGAAAAACATAAAAC
CEM1 A RV	Verification of *CEM1* insertion, upstream	GTAGTGGCAATACCAATGATCTTACAAC
CEM1 B FW	Verification of *CEM1* insertion, downstream	GAAATACCTAAAGTGGCAGCATTAC
CEM1 B RV	Verification of *CEM1* insertion, downstream	CTAAATTTCGGCCAAAGTGTTTTAG

TPS1Δ FW	*TPS1* deletion cassette part A (pBluescriptII_NAT1_GIN11_SSK1)	AGCAACAAAGCAGGCTAACAAACTAGGTACTCACATACAGACTTATTAAGACATAGAACTCCAGATCTGTTTAGCTTGCCTTGTCC
TPS1Δ RV	*TPS1* deletion cassette part B (pBluescriptII_NAT1_GIN11_SSK1)	AAGAGAGGGCAGAGAAAACCGGACCAGGAATAGACGATCGTCTCATTTGCATCGGGTTCACGATAAGCTTGATATCGAATTCCTGCAG
TPS1 insertion FW	*TPS1* insertion cassette	TTACAGCTCCAACAAGAAGGCAGGTGTTGTCTTTATACAAGGAATTCATCAAGAATGC
TPS1 insertion RV	*TPS1* insertion cassette	CTTGCATTGCACCATAGCTTATTAGTATTTTGTTTTATGATCATTACGTCTTCTTACTC
TPS1 A FW	Verification of *TPS1* insertion, upstream	GGTTATATTACCGAGGGTCAAATC
TPS1 A RV	Verification of *TPS1* insertion, upstream	CTTCAGCAACCGTCCTCTTAG
TPS1 B FW	Verification of *TPS1* insertion, downstream	CCTATATAGCCATTTTAGAAAGTAAC
TPS1 B RV	Verification of *TPS1* insertion, downstream	CGTCTGGTTCAACGGTAACTTGTG
NATMX FW	Verification of correct integration of NATMX cassette in RHA	GAGCATGCCCTGCCCCTAATC
NATMX RV	Verification of correct integration of NATMX cassette in RHA	GTGTCGTCAAGAGTGGTACCCATG
MAT locus	Mating type PCR	AGTCACATCAAGATCGTTTATGG
MATα	Mating type PCR, MATα-specific	GCACGGAATATGGGACTACTTCG
MATa	Mating type PCR, MAT**a**-specific	ACTCCACTTCAAGTAAGAGTTTG
**RHA chr II**		
RHA TPS1Δ FW	*TPS1* deletion cassette (pAG25)	AGCAACAAAGCAGGCTAACAAACTAGGTACTCACATACAGACTTATTAAGACATAGAACTCAGCTGAAGCTTCGTACGCTGCAG
RHA TPS1Δ RV	*TPS1* deletion cassette (pAG25)	AAGAGAGGGCAGAGAAAACCGGACCAGGAATAGACGATCGTCTCATTTGCATCGGGTTCAGCATAGGCCACTAGTGGATCTGATATCATC
TPS1 FW	Verification of *TPS1* deletion, upstream of *TPS1,* with NATMX RV	CATCTGCACCAGAACAAAGAACAAA
TPS1 RV	Verification of *TPS1* deletion, downstream of *TPS1* with NATMX FW	CGTGCTAAGAAAGAAGAAAAAGATG
TPS1 seq FW	Sequencing TPS1 for ER7a SNPs	GAGGAGCTTGTTGAGCTTGA
TPS1 seq RV	Sequencing TPS1 for ER7a SNPs	TTGAAGGAAACTTTCAAGGG
RHA YBR126W-AΔ FW	*YBR126W-A* deletion cassette (pAG25)	CCCGACCTTAACGTTTTGCAGTTGCTTCTCGTGAATCTTGACTCAGCTGAAGCTTCGTACGCTGCAG
RHA YBR126W-AΔ RV	*YBR126W-A* deletion cassette (pAG25)	CAAGATTGGCCTTGACCACCGCTGAATACCTTGCTTACCAGCATAGGCCACTAGTGGATCTGATATCATC
YBR126W-A FW	Verification of *YBR126W-A* deletion, upstream of *YBR126W-A,* with NATMX RV	AAGACGAGGTCAGTTGCGCC
YBR126W-A RV	Verification of *YBR126W-A* deletion, downstream of *YBR126W-A* with NATMX FW	TGCCTTCGTTGAGTAGATTG
YBR126W-A seq FW	Sequencing YBR126W-A for ER7a SNPs	ATGGCTATCACCCCTGACAA
YBR126W-A seq RV	Sequencing YBR126W-A for ER7a SNPs	TCAAGCGTGAGGAATGCCGTCC
RHA YBR126W-BΔ FW	*YBR126W-B* deletion cassette (pAG25)	CATGCACCCAGATTAAATCGTTATGGTTCATAGTCTTAGCAACAGCTGAAGCTTCGTACGCTGCAG
RHA YBR126W-BΔ RV	*YBR126W-B* deletion cassette (pAG25)	AAGAATTATCACTCCAAGATTGGCCTTGACCACCGCTGAGCATAGGCCACTAGTGGATCTGATATCATC
YBR126W-B FW	Verification of *YBR126W-B* deletion, upstream of *YBR126W-B,* with NATMX RV	TCACGGGAAGCCTGTTGGACAC
YBR126W-B RV	Verification of *YBR126W-B* deletion, downstream of *YBR126W-B* with NATMX FW	AGGAGATGTTGAATAGAATC
YBR126W-B seq FW	Sequencing YBR126W-B for ER7a SNPs	ATGCAAGTGTATTGCAAT
YBR126W-B seq RV	Sequencing YBR126W-B for ER7a SNPs	TCACCCTGGGCACATCAAGCGT
RHA VMA2Δ FW	*VMA2* deletion cassette (pAG25)	AAATGAGCGGATTTATAGTAAAAGGAATCACATCAGCGAATTGAAACAGCTGAAGCTTCGTACGCTGCAG

RHA VMA2Δ RV	*VMA2* deletion cassette (pAG25)	TGTCCCGTACTGCTGTTTTTAGTGATTGTCACGGGAAGCGCATAGGCCACTAGTGGATCTGATATCATC
VMA2 FW	Verification of *VMA2* deletion, upstream of *VMA2,* with NATMX RV	CGATGTGGATGGGCTGTTCT
VMA2 RV	Verification of *VMA2* deletion, downstream of *VMA2* with NATMX FW	CTCTCCCTGGCGCGCTGG
VMA2 seq FW	Sequencing VMA2 for ER7a SNPs	ATGGTTTTGTCTGATAAG
VMA2 seq RV	Sequencing VMA2 for ER7a SNPs	TTAGATTAGAGATTCTTCTTG
RHA ATG14Δ FW	*ATG14* deletion cassette (pAG25)	GAAGGATAACGAGTAGAGAAAAAGGGAAGTAAAAGTTAAAAACTAGAATCCTAGTATGACCAGCTGAAGCTTCGTACGCTGCAG
RHA ATG14Δ RV	*ATG14* deletion cassette (pAG25)	TATTGTTTATGACTGACTACATGCAACTTTATACACACGGCAGGAAAAAAAGTGCGCACTGCATAGGCCACTAGTGGATCTGATATCATC
ATG14 FW	Verification of *ATG14* deletion, upstream of *ATG14,* with NATMX RV	AAATTATCGATTGTATTGAACTTGA
ATG14 RV	Verification of *ATG14* deletion, downstream of *ATG14* with NATMX FW	GTCAAATTAACAATTTCGTTGTAAC
ATG14 seq FW	Sequencing ATG14 for ER7a SNPs	ATAATAACCTGATTAGGCAC
ATG14 seq RV	Sequencing ATG14 for ER7a SNPs	TAGCGAGACGGACCTACACT
RHA OPY1Δ FW	*OPY1* deletion cassette (pAG25)	GTAGGTGAATTAACCATAAATAATTACGCTAATTTTGCCTTGGTAAATTTCATAAGACCACAGCTGAAGCTTCGTACGCTGCAG
RHA OPY1Δ RV	*OPY1* deletion cassette (pAG25)	ACGTATTATTTAATAGTACAAAGAAAAAAAAAGTCAATACATATTCATCATTGGCATTGCGCATAGGCCACTAGTGGATCTGATATCATC
OPY1 FW	Verification of *OPY1* deletion, upstream of *OPY1,* with NATMX RV	GAACTGCCTCAGTAAGAAAACCAAG
OPY1 RV	Verification of *OPY1* deletion, downstream of *OPY1* with NATMX FW	TTGATATCTAACTGGTCATAGTTCA
OPY1 seq FW	Sequencing OPY1 for ER7a SNPs	GTCAGCATGAAATTTTGATC
OPY1 seq RV	Sequencing OPY1 for ER7a SNPs	TAAAGAATCCCAGAACAAAC
RHA SHE3Δ FW	*SHE3* deletion cassette (pAG25)	ACCAAACGTTAGCTCGTCTATCAAGCACGCCAAGGTTCAACGACACTACTTTTGTGTAAGCAGCTGAAGCTTCGTACGCTGCAG
RHA SHE3Δ RV	*SHE3* deletion cassette (pAG25)	TTACTTTTGTTCTATTATCTAAATGAATCCTATATATATACTCCCTTGTGTCGGCATATTGCATAGGCCACTAGTGGATCTGATATCATC
SHE3 FW	Verification of *SHE3* deletion, upstream of *SHE3,* with NATMX RV	GGACAGTTTTTCGTTTAAAGAGGGT
SHE3 RV	Verification of *SHE3* deletion, downstream of *SHE3* with NATMX FW	TGGTGATGATGGTGATGATGGTAAC
SHE3 seq FW	Sequencing SHE3 for ER7a SNPs	AGTAATCAAAATTTAAATTA
SHE3 seq RV	Sequencing SHE3 for ER7a SNPs	GTAACACCGGGAGGCACTGA
RHA CCZ1Δ FW	*CCZ1* deletion cassette (pAG25)	GTGGAACCAAAAATACATTCTAAATCGTACAACTATTAAATTGATATATGAAAGACGGATCAGCTGAAGCTTCGTACGCTGCAG
RHA CCZ1Δ RV	*CCZ1* deletion cassette (pAG25)	TCCTTTTTTTATAGTTACTTATTTTTATTCTATGTCTATCAAATGCTAAACGTTACATTTGCATAGGCCACTAGTGGATCTGATATCATC
CCZ1 FW	Verification of *CCZ1* deletion, upstream of *CCZ1,* with NATMX RV	GAGGTATTTCTATCTTTTGTGGGGG
CCZ1 RV	Verification of *CCZ1* deletion, downstream of *CCZ1* with NATMX FW	CATGAGATTGAAAGCTCCTTCGAAA
CCZ1 seq FW	Sequencing CCZ1 for TDA1(4) SNPs	AAAAATACATTCTAAATCGT
CCZ1 seq RV	Sequencing CCZ1 for TDA1(4) SNPs	AGGATTATAAGAGGAATGTT
CCZ1 seq FW	Sequencing CCZ1 for ER7a SNPs	ATTGTGTCAGAGTATGGAAT
CCZ1 seq RV	Sequencing CCZ1 for ER7a SNPs	TCACGACCTAAACTTTGAAA

RHA AGP2Δ FW	*AGP2* deletion cassette (pAG25)	CCTTCAGCCTTGTCCCCCCTAAGCTGCACTTACATTTTGCTTCCATAACTTTTGCCAAGCCAGCTGAAGCTTCGTACGCTGCAG
RHA AGP2Δ RV	*AGP2* deletion cassette (pAG25)	ATCTGACAATAAATTTGGAGGCAGTCAATGTAAATTTGTGAATATAACGACATAATTGCAGCATAGGCCACTAGTGGATCTGATATCATC
AGP2 FW	Verification of *AGP2* deletion, upstream of *AGP2,* with NATMX RV	AGTCAAGACTAGTGTTTCCACGCTA
AGP2 RV	Verification of *AGP2* deletion, downstream of *AGP2* with NATMX FW	CAAAGTTTAGGTCGTGACGTGACTG
AGP2 seq FW	Sequencing AGP2 for ER7a SNPs	CGATCGGAAAAGCTTTATAC
AGP2 seq RV	Sequencing AGP2 for ER7a SNPs	GCCTTGGGTAATACTTTTCT
RHA HSL7Δ FW	*HSL7* deletion cassette (pAG25)	CGGACGTTCCTCTTCTTTTATACATATAATTTTTATATATACAAAGGGTTCAGTTTGCATCAGCTGAAGCTTCGTACGCTGCAG
RHA HSL7Δ RV	*HSL7* deletion cassette (pAG25)	GGTACTCAGGACGTATGGATAGTTATTTGTTGCCGCAGTATATAGTATACAATGCAGAATGCATAGGCCACTAGTGGATCTGATATCATC
HSL7 FW	Verification of *HSL7* deletion, upstream of *HSL7,* with NATMX RV	CTCCATTCTTTATTTATCTCATACC
HSL7 RV	Verification of *HSL7* deletion, downstream of *HSL7* with NATMX FW	TATTCTTATCGTACTCAAAATCACC
HSL7 seq FW	Sequencing HSL7 for ER7a SNPs	GGTGTCAAACCAGGCTTTAA
HSL7 seq RV	Sequencing HSL7 for ER7a SNPs	GAAGGAGTCCTGGTTCTTGG
RHA YBR134WΔ FW	*YBR134W* deletion cassette (pAG25)	CAAAAGTCAAATATGTGGAATTTAATAAATTATCTGAATGAGGTTTCAGAGGCGGCATTACAGCTGAAGCTTCGTACGCTGCAG
RHA YBR134WΔ RV	*YBR134W* deletion cassette (pAG25)	GTGTATGTCGTTCTTGTTGTCCAGCCTCACTTTGTCTTCATTAATCATCTCTTTTGCATAGGCCACTAGTGGATCTGATATCATC
YBR134W FW	Verification of *YBR134W* deletion, upstream of *YBR134W,* with NATMX RV	ATGGATCGCTGTCTTCGTAAAG
YBR134W RV	Verification of *YBR134W* deletion, downstream of *YBR134W* with NATMX FW	CCTTTTGAATAGCAAAATG
YBR134W seq FW	Sequencing YBR134W for ER7a SNPs	ATGATTAAAGCCTGGTTTG
YBR134W seq RV	Sequencing YBR134W for ER7a SNPs	TTAGACCCTAAAACAGATC
RHA CKS1Δ FW	*CKS1* deletion cassette (pAG25)	ACTGCTGGCCGCGTTGGATACAACTAGATTGACACGCACTTACTCTCGTCTTACAGTACTCAGCTGAAGCTTCGTACGCTGCAG
RHA CKS1Δ RV	*CKS1* deletion cassette (pAG25)	TCCATATAGTTATGGAGCCTTGAAAAGGAATTCAATTTCAGTAATTAGAGTATATCAAAGGCATAGGCCACTAGTGGATCTGATATCATC
CKS1 FW	Verification of *CKS1* deletion, upstream of *CKS1,* with NATMX RV	ACAAGTCACTCGAAATATACCATTT
CKS1 RV	Verification of *CKS1* deletion, downstream of *CKS1* with NATMX FW	GAGTTCAGGTCTTTTATTGC
CKS1 seq FW	Sequencing CKS1 for ER7a SNPs	ATGTACCATCACTATCACGC
CKS1 seq RV	Sequencing CKS1 for ER7a SNPs	CTAGGAGATTTGGGGTGGAA
RHA MEC1Δ FW	*MEC1* deletion cassette (pAG25)	AGACAAAGTGAGGCTGGACAACAAGAACGACATACACCGCGTAAAGGCCCACAAGACTGCCAGCTGAAGCTTCGTACGCTGCAG
RHA MEC1Δ RV	*MEC1* deletion cassette (pAG25)	CCTGCAGTGATGGTTAGATCAAGAGGAAGTTCGTCTGTTGCCGAAAATGGTGGAAAGTCGGCATAGGCCACTAGTGGATCTGATATCATC
MEC1 FW	Verification of *MEC1* deletion, upstream of *MEC1,* with NATMX RV	GAGGTATTTCTATCTTTTGTGGGGG
MEC1 RV	Verification of *MEC1* deletion, downstream of *MEC1* with NATMX FW	TTCTTTAACTGCATTTCTTATGTAA
MEC1 seq FW	Sequencing MEC1 for TDA1(4) SNPs	AAAAATACATTCTAAATCGT
MEC1 seq RV	Sequencing MEC1 for TDA1(4) SNPs	AGGATTATAAGAGGAATGTT
MEC1 seq FW	Sequencing MEC1 for ER7a SNPs	ATATTGAGAAAACTGGCAAC

MEC1 seq RV	Sequencing MEC1 for ER7a SNPs	TTGAAAGCCAACTTTTCTTT
RHA YBR137WΔ FW	*YBR137W* deletion cassette (pAG25)	CCTAATCAAGCCTTATTATAAGAGCAAATTATTCAAAAAAAGTCTACGGAGAAAATTATTCAGCTGAAGCTTCGTACGCTGCAG
RHA YBR137WΔ RV	*YBR137W* deletion cassette (pAG25)	AGTAACGGGTAAAAAATATTCTATGGTGTTCAAGCCTCTATTTTACTTCTACCTACATTCGCATAGGCCACTAGTGGATCTGATATCATC
YBR137W FW	Verification of *YBR137W* deletion, upstream of *YBR137W,* with NATMX RV	AAATCCGCGGTATAGATCCGCAGGA
YBR137W RV	Verification of *YBR137W* deletion, downstream of *YBR137W* with NATMX FW	CCTTAAAAAGGAAATATCAAAAAAA
YBR137W seq FW	Sequencing YBR137W for TDA1(4) SNPs	TTTTAGACAAGAAGTTATTG
YBR137W seq RV	Sequencing YBR137W for TDA1(4) SNPs	ACAATCAATATATATATGAA
YBR137W seq FW	Sequencing YBR137W for ER7a SNPs	TTTTAGACAAGAAGTTATTG
YBR137W seq RV	Sequencing YBR137W for ER7a SNPs	ACAATCAATATATATATGAA
RHA YBR138CΔ FW	*YBR138C* deletion cassette (pAG25)	CAGATTGGAATAGTCTCGAAAACTTGATCCAACCACTAATTAAAAGGTTAAAATTAAAGGCAGCTGAAGCTTCGTACGCTGCAG
RHA YBR138CΔ RV	*YBR138C* deletion cassette (pAG25)	AAAAAATACACAGCACCAATGCAGTCCTGTTTGTTTTAGTGATCTCTTAGCAATGTATTTGCATAGGCCACTAGTGGATCTGATATCATC
YBR138C FW	Verification of *YBR138C* deletion, upstream of *YBR138C,* with NATMX RV	AGGCTCGGCCGAATGAGGCGTATTT
YBR138C RV	Verification of *YBR138C* deletion, downstream of *YBR138C* with NATMX FW	TGGTCATTCAAGTTTCTATATGGGC
YBR138C seq FW	Sequencing YBR138C for TDA1(4) SNPs	GGTATGTAGTCTTCAACCAG
YBR138C seq RV	Sequencing YBR138C for TDA1(4) SNPs	CACAAACTCATTATCCTTGC
YBR138C seq FW	Sequencing YBR138C for ER7a SNPs	ATGGAGAAAGATCAAATCCA
YBR138C seq RV	Sequencing YBR138C for ER7a SNPs	TGTTGTTGTATTCGTTGTTT
RHA YBR139WΔ FW	*YBR139W* deletion cassette (pAG25)	AAATTGTCATCCTGCATTTTTTCTTTAAAACAGCTATACAAAAAGTGATACCGACATACACAGCTGAAGCTTCGTACGCTGCAG
RHA YBR139WΔ RV	*YBR139W* deletion cassette (pAG25)	AGAGATTTGTGTAAAGTATAATGATTGCAAGGTTAAGAGTGAAACATTTTTAGAGCACTAGCATAGGCCACTAGTGGATCTGATATCATC
YBR139W FW	Verification of *YBR139W* deletion, upstream of *YBR139W,* with NATMX RV	TTGCTATTCATATTTGAGCTGGTAT
YBR139W RV	Verification of *YBR139W* deletion, downstream of *YBR139W* with NATMX FW	CCCTAGAACAATCATCTGAAGCGGT
YBR139W seq FW	Sequencing YBR139W for ER7a SNPs	CCAATTATTCTTTGGTTAAA
YBR139W seq RV	Sequencing YBR139W for ER7a SNPs	CCTCTAATGTCATAGACGTT
RHA IRA1Δ FW	*IRA1* deletion cassette (pAG25)	TTCAAACTTAACATTCTTCTTCAGCATATAACATACAACAAGATTAAGGCTCTTTCTAAACAGCTGAAGCTTCGTACGCTGCAG
RHA IRA1Δ RV	*IRA1* deletion cassette (pAG25)	AAAACAAAATATAATTATAAGGAAAAACGTATATAATCACTGCAATACTCTAATTTAAAAGCATAGGCCACTAGTGGATCTGATATCATC
IRA1 FW	Verification of *IRA1* deletion, upstream of *IRA1,* with NATMX RV	GGTTGAACAATTTTGGTATTACCCT
IRA1 RV	Verification of *IRA1* deletion, downstream of *IRA1* with NATMX FW	TGGGAAACCATGCTTGGTCCAATGA
IRA1 seq FW	Sequencing IRA1 for TDA1(4) SNPs	CTTTTATTGAAATCCCATTAAG
IRA1 seq RV	Sequencing IRA1 for TDA1(4) SNPs	TAGTTCCTCTTGCGTAACTG
IRA1 seq FW	Sequencing IRA1 for ER7a SNPs	CTTTTATTGAAATCCCATTAAG
IRA1 seq RV	Sequencing IRA1 for ER7a SNPs	TAGTTCCTCTTGCGTAACTG
RHA BMT2Δ FW	*BMT2* deletion cassette (pAG25)	GCTAGCCATGACCTTGATGGCAATCTTGTATCAGCTTAGCGATACCACTAATACAGCGGACAGCTGAAGCTTCGTACGCTGCAG

RHA BMT2Δ RV	*BMT2* deletion cassette (pAG25)	TTTACACATCTTTCTATATTGTATCCCTTTATGAAAAACTTCCAGGCGGTGTCGGCTCGCGCATAGGC CACTAGTGGATCTGATATCATC
BMT2 FW	Verification of *BMT2* deletion, upstream of *BMT2,* with NATMX RV	AATTTCTTCCAAGCCATAAAATCCA
BMT2 RV	Verification of *BMT2* deletion, downstream of *BMT2* with NATMX FW	TTTTTGTCTTGCGGATCGCTTTGAT
BMT2 seq FW	Sequencing BMT2 for TDA1(4) SNPs	GAAGGCGTTATTAAGCAGGA
BMT2 seq RV	Sequencing BMT2 for TDA1(4) SNPs	AATAAAATGATCAAAGGCGT
BMT2 seq FW	Sequencing BMT2 for ER7a SNPs	ATGAAGGCGTTATTAAGCAG
BMT2 seq RV	Sequencing BMT2 for ER7a SNPs	TAAAATGATCAAAGGCGTAA
RHA MAK5Δ FW	*MAK5* deletion cassette (pAG25)	TCATCATAACCTCAGGTAGAAATTCTTTAGTCAAATACCGGCATAATCAGTTTTTCCATCCAGCTGAA GCTTCGTACGCTGCAG
RHA MAK5Δ RV	*MAK5* deletion cassette (pAG25)	ACCGTGTATATTACGTAGAAAATACAGTGAAAGGAGAGTTTCTCTTCAAAAGCCTCGAGAGCATAG GCCACTAGTGGATCTGATATCATC
MAK5 FW	Verification of *MAK5* deletion, upstream of *MAK5,* with NATMX RV	AAACTTGCATATCGATTGACGCTTA
MAK5 RV	Verification of *MAK5* deletion, downstream of *MAK5* with NATMX FW	GCAGCTAACTACAAAAACTTCGGTG
MAK5 seq FW	Sequencing MAK5 for ER7a SNPs	AAACTTTAGCCTATGGTATT
MAK5 seq RV	Sequencing MAK5 for ER7a SNPs	TTTTGACTCAGGATTTGTAT
RHA SUP45Δ FW	*SUP45* deletion cassette (pAG25)	TTCATTTCGGCTTGTCTCCTTATTAAGACTACAGAAATAGACAAAGGAAATACTTCAATACAGCTGAA GCTTCGTACGCTGCAG
RHA SUP45Δ RV	*SUP45* deletion cassette (pAG25)	ATCTGGCATCTAGTGATTAAATTCTTTTTGATTCGATTTTTTTCTCCCCCTTTTATTTATGCATAGGCCA CTAGTGGATCTGATATCATC
SUP45 FW	Verification of *SUP45* deletion, upstream of *SUP45,* with NATMX RV	CATGGTATTATAACCAGATCATAAA
SUP45 RV	Verification of *SUP45* deletion, downstream of *SUP45* with NATMX FW	AAAGCCATGAGAGCTACGTTGAACG
SUP45 seq FW	Sequencing SUP45 for ER7a SNPs	GTCCAATCTTTAGAAAAAGC
SUP45 seq RV	Sequencing SUP45 for ER7a SNPs	GTCTTAAAGTCAGCAGAACC
RHA YBR144CΔ FW	*YBR144C* deletion cassette (pAG25)	ACAATACGAAGGAACGATCCCGCGTCAGCAGATGTCACAATTTGATTATTTCATTGTCACCAGCTGA AGCTTCGTACGCTGCAG
RHA YBR144CΔ RV	*YBR144C* deletion cassette (pAG25)	TAAAAAGGTGCTTTCCTCGAAGTTGCGTCGAAGATGAATGAAAGTAGTTGCTAATTCTTAGCATAGG CCACTAGTGGATCTGATATCATC
YBR144C FW	Verification of *YBR144C* deletion, upstream of *YBR144C,* with NATMX RV	TTCGTGACCACCGATTAATGGAAAT
YBR144C RV	Verification of *YBR144C* deletion, downstream of *YBR144C* with NATMX FW	CATGTGCACATTCATCATTGACCTT
YBR144C seq FW	Sequencing YBR144C for ER7a SNPs	ATGCTTTTTGACTCATCCTT
YBR144C seq RV	Sequencing YBR144C for ER7a SNPs	CTAGCACATGAAAAAAAAGG
RHA ADH5Δ FW	*ADH5* deletion cassette (pAG25)	ACTATGTCATTTTGAAGTTGGTTAGAATTTGCTTTTTGGTTTCGCAGCTGAAGCTTCGTACGCTGCAG
RHA ADH5Δ RV	*ADH5* deletion cassette (pAG25)	GTAATCATTCAATTGTCGACCATTAACTAATATCTCACCTGTACCTCTAACAACGAGCATAGGCCACT AGTGGATCTGATATCATC
ADH5 FW	Verification of *ADH5* deletion, upstream of *ADH5,* with NATMX RV	CATAGTCTATCCAAGCGTTTGC
ADH5 RV	Verification of *ADH5* deletion, downstream of *ADH5* with NATMX FW	ACAAAGAAAGCCTTGAAAAC
ADH5 seq FW	Sequencing ADH5 for ER7a SNPs	GGAATATAAAGACGTCACAG
ADH5 seq RV	Sequencing ADH5 for ER7a SNPs	CTCCGCCTAATTGTTCAAATAAC
RHA MRPS9Δ FW	*MRPS9* deletion cassette (pAG25)	AATTGGTTAAATTGATTAGCTAAAATTTACTCGTTGTGGACAGAGTTTGAGCCAAGCGGACAGCTGA AGCTTCGTACGCTGCAG

RHA MRPS9Δ RV	*MRPS9* deletion cassette (pAG25)	TCAAAAAACTCAGAGCACGCACGCTTCCGTTCGAAATTGAGGTAACAAGATGCACTAAGAGCATAGGCCACTAGTGGATCTGATATCATC
MRPS9 FW	Verification of *MRPS9* deletion, upstream of *MRPS9,* with NATMX RV	GATGAATATATTTTTACTTTTTATA
MRPS9 RV	Verification of *MRPS9* deletion, downstream of *MRPS9* with NATMX FW	ATATTGTGGGATATTACAGGAGAAT
MRPS9 seq FW	Sequencing MRPS9 for ER7a SNPs	GATCGTATTAATCGACTCGA
MRPS9 seq RV	Sequencing MRPS9 for ER7a SNPs	TTTCCTGGCTTCTTTCTTTC
**RHA Chr V**		
RHA GAL83_MIG3Δ FW	*GAL83_MIG3* deletion cassette (pAG25)	TCTTTTATGGGGGGTGTTCTGGGGCAGAAACCAAAACGGCGGAACGCGTCCGGAAAAACACAGCTGAAGCTTCGTACGCTGCAG
RHA GAL83_MIG3Δ RV	*GAL83_MIG3* deletion cassette (pAG25)	ATCGCAGAAACATCTTCGCTTTTATCTAAAAAGATGTAGCTATAAGCTCTATAATCATATGCATAGGCCACTAGTGGATCTGATATCATC
GAL83_MIG3 FW	Verification of *GAL83_MIG3* deletion, upstream of *MIG3,* with NATMX RV	AAGATCTCGGAAAAACTGAAAGGCA
GAL83_MIG3 RV	Verification of *GAL83_MIG3* deletion, downstream of *GAL83* with NATMX FW	AAAAATAGGTGTATCTCTCTTTCCT
MIG3 seq FW	Sequencing GAL83	GTGTACGCCAGGGAAGCCTC
MIG3 seq RV	Sequencing GAL83	CTTACTTCAGTAAATAAACA
RHA YEN1Δ FW	*YEN1* deletion cassette (pAG25)	ATGAAATGACAGTTCTATTGCATTTTACCTACTTGTATATTCTGGATACTGCACAAGAAACAGCTGAAGCTTCGTACGCTGCAG
RHA YEN1Δ RV	*YEN1* deletion cassette (pAG25)	TCCCTCGTTTTCGGCGCGATCAACTGTGGTGGCGGATTTTTTGACGCTGTGCCCGTTAACGCATAGGCCACTAGTGGATCTGATATCATC
YEN1 FW	Verification of *YEN1* deletion, upstream of *YEN1,* with NATMX RV	GTTTTGCTATTTTCAGATCAAATTAC
YEN1 RV	Verification of *YEN1* deletion, downstream of *YEN1* with NATMX FW	CCTCCGCAAAATCTGTGTCACCACC
YEN1 seq FW	Sequencing YEN1	CCTTTTTTAATGGCATTTTCTTTTTCTTATAATG
YEN1 seq RV	Sequencing YEN1	CATAAACTTCATGATTCTTAACATGC
RHA CAJ1Δ FW	*CAJ1* + 300bp upstream deletion cassette (pAG25)	CTTTCGGTTAGCGTTTCAGAAATAAACAATGATGATTAATCCAAAAAATTTCGTATTTGACAGCTGAAGCTTCGTACGCTGCAG
RHA CAJ1Δ RV	*CAJ1* + 300bp upstream deletion cassette (pAG25)	TTTGTAATTGATTGCGCTATATATGAAAAATATACATGAGGCGTTATTAACTTGCTGAGCGCATAGGCCACTAGTGGATCTGATATCATC
CAJ1 FW	Verification of *CAJ1* deletion, upstream of *CAJ1,* with NATMX RV	ATGTATTCTAGACATATCACAGGCA
CAJ1 RV	Verification of *CAJ1* deletion, downstream of *CAJ1* with NATMX FW	AAGAAAAATTGCGTTTGTTAGCTGC
CAJ1 seq FW	Sequencing CAJ1	CTTTGAAGCCATCACCACCG
CAJ1 seq RV	Sequencing CAJ1	ATTTGAGAAGTAAGACAGTG
RHA TPA1Δ FW	*TPA1* deletion cassette (pAG25)	CACTCTTTCTTTTTATTTTTACATTTTTTAATTAAGATCACCAAATATACCGGGAATATACAGCTGAAGCTTCGTACGCTGCAG
RHA TPA1Δ RV	*TPA1* deletion cassette (pAG25)	CTATTATACAACTTTATAAATATCTAAGCTGCCATTTACTTAATAAGACGGGTTAATTGTGCATAGGCCACTAGTGGATCTGATATCATC
TPA1 FW	Verification of *TPA1* deletion, upstream of *TPA1,* with NATMX RV	CAAGAAATACCTAGCTCTTCTAAC
TPA1 RV	Verification of *TPA1* deletion, downstream of *TPA1* with NATMX FW	CTTACTCGAATTTGGATTATGGTAATG
TPA1 seq FW	Sequencing TPA1	AATGAGAGATCCTATGAATTG
TPA1 seq RV	Sequencing TPA1	AGATATGTGACCTCCACGGGAAGAA
RHA HMF1_PET117Δ FW	*HMF1_PET117* deletion cassette (pAG25)	TAAAGCTAAAAAATACGTAGTAAAATCCTAAGTTCTAAAAGACGCTATTAAGACTTGCCCCAGCTGAAGCTTCGTACGCTGCAG

RHA HMF1_PET117Δ RV	*HMF1_PET117* deletion cassette (pAG25)	TTACATATATACAAACAAGAGTAGTCTATAATAGAGAGGTAAACTACTGCCGACAGGTGTGCATAG GCCACTAGTGGATCTGATATCATC
HMF1_PET117 FW	Verification of *HMF1_PET117* deletion, downstream of *HMF1,* with NATMX RV	TAGGGGACCAAGCGCGAGTAACTAC
HMF1_PET117 RV	Verification of *HMF1_PET117* deletion, downstream of *PET117* with NATMX FW	ATTCCGCGCGTTATGATTTAGAAAA
HMF1_PET117 seq FW	Sequencing *HMF1_PET117*	AATGTAATGATGATGTTCGA
HMF1_PET117 seq RV	Sequencing *HMF1_PET117*	CGTCGTTGAATGTCAAATGT
RHA FCY21_FCY22Δ FW	*FCY21_FCY22* deletion cassette (pAG25)	TATTAGCATTGAGGCGCGTGTGCTCGGAAAACCTACCATATTATAAAAGGGCTAACAAATCAGCTGA AGCTTCGTACGCTGCAG
RHA FCY21_FCY22Δ RV	*FCY21_FCY22* deletion cassette (pAG25)	TTATCCATTAAAACCAATCATTTCAATGATTAGCAAAAATATTAATCAATTAACATTTAGGCATAGGC CACTAGTGGATCTGATATCATC
FCY21_FCY22 FW	Verification of *FCY21_FCY22* deletion, upstream of *FCY21,* with NATMX RV	ACCATATTATTTTATTCAAGCTTAT
FCY21_FCY22 RV	Verification of *FCY21_FCY22* deletion, downstream of *FCY22* with NATMX FW	CAAAAGCAAGCCACTGTACATATCC
FCY21_FCY22 seq FW	Sequencing *FCY21_FCY22*	CTTAATTGTTGGTGCCTTTG
FCY21_FCY22 seq RV	Sequencing *FCY21_FCY22*	GGCATACTTTTCTTCGATAT
RHA CEM1Δ FW	*CEM1* deletion cassette (pAG25)	ATTAGCAACTTGAAAGTGACAGGAGCCACAACGGATTAAAATTTAATTTCTAGTAAAGAACAGCTG AAGCTTCGTACGCTGCAG
RHA CEM1Δ RV	*CEM1* deletion cassette (pAG25)	AATAGATAGCATATAATTCAAAAACGATTATAATGGGATATATATATAAGTTTAATAATAGCATAGG CCACTAGTGGATCTGATATCATC
CEM1 FW	Verification of *CEM1* deletion, upstream of *CEM1,* with NATMX RV	CCACAACGGATTAAAATTTAATTTCTAGTAAAG
CEM1 RV	Verification of *CEM1* deletion, downstream of *CEM1* with NATMX FW	TATTGCCATTGCATGTTCAGAGCAT
CEM1 seq FW	Sequencing CEM1	CTGATGTTGACTACGTCAATGCACATG
CEM1 seq RV	Sequencing CEM1	CTTATTGGGTTGGTGAGATCAGTCGTTTG
RHA ALD5_RPS24AΔ FW	*ALD5_RPS24A* deletion cassette (pAG25)	CAAACGTGGTTAAGACAGAAAACTTCTTCACAACATTAACAAAAAGCCAAAGAAGAAGAACAGCTG AAGCTTCGTACGCTGCAG
RHA ALD5_RPS24AΔ RV	*ALD5_RPS24A* deletion cassette (pAG25)	TTTATATATAGAAAACTAAACAATCCGTAAAAATGATTTAACGTTTTGAAGTTAGTTTGCGCATAGGC CACTAGTGGATCTGATATCATC
ALD5_RPS24A FW	Verification of *ALD5_RPS24A* deletion, upstream of *ALD5,* with NATMX RV	CGGTAAAAGCCGGGAACGTGCGTAA
ALD5_RPS24A RV	Verification of *RPS24A* deletion, downstream of *ALD5_RPS24A* with NATMX FW	AAGGCTTCTTTTGTGAGTTCTTTCC
ALD5_RPS24A seq FW	Sequencing RPS24A	AAGTAACTACACTCAAACAA
ALD5_RPS24A seq RV	Sequencing RPS24A	GTGGCCCCGCCTACAGGACG
RHA PTP3Δ FW	*PTP3* deletion cassette (pAG25)	AAAATCCGTATTGTATCTCCCTTCTCCATCAGCAACACAGATCTACTTATCATATAGAACCAGCTGAA GCTTCGTACGCTGCAG
RHA PTP3Δ RV	*PTP3* deletion cassette (pAG25)	CAATTATTAAGGGGTTTCGTATTAATAAAATAGAGATCAAATACATTCATATTAGCCTAAGCATAGG CCACTAGTGGATCTGATATCATC
PTP3 FW	Verification of *PTP3* deletion, upstream of *PTP3,* with NATMX RV	GAGAACACAGCGCAGCGAGAATG
PTP3 RV	Verification of *PTP3* deletion, downstream of *PTP3* with NATMX FW	TACATTTCATCAGTTTTTTCCCGTT
PTP3 seq FW	Sequencing PTP3	GAAATGTTATCGATATTGGCAAGAAG
PTP3 seq RV	Sequencing PTP3	CTAAAAATATTAATTGTCCATGAAATTGGATC
RHA YER076WA_YER077CΔ FW	*YER076WA_YER077C* deletion cassette (pAG25)	AAAATTCAACCATAGAAACTGGAGCGATTGCCAAATCATCGTCCATTTGCCAGAATCCTGCAGCTGA AGCTTCGTACGCTGCAG

RHA YER076WA_YER077CΔ RV	*YER076WA_YER077C* deletion cassette (pAG25)	CTTTATTTTGCCGTAACTAAAAATAAAAACTAAAGAGCAGTGCCCAAGAAGAAGAGGAAAGCATAGGCCACTAGTGGATCTGATATCATC
YER076WA_YER077C FW	Verification of *YER076WA_YER077C* deletion, upstream of *YER076WA,* with NATMX RV	GTAAGAGTTGTGGAAGAGACGGCCC
YER076WA_YER077C RV	Verification of *YER076WA _YER077C* deletion, upstrream of *YER077C* with NATMX FW	GTTATTACTATTGAACCGGGTTTAT
YER076WA_YER077C seq FW	Sequencing *YER076WA_YER077C*	CTATATTGGTTTAAAATGGT
YER076WA_YER077C seq RV	Sequencing *YER076WA_YER077C*	TTTTGAGGTTTTTTCAAATT
RHA YER084WA_ILV1Δ FW	*YER084WA_ILV1* deletion cassette (pAG25)	AGTAATATGTCCCTCTGCCCCTGCCTTTCCCTTTCCAGATGCTGGCATCGAAAGTAAGGCCAGCTGAAGCTTCGTACGCTGCAG
RHA YER084WA_ILV1Δ RV	*YER084WA_ILV1* deletion cassette (pAG25)	TGACGACGTTCATTTTACCTAACAAGTTGTTGCGTAAATTTATAAAGTAAATTGTCGGTTGCATAGGCCACTAGTGGATCTGATATCATC
YER084WA_ILV1 FW	Verification of *YER084WA_ILV1* deletion, upstream of *YER084WA,* with NATMX RV	ATCTCATCCCCAAACTTCTCCCTCC
YER084WA_ILV1 RV	Verification of *YER084WA_ILV1* deletion, downstream of *ILV1* with NATMX FW	AAGAGATTTTGGGTGAAAAATATGA
YER084WA_ILV1 seq FW	Sequencing *YER084WA_ILV1*	TTAGAACCTTCCACATATCG
YER084WA_ILV1 seq RV	Sequencing *YER084WA_ILV1*	GCTTTAGTAGAGTAGCTGAC
RHA AIM10Δ FW	*AIM10* deletion cassette (pAG25)	CTTTCAAAACCACTAATTGACCAACGCCTTTTTTTGAAGATTGACTTCTTCCACATAGCCCAGCTGAAGCTTCGTACGCTGCAG
RHA AIM10Δ RV	*AIM10* deletion cassette (pAG25)	GGTTCTTTTTATTTTGGTGTTATATACATGTATACAAGACGGAAATGGCTTGCTTTGTGGGCATAGGCCACTAGTGGATCTGATATCATC
AIM10 FW	Verification of *AIM10* deletion, upstream of *AIM10,* with NATMX RV	CTTTATAAATTTACGCAACAACTTG
AIM10 RV	Verification of *AIM10* deletion, downstream of *AIM10* with NATMX FW	AACAGCAATAATTCGATTTTGAAGA
AIM10 seq FW	Sequencing AIM10	GACGAAATCAGACCCAGGGGTGGTATCTTAC
AIM10 seq RV	Sequencing AIM10	CATCCACTATAGAAACGTCACTGATTTG
RHA DOT6Δ FW	*DOT6* deletion cassette (pAG25)	CCGACCTCATCGAATAGCTTCCGTGCACGTTCCAGTCTTCCCTCCCTTCTCTGCTCCGTGCAGCTGAAGCTTCGTACGCTGCAG
RHA DOT6Δ RV	*DOT6* deletion cassette (pAG25)	TTTTCTTTTTGTTGTTGATATTTTTTTATTTTTATTTTTTTTTCATTTTAAGTTTTCCCCGCATAGGCCACTAGTGGATCTGATATCATC
DOT6 FW	Verification of *DOT6* deletion, upstream of *DOT6,* with NATMX RV	ATTCGCGCATTGCCTTGCACATCTGTG
DOT6 RV	Verification of *DOT6* deletion, downstream of *DOT6* with NATMX FW	GATGCCGCAACTTTTTGTGAACTTC
DOT6 seq FW	Sequencing DOT6	CACAGTTTAAAAAGGAAAAAAACCGAGTACAACAAAAAC
DOT6 seq RV	Sequencing DOT6	GTGTTGATGTCGATCAAAGCAGTCTTGTTC
RHA PUP3Δ FW	*PUP3* deletion cassette (pAG25)	AGTGCGTTTCTTGCGAATTGTGGTGAAAATATTGAACAGTGAATAAAGCATAAAAAAAAACAGCTGAAGCTTCGTACGCTGCAG
RHA PUP3Δ RV	*PUP3* deletion cassette (pAG25)	CGTTCCGCTGATATTTTGTGTTGGGGTTGTTTTTGGGACCATGAGATTGCCATGAACCGAGCATAGGCCACTAGTGGATCTGATATCATC
PUP3 FW	Verification of *PUP3* deletion, upstream of *PUP3,* with NATMX RV	TTGGAATGCTCAGGGAATGCTC
PUP3 RV	Verification of *PUP3* deletion, downstream of *PUP3* with NATMX FW	TAAGCCTCATGGTAATCAGGGTGAA
PUP3 seq FW	Sequencing PUP3	GTTTCTTGCGAATTGTGGTGAAAATATTG
PUP3 seq RV	Sequencing PUP3	GTATACTACAGCTCCCCAGCCTGATAG

RHA Chr VII		
RHA ATE1_PUF4Δ FW	*ATE1_PUF4* deletion cassette (pAG25)	CATTGCTATTGTTACAAAAGACAAGATAGTCTCAAGATACAACTGAGAAGAAGCACAATTCAGCTGAAGCTTCGTACGCTGCAG
RHA ATE1_PUF4Δ RV	*ATE1_PUF4* deletion cassette (pAG25)	TACAAAAGATATTATATGAGGCTAACGTAAAAAAAGCTAAACTGAGGATATATGTATCAAGCATAGGCCACTAGTGGATCTGATATCATC
ATE1_PUF4 FW	Verification of *ATE1_PUF4* deletion, upstream of *ATE1,* with NATMX RV	CTTTGAGAGTATGGTAAGCTTGATT
ATE1_PUF4 RV	Verification of *ATE1_PUF4* deletion, downstream of *PUF4* with NATMX FW	ATTAACAATATTAACAACAACAACA
ATE1_PUF4 seq FW	Sequencing PUF4	CTCTGTCTATTTCTCTTTGT
ATE1_PUF4 seq RV	Sequencing PUF4	CACTTAAAAATGTTACAGCG
RHA PMA1Δ FW	*PMA1* deletion cassette (pAG25)	AAGAAAATCATTGAAAAGAATAAGAAGATAAGAAAGATTTAATTATCAAACAATATCAATCAGCTGAAGCTTCGTACGCTGCAG
RHA PMA1Δ RV	*PMA1* deletion cassette (pAG25)	AGAAAAATCAAGTTGATTAAAATGTGACAAAAATTATGATTAAATGCTACTTCAACAGGAGCATAGGCCACTAGTGGATCTGATATCATC
PMA1 FW	Verification of *PMA1* deletion, upstream of *PMA1,* with NATMX RV	CTTATGCTCCCCTCCATTAGTTTC
PMA1 RV	Verification of *PMA1* deletion, downstream of *PMA1* with NATMX FW	AGTTGAAATGTGCGTGTTGTGAATT
PMA1 seq FW	Sequencing PMA1	TAATCACGGTGTCGACGACGAAGACAG
PMA1 seq RV	Sequencing PMA1	CAGATTGATCGATTTGCAAGAAACAGTC
RHA UGA1Δ FW	*UGA1* deletion cassette (pAG25)	TGTGTTACATTACAGAAAGAACAGACAAGAAACCGTCAATAAGAAATATAACTAAGAACACAGCTGAAGCTTCGTACGCTGCAG
RHA UGA1Δ RV	*UGA1* deletion cassette (pAG25)	CAAGATACACATATATAAAGACCAAAAAAGGGAACGTGACACGGCCTCGCTAATATACAAGCATAGGCCACTAGTGGATCTGATATCATC
UGA1 FW	Verification of *UGA1* deletion, upstream of *UGA1,* with NATMX RV	GACAAGAAACCGTCAATAAGAAATATAACTAAGAAC
UGA1 RV	Verification of *UGA1* deletion, downstream of *UGA1* with NATMX FW	GTTCCCTGCTATTTTAGAAATTCCA
UGA1 seq FW	Sequencing UGA1	CAAATCTCTTCAATTGCACTTGGTTATAACAACC
UGA1 seq RV	Sequencing UGA1	CAAGGTAATGTCTCTTAGCTTTTGTAAGAAATAC
RHA YGR054WΔ FW	*YGR054W* deletion cassette (pAG25)	ATCTCGATAAAAAAACATAGTACTACAACAAACAGTCAGGTTCAATAACACCTAAACATTCAGCTGAAGCTTCGTACGCTGCAG
RHA YGR054WΔ RV	*YGR054W* deletion cassette (pAG25)	AGTATTATTTGACGTTGTTAATATTTACACAGTTGTATGGATACATCAGTTTCTTCTAGTGCATAGGCCACTAGTGGATCTGATATCATC
YGR054W FW	Verification of *YGR054W* deletion, upstream of *YGR054W,* with NATMX RV	GTCCTTTCTTTATATTCTTTACTTT
YGR054W RV	Verification of *YGR054W* deletion, downstream of *YGR054W* with NATMX FW	GATCTGTTATACAAAACCCTACACG
YGR054W seq FW	Sequencing YGR054W	AAGAAGAACAGGCGGTGGAC
YGR054W seq RV	Sequencing YGR054W	AAATAAGGAAAATGCTCACT
RHA RSC1Δ FW	*RSC1* deletion cassette (pAG25)	GGGAAAATCTGTGAAGTGCCCTAGCTACAAAACAGAGATAAAAAAAATTATATTTCAAGCCAGCTGAAGCTTCGTACGCTGCAG
RHA RSC1Δ RV	*RSC1* deletion cassette (pAG25)	ACAAAATATATATGGTATATGCATGAACATATATAGATACATGTGGTGAATTTATCATGGGCATAGGCCACTAGTGGATCTGATATCATC
RSC1 FW	Verification of *RSC1* deletion, upstream of *RSC1,* with NATMX RV	GTATTTTTTCAATTTTTATACCTAC
RSC1 RV	Verification of *RSC1* deletion, downstream of *RSC1* with NATMX FW	TTTGGTGTCTCTGACGGATGATAAA
RSC1 seq FW	Sequencing RSC1	GAAAAAGGTTAAGTCCAGAAAATATAG
RSC1 seq RV	Sequencing RSC1	CATTAACAGGTATCGTAGGCTCATCTTG

RHA PEF1Δ FW	*PEF1* deletion cassette (pAG25)	CTGAACATGTATGTATACCGTATACCGCATAAAATAACTATAAACCTACACCATTAAACGCAGCTGAAGCTTCGTACGCTGCAG
RHA PEF1Δ RV	*PEF1* deletion cassette (pAG25)	GCCATTGATTGGTTTACCTCTTCTATTTGTATTACGACCAGAAAGAACGAATGGTTTTCAGCATAGGCCACTAGTGGATCTGATATCATC
PEF1 FW	Verification of *PEF1* deletion, upstream of *PEF1,* with NATMX RV	CATTTGAAATGATATTCAGTGGAAG
PEF1 RV	Verification of *PEF1* deletion, downstream of *PEF1* with NATMX FW	CTTCATAATAAAAATTCAATGACGC
PEF1 seq FW	Sequencing PEF1	CATCGCCCTATACAAAAGAGTG
PEF1 seq RV	Sequencing PEF1	CTTGATATCCTTATTATGTAAT
RHA SPR3_ERG25Δ FW	*SPR3_ERG25* deletion cassette (pAG25)	AGAAATAAATAAATAAATAATAAAAACCTAAAATTCCTTTTGCGTCATTGAATTTTTATTCAGCTGAAGCTTCGTACGCTGCAG
RHA SPR3_ERG25Δ RV	*SPR3_ERG25* deletion cassette (pAG25)	TCATTTTATACGTTTTTTTGTTGTTTTTCTTTTTTTTTTTTTTGAAGTATGTTTCTTCTCGCATAGGCCACTAGTGGATCTGATATCATC
SPR3_ERG25 FW	Verification of *SPR3_ERG25* deletion, upstream of *SPR3,* with NATMX RV	CTGGTCGTAATACAAATAGAAGAGG
SPR3_ERG25 RV	Verification of *SPR3_ERG25* deletion, downstream of *ERG25* with NATMX FW	TTCAGATCTGCCAGAAGATGGGTCG
SPR3_ERG25 seq FW	Sequencing SPR3_ERG25	TGTTTTCTTCAAATTTTTTCT
SPR3_ERG25 seq RV	Sequencing SPR3_ERG25	TATATTATATAGAAGTATGC

Allele specific PCR

QTL	Location of 5' end	S288c allele specific primer	ER7a allele specific primer
Chr II	449410 FW	AATTTCGCAATGGAAAACGT	TTTCGCAATGGAAAACG**C**
Chr II	450494 RV	CTTTGCTACGTAAGAAATGGAATTC	CTTTGCTACGTAAGAAATGGAATT**T**
Chr II	463909 FW	CAGTTGGTGAGCTTGTATGACTG	GCAGTTGGTGAGCTTGTATGACT**A**
Chr II	464493 RV	AACAAATCGAACGGTGCG	ATATAAACAAATCGAACGGTGC**A**
Chr II	488838 FW	CATTTATATAATTTTATATAAAGTATAAAAAAAGTGAATAG	CATTTATATAATTTTATATAAAGTATAAAAAAAGTGAATA**C**
Chr II	489262 RV	GTTATATGCTGTGAGCGATGTT	GTTATATGCTGTGAGCGATGT**C**
Chr II	504945 FW	GTACTCAGACGATAACTATGAG	GTACTCAGACGATAACTATGA**A**
Chr II	505383 RV	TGCTTGTTTGTTTATTCTTTA	TGCTTGTTTGTTTATTCTTT**G**
Chr II	536053 FW	GAAAAAAGAAGGCAAGAAAGATGCCAA	GAAAAAAGAAGGCAAGAAAGATGCCA**G**
Chr II	537370 RV	TTAACAAAATTAAATCTGCCAATGTATAGTAA	TTAACAAAATTAAATCTGCCAATGTATAGTA**T**
Chr V	151432 FW	GAAAAAATCAGCAAACAG	GAAA**G**AATCAGCAAACA**A**
Chr V	152071 RV	GGAAATCTAATACTGCTACAAT	GGAAATCTAATACTGCTACAA**A**
Chr V	200052 FW	CGAAAGTGTGATGGATAGGGAT	CGAAAGTGTGATGGATAGGGA**C**
Chr V	200850 RV	TTACACCTTCTTTTGCTGGATGTAC	TTACACCTTCTTTTGCTGGATGTA**T**
Chr V	250546 FW	TCCGGGTAATAGCAGTC	CTTTCCGGGTAATAGCAGT**A**
Chr V	251553 RV	ATATACAAGCTATAAACTCTCCTCT	ATATACAAGCTATAAACTCTCCTCC
Chr V	300653 FW	AAGGATCTCCTGCTTCTCAAGGTAT	AAGGATCTCCTGCTTCTCAAGGTA**C**

Chr V	301089 RV	CATAGTTGGGGCACGTAAGAAC	CCATAGTTGGGGCACGTAAGAA**T**
Chr V	350539 FW	AGCTTTTCGGTGAATTCAGG	AGCTTTTCGGTGAATTCAG**A**
Chr V	351275 RV	TATGCAAAAATCTAAGACAGTGGAT	TATGCAAAAATCTAAGACAGTGGA**C**
Chr V	401541 FW	GTACACCCAGCACAGAACCAA	TACACCCAGCACAGAACCA**G**
Chr V	402144 RV	TGCCCAAGATAGCGCCTACTAC	ATTT**A**CCCAAGATAGCGCCTACTA**T**
Chr V	450243 FW	CCAAAAGGAAGGAACGTCG	CCAAAAGGAAGGAACGTC**A**
Chr V	450819 RV	GCAAGAACAGGGGTTCACCTA	CAAGAACAGGGGTTCA**T**CT**G**
Chr VII	445294 FW	ATGTCTTTTGTATATAATGAAAATTA	ATGTCTTTTGTATATAATGAAAATT**G**
Chr VII	445993 RV	ATTGTTACTTTGGTATGAGG	ATTGTTACTTTGGTATGAG**A**
Chr VII	470321 FW	CAGTACTATCTTTAGAGAATGCA	CAGTACTATCTTTAGAGAATGC**G**
Chr VII	471073 RV	ATATGCATAAAAATTCCTACC	ATATGCATAAAAATTCCTAC**A**
Chr VII	509192 FW	GCCATTTTCTTCAATCTGTTG	AGGCCATTTTCTTCAATCTGTT**A**
Chr VII	509899 RV	GCGTCCGAAGTTGATCATA	GCGTCCGAAGTTGATCAT**C**
Chr VII	542121 FW	TAGTTTAGCTTTCCTAAAAATCGTT	TAGTTTAGCTTTCCTAAAAATCGT**C**
Chr VII	543271 RV	AATCAAGAGGTTGAGATGACAAC	AATCAAGAGGTTGAGATGACAA**T**
Chr VII	584904 FW	TTTGATATATCCTGAAGTTATGTTA	TTTGATATATCCTGAAGTTATGTT**G**
Chr VII	585684 RV	GTTATTTTTCTCTAAGGTGTAATGT	GTTATTTTTCTCTAAGGTGTAATG**C**
Chr VII	620154 FW	CAGAGCCATTTCGGGTGAA	CAGAGCCATTTCGGGTGA**G**
Chr VII	620943 RV	GACGCATTGAACACCTACTCAAGT	GACGCATT**A**AACACCTACTCAAG**C**
Chr VII	660353 FW	AGGTTTCTAACGAGGTTATCACTAC	AGGTTTCTAACGAGGTTATCACTA**G**
Chr VII	661084 RV	CAGCATCAAAATCAGCAGG	CAGCATCAAAATCAGCAG**A**
Chr XI	20444 FW	CGAGATAGACAAAATGTTAAACTTTTCA	CGAGATAGACAAAATGTTA**G**ACTTTTC**G**
Chr XI	21020 RV	AGACATCTCCTAGATCTTGTCTGGC	AGACATCTCCTAGATCTTGTCTGG**T**
Chr XI	51559 FW	TAGATGCATCGCGGATA	TAGATGCATCGCGGAT**G**
Chr XI	52400 RV	AGTGGTTCCAATTGGACGAT	AGTGGTTCCAATTGGACGA**C**
Chr XI	105013 FW	AGTGGTTCCAATTGGACGAT	AGTGGTTCCAATTGGACGA**C**
Chr XI	105793 RV	TGGGTTGTTAATGACAATGTCTAAG	TGGGTTGTTAATGACAATGTCTAA**A**
Chr XI	152360 FW	CTGCACGTAATTGCCGTTT	TGCACGTAATTGCCGTT**G**
Chr XI	153057 RV	GCTATGGGGTAAAAATTTTGGG	GCTATGGGGTAAAAATTTTGG**A**
Chr XI	200890 FW	AGCCACTCTTTTGTCACAAGT	AGCCACTCTTTTGTCACAAG**C**
Chr XI	201371 RV	GATATTTTGCTGCATATCCAAGT	GATATTTTGCTGCATATCCAAG**C**
Chr XI	250265 FW	AGCAGAAGCTGTAACATTACCAC	GAAGCAGAAGCTGTAACATTACCA**T**
Chr XI	250952 RV	CCCTGACATATTCTCAGATGG	AGCCCTGACATATTCTCAGATG**T**
Chr XIV	407152 FW	TTGATTGAAACTTTGCCTGCT	GATTGAAACTTTGCCTGC**G**
Chr XIV	407759 RV	CGTGTTTCACCCATTGATACAA	CGTGTTTCACCCATTGATACA**C**
Chr XIV	425064 FW	CTCTGGATAACGAGTTCTCTAAT	CTCTGGATAACGAGTTCTCTAA**C**
Chr XIV	425763 RV	TGAATAGTCAGGAACAGTTATATTG	ATTGAATAGTCAGGAACAGTTATATT**A**
Chr XIV	435604 FW	ATTTATTGTCAACAAAAAAAGGCAA	ATTTATTGTCAACAAAAAAAGGCA**G**

Chr XIV	436534 RV	ACCAAAGCAAGTACTAAAGGAAATTTT	ACCAAAGCAAGTACTAAAGGAAATTTC
Chr XIV	472094 FW	GCTGGTGGTATTTCAGGTGTTA	CTGGTGGTATTTCAGGTGTT**G**
Chr XIV	472820 RV	GTCTTGTTCTTAAAAGATTGATTGGATAA	GTCTTGTTCTTAAAAGATTGATTGGATA**G**
Chr XIV	474360 FW	CAATCTTGAATTACCCATGAGCTT	CAATCTTGAATTACCCATGAGCT**C**
Chr XIV	475171 RV	TCAATCCATCACTCAATGACCTA	CAATCCATCACTCAATGACCT**G**
Chr XIV	490266 FW	ATATTCAGTTATAGCTAATGAGAGT	ATATTCAGTTATAGCTAATGAGAG**C**
Chr XIV	490813 RV	AAATCGTTGCTCCAGT	GAAATCGTTGCTCCAG**C**
Chr XIV	505824 FW	ATTTTGAAGTGAAAAGTATGATGAA	ATTTTGAAGTGAAAAGTATGATGA**G**
Chr XIV	506323 RV	TGAGAGACCACACAAGAGC	TTCTGAGAGACCACACAAGAG**T**
Chr XIV	541371 FW	ATTTCTCCAGGCAGAAAACCTA	TCTCCAGGCAGAAAACCT**G**
Chr XIV	542442 RV	GGATTCAACCGGCACTG	GGGATTCAACCGGCACT**A**
Chr XIV	555458 FW	AGATTAGCTTCACTGAGTAAGG	GATTAGCTTCACTGAGTAAG**C**
Chr XIV	556379 RV	GTTTTCTTGATCTATAAGGGTATAA	GTTTTCTTGATCTATAAGGGTATA**G**

Supplementary table S5. Plasmids used in this study.

Plasmid	Description	Reference
pUG6	*E. coli* vector, Amp+, loxP-KanMX6-loxP disruption cassette	(Guldener et al. 1996)
pAG25	yeast shuttle vector, Amp+, NatMX4 disruption cassette	(Goldstein and McCusker 1999)
pFL39 GAL1 HO KanMX	vector containing HO gene	Lab stock
pBluescriptII_NAT1_GIN11_SSK	phagmid vector, Amp+, disruption cassette with selectable NatMX4 and counter-selectable GIN11 marker (Akada et al. 1999; Akada et al. 2002)	(Hubmann et al. 2013)

Akada R, Hirosawa I, Kawahata M, Hoshida H, Nishizawa Y. 2002. Sets of integrating plasmids and gene disruption cassettes containing improved counter-selection markers designed for repeated use in budding yeast. *Yeast* **19**(5): 393-402.

Akada R, Matsuo K, Aritomi K, Nishizawa Y. 1999. Construction of recombinant sake yeast containing a dominant FAS2 mutation without extraneous sequences by a two-step gene replacement protocol. *Journal of bioscience and bioengineering* **87**(1): 43-48.

Goldstein AL, McCusker JH. 1999. Three new dominant drug resistance cassettes for gene disruption in Saccharomyces cerevisiae. *Yeast* **15**(14): 1541-1553.

Guldener U, Heck S, Fielder T, Beinhauer J, Hegemann JH. 1996. A new efficient gene disruption cassette for repeated use in budding yeast. *Nucleic acids research* **24**(13): 2519-2524.

Hubmann G, Foulquie-Moreno MR, Nevoigt E, Duitama J, Meurens N, Pais TM, Mathe L, Saerens S, Nguyen HT, Swinnen S et al. 2013. Quantitative trait analysis of yeast biodiversity yields novel gene tools for metabolic engineering. *Metabolic engineering* **17**: 68-81.
